# Transitional care programs for older adults moving from hospital to home in Canada: A systematic review of text and opinion

**DOI:** 10.1371/journal.pone.0307306

**Published:** 2024-07-18

**Authors:** Brittany V. Barber, Emily E. Gregg, Emily K. Drake, Marilyn Macdonald, Madison Hickey, Chloe Flynn, Elaine Moody, Sarah M. Gallant, Erin McConnell, Lori E. Weeks

**Affiliations:** 1 School of Nursing, Faculty of Health, Dalhousie University, Halifax, Nova Scotia, Canada; 2 Aligning Health Needs and Evidence for Transformative Change: A JBI Centre of Excellence, Dalhousie University, Halifax, Nova Scotia, Canada; 3 Department of Nursing & Health Sciences, University of New Brunswick, Saint John, New Brunswick, Canada; 4 University of New Brunswick (UNB) Saint John Collaboration for Evidence-Informed Healthcare: A JBI Centre of Excellence, University of New Brunswick, Saint John, New Brunswick, Canada; Jashore University of Science and Technology (JUST), BANGLADESH

## Abstract

**Background:**

Investing in transitional care programs is critical for ensuring continuity of health and coordinated care for older adults transitioning across health settings. However, literature delineating the scope of transitional care programs across Canada is limited. The aim of this systematic review of text and opinion is to characterize Canadian transitional care programs for older adults transitioning from hospital to home.

**Methods:**

Following JBI guidelines for systematic review of text and opinion, we conducted a search of Canadian grey literature sources published online between 2016 to 2023. A 3-phase search was undertaken for: 1) Canadian databases and organizational websites; 2) advanced Google search of national sources and news media reports; and 3) advanced Google search of provincial/territorial sources. Two reviewers independently screened sources for eligibility against inclusion criteria. Data were extracted by one reviewer and verified by a second. Textual data were extracted from multiple sources to characterize each transitional care program.

**Results:**

Grey literature search produced a total of 17,092 text and opinion sources, identifying 119 transitional care programs in Canada. Model of care was a key characteristic defining the design and delivery of transitional care programs within community (n = 42), hospital (n = 45), and facility-based (n = 32) settings. Programs were characterized by goal, population and eligibility, setting and length of program, intervention and services, and healthcare team members. Patient, caregiver, and health system outcomes were reported for 18 programs. The province of Ontario has the most transitional care programs (n = 84) and reported outcomes, followed by British Columbia (n = 10).

**Conclusions:**

Characterizing transitional care programs is important for informing health services planning and scaling up of transitional care program models across Canada. Recognizing transitional care programs as a core health service is critical to meet the health care needs of older adults at the right time and place.

**Trial registration:**

PROSPERO ID 298821.

## Introduction

The proportion of adults aged 60 and older is expected to double globally, from 12% in 2015 to 22% by 2050 [1]. This projected demographic shift presents challenges for countries to strategically prepare both health and social systems to meet the needs of the steadily aging population. The growing population of older adults in the next 20 years is particularly important for Canada, [2] due to the significant increase expected in the number of older adults 85 and over (from approximately 871,000 in 2021 to 3 million by 2050) [3]. As populations of older adults aged 85 and older experience increasingly complex health care needs, pressures on health and home care sectors will likely be exacerbated [3]. Ensuring older adults receive high quality care at the appropriate time and place is a priority for Canadian clinicians, researchers, and policy makers.

Attention to transitional care services is crucial for delivering high-quality care for older adults. Transitional care encompasses interventions designed to ensure both coordination and continuity of care as patients move between different locations or levels of care within the same location [4, 5]. Increased emphasis has been placed on the importance of improving transitions within and between health settings, [6] suggesting transitional care programs warrant recognition as a core health service. One challenge to identifying, evaluating, and synthesizing international evidence related to transitional care programs is the diverse range of terminology adopted throughout the literature. Depending on the country of origin, transitional care programs may be referred to as intermediate care models, sub-acute care, post-acute care, or alternate care units [7]. In Canada, transitional care program appears to be the predominant title, [7] and will be the term adopted for this review. For the purposes of this review, an older adult is defined as an individual 65 years or older, [8] and for Indigenous populations an older adult is defined as an individual 55 years or older [9, 10]. Indigenous populations in Canada continue to experience direct impacts of colonialism and multiple health disparities such as lower socioeconomic status, worse health outcomes, resulting in shorter life expectancy from suboptimal social, physiological and emotional support towards a healthy aging process [9, 10].

Health system challenges associated with older adults designated “alternate level of care” (ALC) in acute care hospitals is a significant contributing factor to unfavorable health outcomes and increased costs of care [11]. Older adults in hospital receive ALC status when they no longer require medical care but are not yet ready to return home or are awaiting placement in an alternate location [12]. While ALC-designated individuals wait in hospital until they can be transitioned to their final discharge destination, an acute care hospital bed remains occupied. Depending on the ALC occupancy rate within a given facility, patient flow may be significantly decreased or halted due to a lack of available hospital beds. Interruptions in patient flow contributes to overcrowded emergency departments, increased wait times, and ultimately, reduced access to essential care [11]. The resulting systemic impact of the ALC challenge on patient flow in hospitals is overwhelming and even more concerning is the subsequent effect on individual access to care.

In Canada, excluding the province of Quebec, 16% of patient days in hospital were associated with ALC status in 2022–2023 (ranging from 6.3% in Nunavut to 25.4% in Prince Edward Island) [13]. ALC patient days were highest in Atlantic provinces, with 20% in Nova Scotia and New Brunswick, 23.5% in Newfoundland and Labrador, and 25.4% in Prince Edward Island [13]. Although a significant portion of ALC designated individuals are older adults awaiting residential long-term care placement, it is important to acknowledge the ALC population with a discharge destination of home who experience a delay in discharge. The delay may be due to reasons such as time required to organize home care or other community services, essential home renovations and supports to live independently, home care capacity to admit new clients, or additional rehabilitation to improve functioning such as achieving activities of daily living [11]. Thus, while increasing available residential long-term care beds plays a critical role in addressing the ALC crisis, a more comprehensive and multi-faceted approach is required to improve older adult health care services.

Older adults that remain in hospital when acute-care treatment is no longer required often experience detrimental health effects, [14–17] including unnecessary stress for families and caregivers in relation to lack of communication and uncertainty around discharge planning [14–17]. Furthermore, a lack of resources within acute care hospitals means older adult patients are unlikely to receive the rehabilitative programming they require to improve or maintain cognitive functioning and mobility after an acute illness [14, 16, 18]. These system-level challenges suggest the infrastructure, care model, and human resources of acute care hospitals are not designed to meet the specific needs of older adults designated as ALC patients [14]. An alternative setting or program designed to meet the unique care, rehabilitative needs, and discharge goals for older adult ALC patients is ideal for promoting health and wellness of older adult populations.

There is a growing evidence base to support the role of transitional care programs for older adults moving from hospital to home. The diversity of transitional care interventions, location of program delivery, and specific program characteristics across countries is highlighted within peer-reviewed literature [19–22]. For instance, core components of transitional care programs have been found to include: assessment; care planning and monitoring; discharge planning; and patient, family, and staff education [7]. Despite core program components remaining relatively similar between countries and health care systems (i.e., Europe, U.S.A, Australia) a recent scoping review highlights the wide range of health system program delivery and intervention services [7]. Such heterogeneity makes it challenging to compare results from systematic reviews spanning diverse health care systems and transitional care models of care and intervention services.

In terms of effectiveness of transitional care programs, the majority of research has focused on health care service utilization [20, 23, 24] and cost-effectiveness [25–28]. Investigation into other patient-focused outcomes, such as improving functional status towards activities of daily living and successful discharge home are demonstrated for adults participating in facility-based transitional care programs outside hospital settings [29, 30]. However, transitional care program impact on family and caregiver outcomes such as caregiver burnout are largely unexplored [31].

The majority of research investigating transitional care programs was conducted outside Canada [7, 29, 31, 32]. Despite limited peer-reviewed literature, there is experiential knowledge Canadian transitional care programs exist [33]. Further research is needed to better understand the scope of Canadian transitional care programs, including characteristics of model of care, setting, and health care professional teams across provincial and territorial regions. In addressing this knowledge gap, we aimed to conduct a systematic review of text and opinion sources to characterize the types of transitional care programs that exist across Canada. Building the evidence base of the types and characteristics of Canadian transitional care programs (or lack thereof) can help inform provincial/territorial decision makers and health system leaders to address gaps in service delivery with greater investments in health care resources for older adults. Further, information about the scope of Canadian transitional care programs can strengthen knowledge of health system improvements internationally, such as for countries aiming to improve transitional care services for aging populations, particularly in countries with publicly funded health systems like Canada. This review was conducted following a previously published protocol [34].

### Review questions

The primary aim of this systematic review of text and opinion was to identify and characterize transitional care programs that exist across Canada to support older adults with moving from hospital to home. We aimed to achieve this by answering the following research questions:

1. What transitional care programs exist across Canada for older adults to support moving from hospital to home?
2. What are the characteristics and reported outcomes of these programs?

### Inclusion criteria

#### Participants

Sources focusing on older adults transitioning from hospital-based acute care to another care setting, such as the person’s home in the community or short-term care settings (i.e., rehabilitation services in long-term care facilities and hospital settings), were considered for inclusion. This review focused on the types of transitional care programs providing care services to older adults with any medical condition, morbidity, cognition level, regardless of whether they have been in acute care previously. Sources focusing on adults younger than 65 were excluded, or 55 if the source focused on Indigenous populations.

### Phenomena of interest

The phenomenon of interest was transitional care programs supporting older adults transitioning from hospital to home, including the person’s home in the community and other short-term care settings including programs operating from hospital and long-term care facilities. Characteristics of transitional care programs include services offered, types of care settings, characteristics of healthcare teams, patient populations served, integration with other healthcare services, resources and services provided for unpaid caregivers, and the impact of the program. Terminology used to describe transitional care programs and related interventions are inconsistent across regions and care settings in Canada [33]. Interchangeable terms identified in the literature on transitional care programs were included in this review such as, “intermediate care programs,” “reintegration programs,” “subacute care,” “reactivation programs,” “short-term transitional care programs,” “skilled nursing facilities,” and “post-acute care.” Transitional care interventions and programs focused on transitioning seniors to long-term residential care facilities were excluded from this review. Care programs providing short-term respite and observation stays were also excluded.

### Context

The focus of this review was on Canadian transitional care programs delivered by provincial/territorial health systems and services. Canada consists of 10 provinces and 3 territories with diverse geographic characteristics (e.g., urban vs. rural), local cultural/subcultural contexts, and varying sociodemographic factors (e.g., race, age, ethnicity, language, gender identities). Sources focusing on transitional care programs outside of Canada were excluded.

### Types of publications

This review considered grey literature of text and opinion including white papers, internal healthcare documents, websites, procedures, policies, evaluations, government documents, expert reports, policy literature, working papers, newsletters, and media sources.

## Methods

This systematic review was conducted in accordance with JBI methodology for systematic reviews of text and opinion, [35] and the Preferred Reporting Items for Systematic Reviews and Meta-Analysis checklist for systematic reviews [36] (S1 Appendix). Ethics approval was not required for this systematic review because all data sources are secondary and already in the public domain.

### Search strategy

A list of Canadian sources of grey literature and a comprehensive search strategy was developed by team members in conjunction with two health science librarians (S2 Appendix). A 3-phase search strategy was undertaken by two researchers independently. First, an initial limited search of Canadian grey literature databases and organizational websites was conducted to identify sources on the topic of transitional care programs. Canadian databases and organizational websites of grey literature included Government and Legislative Libraries Online Publication Portal (GALLOP), Canadian Institute for Health Information (CIHI), Canadian Nurses Association, Theses Canada Portal, Google Programmable Search Engine for Canadian Federal Documents, and Canadian Agency for Drug and Technologies in Health (CADTH). Next, an advanced incognito Google search of Canadian resources and Canadian news media reports from ProQuest and Nexis Uni was conducted. Lastly, an advanced Google search of all 10 provinces and 3 territories using provincial/territorial search term was completed to retrieve information about local transitional care programs, including local pilot projects, health region-specific programs and provincial/territorial organizations. A list of keywords and index terms are provided (S2 Appendix). A forward search of identified transitional care programs was conducted to gather additional text and opinion sources that our search may have missed.

All grey literature sources published since 2016 were included in this review. In 2016, the World Health Organization introduced a framework on Integrated, People-centred Health Services during the Sixty-Ninth World Health Assembly [6]. This framework identified coordination of services within and across sectors as a key strategy for enhancing health systems, particularly during care transitions. Since this framework was introduced, there has been an increase in efforts to improve health outcomes and experiences of people transitioning across health settings. Grey literature sources published in English or French were considered for inclusion, as Canada’s co-official languages. Sources published in French and unavailable online in English were translated by team members with French-language skills. The initial search was conducted from July to October 2022, and updated from October to November 2023 to verify source links and identify new eligible programs; no additional transitional care programs were located.

### Study selection

**Fig 1:**
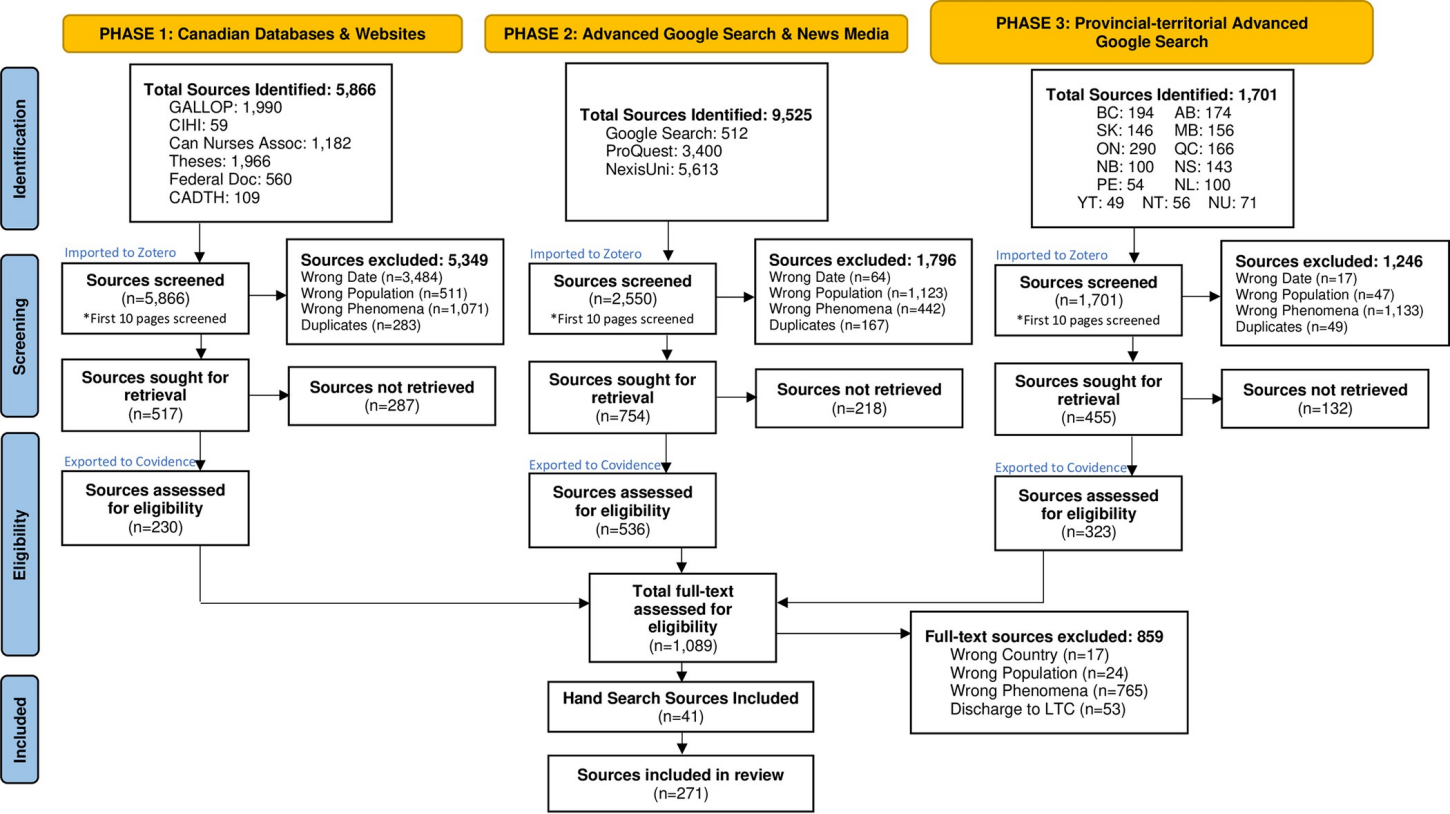
PRISMA flow chart. *From*: Page MJ, McKenzie JE, Bossuyt PM, Boutron I, Hoffmann TC, Mulrow CD, et al. The PRISMA 2020 statement: an updated guideline for reporting systematic reviews. BMJ 2021;372:n71. doi: 10.1136/bmj.n71. For more information, visit: http://www.prisma-statement.org/.

At the time the search was conducted, all potential sources were screened against inclusion criteria. Most grey literature sources did not have an abstract for screening, except sources within the Theses Canada Portal. Therefore, titles and abbreviated descriptions were used to screen sources by two independent reviewers (BB and EKD) for assessment against inclusion criteria using a screening tool (S3 Appendix). Identified sources from each search were imported to Zotero and duplicates removed. Sources sought for retrieval were uploaded to Covidence, [37] to facilitate full-text review. Any conflicts that arose between reviewers were resolved through discussion. Multiple sources (i.e., websites, news media, reports) related to each transitional care program were included in the final review and assessed to gather as much information as possible about each transitional care program. The results of the search and included sources are presented in the PRISMA flow diagram Fig 1. Full-text sources that did not meet inclusion criteria were excluded, with reasons for exclusion provided (Fig 1).

### Assessment of methodological quality

Grey literature sources selected for inclusion were assessed by two independent reviewers (BB and EG) using the JBI critical appraisal checklist (2020) for text and opinion [35]. Results of the critical appraisal are reported in Table 1. Sources were not excluded based on their quality. During critical appraisal, reviewers identified questions 4, 5, and 6 were unsuitable for text and opinion sources included in this review (Question 4: Is the stated position the result of an analytical process and is there logic in the opinion expressed? Question 5: Is there reference to the extant literature? and Question 6: Is any incongruence with the literature/sources logically defended?). Grey literature sources could not be assessed for logic in opinion expressed, were not reporting on empirical research involving reference to extant literature, and sources did not report any incongruence with literature sources. Furthermore, reviewers considered all organizations delivering transitional care programs for older adults to have standing in the field (Question 2: Does the source of opinion have standing in the field of expertise).

**Table 1:** Critical appraisal of eligible sources.

Province/ Program Name/ Author/ (Year)	Q1	Q2	Q3	Q4	Q5	Q6	Total Score
Ontario							
ON/	Y	Y	Y	N/A	N/A	N/A	3
Let’s Go Home (LEGHO)/ One Care Support/ (2022) (42)							
ON/	Y	Y	Y	N/A	N/A	N/A	3
Richview Community Care Services/ Toronto Central Healthline/ (2022) (43)							
ON/	Y	Y	Y	N/A	N/A	N/A	3
Home Concierge—Post-operative care/ Home Concierge/ (2022) (44)							
ON/	Y	Y	Y	N/A	N/A	N/A	3
Home at Last/ West Neighbourhood Group/ (2022) (45)							
ON/	Y	Y	Y	N/A	N/A	N/A	3
Integrated Comprehensive Care Program/ St. Joseph’s Healthcare Hamilton/(2018) (46)							
ON/	Y	Y	Y	N/A	N/A	N/A	3
Bayshore @ Home/ Bayshore Healthcare/ (2022) (47)							
ON/	Y	Y	Y	N/A	N/A	N/A	3
Aghabi Place—Bridges to Care Program/ Able Living/ (2022) (48)							
ON/	Y	Y	Y	N/A	N/A	N/A	3
Southlake@Home/ Southlake Regional Health Centre/ (2020) (49)							
ON/	Y	Y	Y	N/A	N/A	N/A	3
C-Care/ C-Care Health Services/ (2022) (50)							
ON/	Y	Y	Y	N/A	N/A	N/A	3
Comfort Keepers Post Hospital Care/ Comfort Keepers/ (2022) (51)							
ON/	Y	Y	Y	N/A	N/A	N/A	3
Spectrum Hospital to Home Program/ Spectrum Health Care/ (2022) (52)							
ON/	Y	Y	Y	N/A	N/A	N/A	3
Scarborough Health Network Transitional Care Program [SHN@home]/ Scarborough Health Network/ (2023) (53)							
ON/	Y	Y	Y	N/A	N/A	N/A	3
SE Care Transitions: Reactivation Care/ Saint Elizabeth Health Care/ (2022) (54)							
ON/	Y	Y	Y	N/A	N/A	N/A	3
Post Hospital Transition Care/ Global Health Care Services/ (2022) (55)							
ON/	Y	Y	Y	N/A	N/A	N/A	3
MSH Care@Home/ Eastern York Region North Durham/ (2022) (56)							
ON/	Y	Y	Y	N/A	N/A	N/A	3
Care@Home/ Eastern York Region North Durham/ (2022) (57)							
ON/	Y	Y	Y	N/A	N/A	N/A	3
KHSC@Home/ Kingston Health Sciences Centre/ (2021) (58)							
ON/	Y	Y	Y	N/A	N/A	N/A	3
Dynamic Home Care Services/ Kingston Health Sciences Centre/ (2022) (59)							
ON/	Y	Y	Y	N/A	N/A	N/A	3
PATH: Priority Support to Transition Home/ Timiskaming Home Support/ (2022) (60)							
ON/	Y	Y	Y	N/A	N/A	N/A	3
Donato House & Post Stroke Transitional Care Program/ ICAN Independence Centre Network/ (2017) (61)							
ON/	Y	Y	Y	N/A	N/A	N/A	3
PHARA Transitional Care Program/ PHARA Independence and Housing/ (2022) (62)							
ON/	Y	Y	Y	N/A	N/A	N/A	3
Frail Seniors Transition to Home Program/ West Park Healthcare Centre/ (2022) (63)							
ON/	Y	Y	Y	N/A	N/A	N/A	3
LOFT Specialized Support: Transition from hospital/ LOFT Community Service/ (2022) (64)							
ON/	Y	Y	Y	N/A	N/A	N/A	3
The Key: Post-Hospital Care/ Home Care Assistance/ (2022) (65)							
ON/	Y	Y	Y	N/A	N/A	N/A	3
One Care: Home at Last/ One Care Home and Community Support Services/ (2022) (66)							
ON/	Y	Y	Y	N/A	N/A	N/A	3
Right at Home: Hospital to Home Program (RightTransitions)/ Right at Home Canada/ (2022) (67)							
ON/	Y	Y	Y	N/A	N/A	N/A	3
Divine Care—Transitional Care Services/ Divine Home Care/ (2022) (68)							
ON/	Y	Y	Y	N/A	N/A	N/A	3
Canes@Home Transitional Care/ Canes Community Care/ (2020) (69)							
ON/	Y	Y	Y	N/A	N/A	N/A	3
Transitional Rapid Access Care Coordination (TRACC)/ Sinai Health/ (2022) (70)							
ON/	Y	Y	Y	N/A	N/A	N/A	3
Emergency Department Diversion Program/ Grand River Hospital/ (2021) (71)							
ON/	Y	Y	Y	N/A	N/A	N/A	3
Circle of Care/ Sinai Health/ (2022) (72)							
ON/	Y	Y	Y	N/A	N/A	N/A	3
Southlake Transitional Care/ Southlake Regional Health Centre/ (2019) (73)							
ON/	Y	Y	Y	N/A	N/A	N/A	3
Reactivation Care Unit/ Northeast Specialized Geriatric Centre/ (2023) (74)							
ON/	Y	Y	Y	N/A	N/A	N/A	3
St. Joseph’s Continuing Care Centre/ St. Joseph’s Health Centre of Sudbury/ (2019) (75)							
ON/	Y	Y	Y	N/A	N/A	N/A	3
Acute Care of the Elderly Unit (ACE)/ Michael Garron Hospital/ (2023) (76)							
ON/	Y	Y	Y	N/A	N/A	N/A	3
Short-term Inpatient Rehab/ Michael Garron Hospital/ (2023) (77)							
ON/	Y	Y	Y	N/A	N/A	N/A	3
Centralized Care and Transitions (CCaTT)/ Hamilton Health Sciences/ (2023) (78)							
ON/	Y	Y	Y	N/A	N/A	N/A	3
Hospital Outreach Team/ Hamilton Health Sciences/ (2019) (79)							
ON/	Y	Y	Y	N/A	N/A	N/A	3
Geriatric Rehab Unit (M3)/ Hamilton Health Sciences/ (2023) (80)							
ON/	Y	Y	Y	N/A	N/A	N/A	3
Ottawa Hospital Rehabilitation Centre (TOHRC)/ The Ottawa Hospital/ (2023) (81)							
ON/	Y	Y	Y	N/A	N/A	N/A	3
Specialized Seniors Care Inpatient Unit/ Royal Victoria Regional Health Centre/ (2021) (82)							
ON/	Y	Y	Y	N/A	N/A	N/A	3
Transitional Care Inpatient Unit/ Royal Victoria Regional Health Centre/ (2021) (83)							
ON/	Y	Y	Y	N/A	N/A	N/A	3
Sudbury Health Sciences COACH/ Health Sciences North/ (2023) (84)							
ON/	Y	Y	Y	N/A	N/A	N/A	3
West Haldimand Transitional Care Bed Program/ Able Living/ (2022) (85)							
ON/	Y	Y	Y	N/A	N/A	N/A	3
Willett Transitional Care Bed Program/ Able Living/ (2022) (86)							
ON/	Y	Y	Y	N/A	N/A	N/A	3
St. Joseph’s Parkwood Hospital Complex Care and Transitional Care Unit/ St. Joseph’s Hospital/ (2022) (87)							
ON/	Y	Y	Y	N/A	N/A	N/A	3
St. Joseph’s Geriatric Rehabilitation Unit/ St Joseph’s Hospital/ (2022) (88)							
ON/	Y	Y	Y	N/A	N/A	N/A	3
Geriatric Engagement and Reintegration Unit/ Ross Memorial Hospital/ (2022) (89)							
ON/	Y	Y	Y	N/A	N/A	N/A	3
Ontario Shores Geriatric Transitional Unit/ Ontario Shores Centre for Mental Health Services/ (2021) (90)							
ON/	Y	Y	Y	N/A	N/A	N/A	3
Halton Complex Transitional Care (CTC)/ Halton Health Care/ (2022) (91)							
ON/	Y	Y	Y	N/A	N/A	N/A	3
Centenary Complex Continuing Care Unit/ Scarborough Health Network/ (2023) (92)							
ON/	Y	Y	Y	N/A	N/A	N/A	3
Day Therapy Transitional Program/ St. Joseph’s Healthcare Hamilton/ (2022) (93)							
ON/	Y	Y	Y	N/A	N/A	N/A	3
Reactivation Care Centre/							
Central LHIN Hospitals Collaborative/ (2022) (94)							
*Locations at Humber River Health; Trillium Health Partners; Sunnybrook Health Sciences; St Joseph’s Health Centre; William Osler Health System							
ON/	Y	Y	Y	N/A	N/A	N/A	3
Ben & Hilda Katz ACE Unit/ Sinai Health/ (2022) (95)							
ON/	Y	Y	Y	N/A	N/A	N/A	3
Bruyere Transitional Care Program/ Bruyere/ (2022) (96)							
ON/	Y	Y	Y	N/A	N/A	N/A	3
Hennick Bridgepoint/ Sinai Health/ (2022) (97)							
ON/	Y	Y	Y	N/A	N/A	N/A	3
St. Michael’s Unity Health ACE Unit/ Unity Health Toronto/ (2022) (98)							
ON/	Y	Y	Y	N/A	N/A	N/A	3
Seniors and Rehabilitation Day Hospital/ Trillium Health Partners/ (2022) (99)							
ON/	Y	Y	Y	N/A	N/A	N/A	3
Assess & Restore Program/ Toronto Grace Health Centre/ (2022) (100)							
ON/	Y	Y	Y	N/A	N/A	N/A	3
RECOVER Program/ Toronto Grace Health Centre/ (2022) (101)							
ON/	Y	Y	Y	N/A	N/A	N/A	3
Satellite Health Facility/ St. Joseph’s Health- care Hamilton/ (2022) (102)							
ON/	Y	Y	Y	N/A	N/A	N/A	3
Restorative Transitional Care/ Providence Care Centre/ (2021) (103)							
ON/	Y	Y	Y	N/A	N/A	N/A	3
Care First Transitional Care Centre/ Care First/ (2022) (104)							
ON/	Y	Y	Y	N/A	N/A	N/A	3
Integrated Transitional Services Program/ Toronto Grace Health Centre/ (2022) (105)							
ON/	Y	Y	Y	N/A	N/A	N/A	3
Pine Villa Transitional Care/ Pine Villa/ (2022) (106)							
ON/	Y	Y	Y	N/A	N/A	N/A	3
Villa Pugliese Assisted Living/ Villa Pugliese/ (2022) (107)							
ON/	Y	Y	Y	N/A	N/A	N/A	3
Integrated Care Solutions/ Bayshore Healthcare/ (2022) (108)							
ON/	Y	Y	Y	N/A	N/A	N/A	3
Reintegration Care Unit/ Les Centres d’Accueil Héritage (CAH)/ (2022) (109)							
ON/	Y	Y	Y	N/A	N/A	N/A	3
Hillcrest Reactivation Centre/ University Health Network/ (2022) (110)							
ON/	Y	Y	Y	N/A	N/A	N/A	3
Binbrook Transitional Care Bed Program/ AbleLiving/ (2022) (111)							
ON/	Y	Y	Y	N/A	N/A	N/A	3
Helping Hands Orillia Transitional Bed Program/ Helping Hands/ (2022) (112)							
ON/	Y	Y	Y	N/A	N/A	N/A	3
Transitional Care Units/ VHA Home Health Care/ (2022) (113)							
ON/	Y	Y	Y	N/A	N/A	N/A	3
Transitional Care Sites/ Canes Community Care/ (2020) (114)							
ON/	Y	Y	Y	N/A	N/A	N/A	3
RVH-IOOF Patient Flow Program/ Royal Victoria Regional Health Centre/ (2021) (115)							
ON/	Y	Y	Y	N/A	N/A	N/A	3
Transitional Behavioral Support Unit/ Baycrest/ (2023) (116)							
ON/	Y	Y	Y	N/A	N/A	N/A	3
Queen’s Estate Transitional Care Rehab Unit/ Queens Estate/ (2019) (117)							
ON/	Y	Y	Y	N/A	N/A	N/A	3
Yee Hong Centre for Geriatric Care/ Indus Community Services/ (2018) (118)							
ON/	Y	Y	Y	N/A	N/A	N/A	3
Sunrise Short Stay Support/ Eastern York Region North Durham Sunrise Senior Living/ (2022) (119)							
*Facilities exist in QC, ON							
ON/	Y	Y	Y	N/A	N/A	N/A	3
Perley Health Sub-Acute Care for Frail Elderly [SAFE]/ Perley Health/ (2021) (120)							
ON/	Y	Y	Y	N/A	N/A	N/A	3
Caledon Transitional Care Program/ Caledon Community Services/ (2022) (121)							
ON/	Y	Y	Y	N/A	N/A	N/A	3
Transitional Care Unit: Windsor Retirement Residence/ Kingston Health Sciences Centre/ (2021) (122)							
ON/	Y	Y	Y	N/A	N/A	N/A	3
Rubidge Retirement Residence Transitional Care Unit/ Peterborough Regional Health Centre/ (2020) (123)							
ON/	Y	Y	Y	N/A	N/A	N/A	3
Rekai Centre Transitional Care Unit/ Sinai Health/ (2022) (124)							
ON/	Y	Y	Y	N/A	N/A	N/A	3
Transitional Care Beds/ Niagara Gardens Retirement Manor/ (2022) (125)							
British Columbia							
BC/	Y	Y	Y	N/A	N/A	N/A	3
Personalized Support & Stabilization Team Plus (PSS+) at Robert & Lily Lee Family Community Health Centre/ Vancouver Coastal Health/ (2023) (126)							
BC/	Y	Y	Y	N/A	N/A	N/A	3
Transition Services Team (TST) Vancouver General Hospital/ Vancouver Coastal Health/ (2023) (127)							
BC/	Y	Y	Y	N/A	N/A	N/A	3
Transition Services at Koerner Pavilion/ Vancouver Coastal Health/ (2023) (128)							
BC/	Y	Y	Y	N/A	N/A	N/A	3
Convalescent Care Program/ Fraser Health/ (2023) (129)							
BC/	Y	Y	Y	N/A	N/A	N/A	3
Convalescent Care Program/ Interior Health/ (2023) (130)							
BC/	Y	Y	Y	N/A	N/A	N/A	3
Providence Care Centre/ Providence Care/ (2022) (131)							
BC/	Y	Y	Y	N/A	N/A	N/A	3
Glengarry Transitional Care Unit/ Island Health/ (2023) (132)							
BC/	Y	Y	Y	N/A	N/A	N/A	3
Short Term Enablement and Planning Suites (STEPS)/ Island Health/ (2022) (133)							
BC/	Y	Y	Y	N/A	N/A	N/A	3
Evergreen House/ Vancouver Coastal Health/ (2023) (134)							
BC/	Y	Y	Y	N/A	N/A	N/A	3
Glenwood Care Centre/ Fraser Health/ (2023) (135)							
Alberta							
AB/	Y	Y	Y	N/A	N/A	N/A	3
Alberta Health Services Self-Managed Care Program/ Harmony Caregiving/ (2023) (136)							
AB/	Y	Y	Y	N/A	N/A	N/A	3
Post-Hospital Care/ Comfort Keepers/ (2023) (137)							
AB/	Y	Y	Y	N/A	N/A	N/A	3
Post-Hospital Care/ Care West AB/ (2020) (138)							
AB/	Y	Y	Y	N/A	N/A	N/A	3
Hope 4 Life/ Hope 4 Life Home Care/ (2017) (139)							
AB/	Y	Y	Y	N/A	N/A	N/A	3
Rehabilitation and Enhanced Community Transition Program/ Home Care Assistance/ (2022) (140)							
Saskatchewan							
SK/	Y	Y	Y	N/A	N/A	N/A	3
Kensington Transitional Beds/ Kensington Gentle Care Home/ (2023) (141)							
SK/	Y	Y	Y	N/A	N/A	N/A	3
Convalescent Care/ William Booth Special Care Home/ (2021) (142)							
Manitoba							
MB/	Y	Y	Y	N/A	N/A	N/A	3
Priority Home Rapid Response Nursing Team/ Winnipeg Regional Health Authority							
(2017) (143)							
MB/	Y	Y	Y	N/A	N/A	N/A	3
Priority Home/ Winnipeg Regional Health Authority/ (2017) (144)							
MB/	Y	Y	Y	N/A	N/A	N/A	3
Prairie Mountain Health Transitional Care/ Prairie Mountain Health/ (2023) (145)							
MB/	Y	Y	Y	N/A	N/A	N/A	3
Misericordia Transitional Care Unit (Restorative Care)/ Misericordia Health Centre/ (2023) (146)							
MB/	Y	Y	Y	N/A	N/A	N/A	3
Victoria Hospital- Geriatric Rehabilitation Unit/ Victoria hospital/ (2020) (147)							
MB/	Y	Y	Y	N/A	N/A	N/A	3
Rehabilitation Geriatric Services- Seven Oaks General Hospital/ Seven Oaks General Hospital/ (2023) (148)							
MB/	Y	Y	Y	N/A	N/A	N/A	3
Deer Lodge- Geriatric Assessment and Rehabilitation Program/ Deer Lodge Centre/ (2023) (149)							
Quebec							
QC/	Y	Y	Y	N/A	N/A	N/A	3
McGill University Health Centre Transitional care Unit/ McGill University Health Centre/ (2023) (150)							
QC/	Y	Y	Y	N/A	N/A	N/A	3
Jeffery Hale Saint Brigid’s Geriatrics Unit -Functional Rehabilitation Transition Unit/ Jeffery Hale Saint Bridges/ (2023) (151)							
QC/	Y	Y	Y	N/A	N/A	N/A	3
Short Term Stay Sunrise Senior Living Quebec/ Sunrise Senior Living/ (2022)							
*Facilities exist in ON, QC (152)							
New Brunswick							
NB/	Y	Y	Y	N/A	N/A	N/A	3
New Brunswick Extra Mural Program/ Medavie Health Services NB/ (2023) (153)							
NB/	Y	Y	Y	N/A	N/A	N/A	3
Rapid Rehabilitation and Reablement/ Social Supports NB/ (2023) (154)							
NB/	Y	Y	Y	N/A	N/A	N/A	3
Transitional Living Suites / Horizon Health Network NB/ (2023) (155)							
Prince Edward Island							
PEI/	Y	Y	Y	N/A	N/A	N/A	3
Caring for Older Adults in the Community [COACH] Program/ Government of Prince Edward Island/ (2022) (153)							
Nova Scotia							
NS/	Y	Y	Y	N/A	N/A	N/A	3
Victoria General Hospital Transitional Care Unit/ Nova Scotia Health Authority/ (2023) (154)							
Newfoundland and Labrador							
NL/	Y	Y	Y	N/A	N/A	N/A	3
Central Health Restorative Care/ Central Health/ (2023) (155)							
Northwest Territories							
NT/	Y	Y	Y	N/A	N/A	N/A	3
Norman Wells Transitional Care/ Northwest Territories Health Authority/ (2019) (156)							
Yukon							
YT/	Y	Y	Y	N/A	N/A	N/A	3
Bridge-to-Home/ Government of Yukon/ (2023) (157)							

Y, yes; N, no: U, unclear; N/A, not applicable.

JBI critical appraisal checklist for text and opinion.

Q1: Is the source of the opinion clearly identified? Q2: Does the source of opinion have standing in the field of expertise? Q3: Are the interests of the relevant population the central focus of the opinion? Q4: Is the stated position the result of an analytical process, and is there logic in the opinion expressed? Q5: Is there reference to the extant literature? Q6: Is any incongruence with the literature/sources logically defended?

### Data extraction

Textual data were extracted from sources included in the review by 6 independent reviewers (BB, EG, CF, MH, EMc, and SMG) using a modified version of the standardized JBI data extraction tool (S4 Appendix). Two reviewers (BB and EG) pilot-tested the extraction tool with 6 sources, to identify any discrepancies and ensure consistency of data extraction. Extracted data were verified by a second reviewer (BB, EG, EM, MM, and LEW). Conflicts between reviewers regarding data extracted were resolved through discussion or by a third reviewer. Textual data were extracted verbatim from each source, including direct quotes of patient testimonials and transitional care program effectiveness. Unique to this systematic review of text and opinion was the inclusion of multiple sources of webpage links and reports to characterize one transitional care program (S5 Appendix). Textual data were extracted from multiple sources to gather as much information about each transitional care program goal, population or eligibility, setting and length of program, intervention and services offered, and healthcare team members.

### Data synthesis and presentation of results

Findings from textual sources are presented in narrative format to depict characteristics and outcomes of transitional care programs. Textual sources were not graded using the JBI ConQual approach [38] for dependability and credibility of qualitative findings. The ConQual approach is aligned to meta-aggregation in qualitative systematic reviews and does not have formal guidance for use within text and opinion systematic reviews [39]. The omission of a ConQual score is a deviation from the protocol. Textual data are presented in tabular form with a narrative summary to describe how findings address the review’s questions and objectives.

## Results

This 3-phase grey literature search produced a total of 17,092 text and opinion sources, resulting in the identification of 119 transitional care programs in Canada published from 2017 to 2023. Sources include websites, reports, and news articles. We screened 10,117 sources in Zotero where 1,726 sources were sought for retrieval. After screening in Zotero, 1,089 sources were exported to Covidence for full-text review. A total of 271 sources of text and opinion were included in this review (S5 Appendix). See Fig 1. for the PRISMA flow chart.

### Characteristics of Canadian transitional care programs

Model of care was a key characteristic defining the design and delivery of transitional care programs. Three models of care including community, hospital, and facility-based characterize the setting where transitional care programs are delivered in Canada. For example, community-based transitional care programs are delivered within older adults’ home or in temporary apartments. Hospital-based transitional care programs are delivered on designated transitional or rehabilitation units that are designed to support patients’ needs as they prepare to transition out of hospital. Facility-based transitional care programs are delivered in a facility separate from acute care hospitals, such as rehabilitation centers and reactivation centres, or within leased units at long-term care or assisted-living facilities. A total of 42 community, 45 hospital, and 32 facility-based transitional care programs were identified. Differences between provincial/territorial regions including total population and proportion of older adults is important for contextualizing the number of transitional care programs identified. A summary of provincial/territorial population demographics and number of transitional care programs by model of care are presented (Table 2).

**Table 2:** Provincial/territorial transitional care programs in Canada.

Province or Territory	Total Pop 2021*	Pop Age 65+ 2021*	% Age 65+ 2021*	Model of Care			Total
				Community	Hospital	Facility	
Ontario	14,223,942	2,637,710	18.5%	31	30	23	84
British Columbia	5,000,879	1,016,365	20.3%	1	5	4	10
Manitoba	1,342,153	229,050	17.1%	2	5		7
Alberta	4,262,635	629,220	14.8%	4		1	5
Quebec	8,501,833	1,753,530	20.6%		2	1	3
New Brunswick	775,610	177,160	22.8%	2	1		3
Saskatchewan	1,132,505	197,980	17.5%			2	2
Newfoundland and Labrador	510,550	120,610	23.6%		1		1
Nova Scotia	969,383	215,325	22.2%		1		1
Northwest Territories	41,070	4,110	10%			1	1
Prince Edward Island	154,331	32,705	21.2%	1			1
Yukon	40,232	6,050	15%	1			1
Nunavut	36,858	1,605	4.4%				0
Canada	36,991,981	7,021,420	19%	42	45	32	119

Note: No transitional care programs were identified in Nunavut

Source: [40]*

Characteristics defining hospital and facility-based transitional care programs were homogenous however, characteristics of community-based transitional care programs were heterogenous. A summary of characteristics for 119 transitional care programs are presented in Table 3. Transitional care programs are categorized based on province and characterized by program name, program goal, population and eligibility, setting and length of program, intervention and services, and healthcare team members. Additional characteristics of transitional care programs such as funding models, involvement of unpaid caregivers, discharge process, and integration with other health services were not included due to unavailable or unclear data for almost all transitional care programs.

**Table 3:** Results of sources reporting Canadian transitional care programs within community, hospital, and facility-based models of care.

Province/ Program Name/ Author/ (Year)	Model of Care	Program Goal	Population/ Eligibility	Setting/ Length of Program	Intervention/ Services	Healthcare Team Members
Ontario						
ON/ Let’s Go Home (LEGHO)/ One Care Support/ (2022) [41]	Comm	To help older adults and people with health challenges to strengthen their health, independence and quality of life to live at home in a caring community.	Older adults/ In hospital, medically stable, requiring additional supports to transition home.	Client home/ 4–6 weeks	Provides clients access to community services for a short period of time post hospital discharge to ensure a successful recovery period. Care Planner does an assessment and regular check ins, and links clients to various services they may need. Core services provided to all clients include Care Planning, Transportation, Meals on Wheels, and Home Help.	Care Planner
ON/ Richview Community Care Services/ Toronto Central Healthline/ (2022) [42]	Comm	We value our clients’ strengths and strive to support independence. Our services are designed to help clients achieve their maximum potential. Staff work with each client to enable them to complete activities of living.	Older adults (65+)/ In hospital, medically stable, requiring additional supports to transition home.	2-bedroom apartment/ 5–30 day stay	Onsite personal support worker provides 14 hr/day care to assist with activities of daily living, medication administration, safety & reassurance services, meal planning and shopping, walking escorts to on-site areas, laundry.	Personal Support Worker
ON/ Home Concierge—Post-operative care/ Home Concierge/ (2022) [43]	Comm	To ensure older adult patients can transition with ease and comfort back to the place they feel most comfortable–home.	Older adults/ In hospital post-surgical, medically stable, requiring additional supports to transition home.	Client home/ Length not specified	Immediately after surgery, one of our team members can meet you or a loved one at the hospital or medical centre and help them get home safely and set the stage for a speedy recovery. We provide ongoing care support at home for as many days after as needed.	Not specified
ON/ Home at Last/ West Neighbourhood Group/ (2022) [44]	Comm	Coming home from staying in the hospital can be hard if friends or family aren’t there to help. For seniors, it’s much tougher. Our Home at Last program helps by providing a personal support worker to help you get home, and to settle in safely once back home.	Older adults (age 59+) / Lives alone, does not have a caregiver, is being discharged from the hospital.	Supportive apartments / 3–6 months	Personal support workers provide case management, emergency response/ lifeline system, 24-hr personal care, medication monitoring, meal preparation, light housekeeping	Personal Support Worker, Case Manager
ON/ Integrated Comprehensive Care Program/ St. Joseph’s Healthcare Hamilton / (2018) [45]	Comm	To help patients move smoothly from the hospital setting to their home and community. This program provides guidance, care and support during this challenging time and while you recover at home. We strive to improve provider satisfaction, quality and health system outcomes, efficiency of the healthcare system, and patient experience.	Older adults/ In hospital for surgery and chronic diseases including Congestive Heart Failure, Chronic Obstructive Pulmonary Disease.	Client home/ Up to 60 days post hospital discharge	Integrated Care Coordinators meet patients shortly after admission to hospital to plan for your discharge home with services you need to recover. Coordinators work closely with all members of your healthcare team throughout your care journey. A dedicated integrated care coordinator guides patients leaving hospital, dedicated call line is available 24/7 to support patients and families, use of one central electronic health record, provide virtual care in comfort of patients’ home.	Integrated Care Coordinator
ON/ Bayshore @ Home/ Bayshore Healthcare/ (2022) [46]	Comm	To relieve the overburdened hospital sector and allow for more patients to receive the high-quality, personalized care they deserve in the comfort of their homes.	Older adult/ In hospital and in need of short-term support before they can move back home.	Client Home/ 16 weeks	Our team support patients by offering physiotherapy, nursing, and rehab services from home	Care Manager, Nurse Practitioner, Therapists, Personal Support Worker
**Province/ Program Name/ Author/ (Year)**	**Model of Care**	**Program Goal**	**Population/ Eligibility**	**Setting/ Length of Program**	**Intervention/ Services**	**Healthcare Team Members**
ON/ Aghabi Place—Bridges to Care Program/ Able Living/ (2022) [47]	Comm	AbleLiving services provide independent living support solutions for seniors through a variety of in-home services to remain in the community as long as possible.	Older adults/ In hospital	3-bed apartment units/ Up to 90 days	Attendant care services, congregate dining, laundry and community social engagement is included.	Nursing, Therapeutic Services, Home and Community Care Teams
ON/ Southlake@Home/ Southlake Regional Health Centre/ (2020) [48]	Comm	To make clients’ transition home from hospital as easy as possible.	Older adults with frailty or complex needs/ In hospital, requiring additional supports to transition home.	Client home/ Up to 16 weeks	Joint home visits with primary care and community support services. 24/7 phone line for patient and family support. Technology is leveraged to promote communication and self-care (telemonitoring, phone). First day at home visit; check in every day for the first week (then determine how often check in is required), work closely with the hospital to ensure goals are being met post discharge; keep primary care provider up to date on progress; connection with other local community resources (Meals on Wheels, transportation and caregiver support programs).	Coordinator, Nursing, Personal Support Worker, Occupational Therapy, Physiotherapy, Social Worker, Dietician.
ON/ C-Care/ C-Care Health Services/ (2022) [49]	Comm	To ease a transition from hospital to home, C-Care believes in continuous and consistent care. Our staff provide assistance and support in the hospital and come to the home or new residence to provide the same personal, care and encouragement patients and loved ones rely on.	Older adults/ In hospital or at home and requiring additional support post-surgery, after an illness, or decline in health.	Client home/ Length not specified	Assistance with personal care and support (i.e., dressing, feeding, washing, and toileting, as well as advice, encouragement, and emotional and psychological support); 24-hour nursing care, therapy supports. Patient health needs are assessed by clinical consultants and nurses to understand needs and recommend services that best suit patient needs.	Nursing, Speech, Occupational and Physical Therapy, Personal Support Worder
ON/ Comfort Keepers Post Hospital Care/ Comfort Keepers/ (2022) [50]	Comm	By providing uplifting care during recovery at home, we can ease your loved one’s transition after discharge from a hospital or rehabilitation facility. Post-acute care is critical for physical recovery and emotional wellbeing, especially for seniors that are returning home.	Older adults/ In hospital or rehabilitation facility awaiting discharge and requiring additional support to move back home.	Client home/ Length not specified	Comfort Keepers^®^ provides a range of in-home senior care and custom care plans, including following hospital-provided discharge plans and rehab for the elderly after hospital stay, all in the comfort and privacy of home. Caregiver services include transportation, medication reminders, encouragement, companionship, personal care, respite care, patient monitoring and communication. Our caregivers are available 24/7.	Nursing, Personal Support Worker,
ON/ Spectrum Hospital to Home Program/ Spectrum Health Care/ (2022) [51]	Comm	To re-imagine home care for clients and families to provide products and services they need, whenever care is required, delivered by the most qualified and compassionate staff.	Older adults/ In hospital or rehabilitation facility awaiting discharge and requiring additional support to move back home.	Client home/ Length not specified	Our home care services and caregivers customize a care plan that will work with your hospital to home needs. We provide support with personal care, grocery shopping, meal preparation or getting around. Our support staff will be there at the hospital for admittance support, bedside companions, respite care, transportation home. Once you arrive home, we provide visiting nurses, home and personal supports, overnight assistance, live-in caregivers, grocery shopping, transportation, escorts for medical appointments.	Nursing, Caregivers, Personal Support Workers
**Province/ Program Name/ Author/ (Year)**	**Model of Care**	**Program Goal**	**Population/ Eligibility**	**Setting/ Length of Program**	**Intervention/ Services**	**Healthcare Team Members**
ON/ Scarborough Health Network Transitional Care Program [SHN@home]/ Scarborough Health Network/ (2023) [52]	Comm	To extend health care services beyond our hospital walls. Through our SHN@Home program, we create and deliver a seamless discharge care plan for patients who require continued restorative care that follows patients into the community after they have been discharged from one of our hospitals.	Older adults/ In hospital or rehabilitation facility awaiting discharge and requiring additional support to move back home.	Client home/ Up to 16 weeks	Before leaving the hospital, care coordinators meet with patients and families to review discharge plans. Once home, the care team uses different ways to check in and care for you including home visits, phone calls, technology, and coordinate supports with local community resources (i.e. Meals on Wheels, transportation and caregiver support programs). After 8 weeks the care team will review patient progress and plans for ongoing care. After 12 weeks care team connects patient with a Local Health Integration Network (LHIN) Care Coordinator who will conduct an assessment and plan for ongoing care. After 16 weeks care team connects patient with homecare services provided by the LHIN.	Care coordinators, Nurses, Personal Support Workers, Occupational Therapists, Physiotherapists, Social Worker, Dieticians
ON/ SE Care Transitions: Reactivation Care/ Saint Elizabeth Health Care/ (2022) [53]	Comm	To support people to live at home for as long as possible. Our focus is on creating a simple and comfortable process for patients by providing clarity as to the services that will be delivered, ensuring proper education, and reliably delivering on services when requested and as planned.	Older adults/ In hospital but no longer require acute care.	Client home/ Length not specified	Our support beings with monitoring the patient’s continued recovery from their acute care hospitalization, and quickly transitioning to the education and support patients and families need to support self-care and independence. Our reactivation care model provides a personalized monitoring plan. Key Features of our Care Transitions programs include Clinical Expertise and Dedicated Community Based Care Teams; jointly designed and implemented pathways of care; 24/7 access to care; virtual care through our Intelligent Care Platform; Patient-and-Family-Centred Care; Quality and Audit System.	Experienced clinical professionals
ON/ Post Hospital Transition Care/ Global Health Care Services/ (2022) [54]	Comm	To help our clients maximize their health care while maintaining their dignity and personal integrity while respecting cultural and spiritual values.	Older adults/	Client home/ Length not specified	Global Health starts right in the hospital before patients are discharged to provide a personalized care plan; home safety review & modifications; rehab exercises, physical and emotional support; companionship; accompaniment to rehab sessions, doctor appointments and personal events; regular status updates to/from the care team and family. Our care providers assist with: mobility and transferring, bathing, dressing and grooming assistance; errands, grocery shopping, prescription pick-up, housekeeping; meal preparation and feeding assistance; companionship and emotional support; medication reminders; toileting and incontinence care; status reporting to family; safety and fall prevention.	Case manager and caregivers.
ON/ MSH Care@Home/ Eastern York Region North Durham/ (2022) [55]	Comm	To better coordinate the care that you need when you are discharged from Oak Valley Health. Our goal is to make your transition home as stress-free and smooth as possible to help you recover and become more independent.	Older adults/ In hospital but no longer require acute care.	Client home/ Up to 16 weeks	A Transitions Care Lead works closely with all members of your health care team, manage your home care services, connect with family doctors or specialists, and connect with community resources. We provide a care team before you leave the hospital to create a home care plan for the first 72 hours you arrive home. We develop a long-term care plan in collaboration with your caregivers.	Nurse Navigator, Care Coordinator, Primary Care Providers, Occupational Therapist, Social Worker, Chiropodist/ Foot Specialist
**Province/ Program Name/ Author/ (Year)**	**Model of Care**	**Program Goal**	**Population/ Eligibility**	**Setting/ Length of Program**	**Intervention/ Services**	**Healthcare Team Members**
ON/ Care@Home/ Eastern York Region North Durham/ (2022) [56]	Comm	To provide wrap around services at home to individuals with complex needs. We will continue to support you until your goals have been met and your condition is stable.	Older adults/ In hospital but no longer require acute care.	Client home/ Length not specified	Our Care@Home team works closely with all members of your health care team, manage your home care services, connect with family doctors or specialists, and connect with community resources. We provide a care team before you leave the hospital to create a home care plan for the first 72 hours you arrive home. We develop a long-term care plan in collaboration with your caregivers to meet goals. At six weeks your team will assess your goals and add services you may need and an additional 4-weeks of care. We will continue to support your goals and needs, and your plan will be re-evaluated every 4-weeks.	Nurse Navigator, Care Coordinator, Primary Care Providers, Occupational Therapist, Social Worker, Chiropodist/ Foot Specialist
ON/ KHSC@Home/ Kingston Health Sciences Centre/ (2021) [57]	Comm	To ensure clients experience a smooth transition from hospital to home and regain their vitality.	Older adults/ In hospital but no longer require acute care.	Client home/ Up to 16 weeks	The services offered through KHSC@Home to help people regain their vitality may include nursing, personal support for daily-living activities, occupational therapy, physiotherapy, social work, speech therapy and dietitian support. After 16-weeks, if patients continue to have care needs, they are connected with community supports.	Nursing, Occupational Therapy, Physiotherapy, Social Worker, Dietician
ON/ Dynamic Home Care Services/ Kingston Health Sciences Centre/ (2022) [58]	Comm	Our services are customized to empower and maximize independence, provide high-quality elderly care that best match your changing needs.	Older adults / In hospital but no longer require acute care.	Client home/ Length not specified	Our care team works with patients and their caregivers to co-create a customized care plan. Our team provides personal care (e.g., grooming, bathing, dressing), companionship and engaging conversations, meal preparation, cooking, feeding, medication administering or reminders, laundry, light housekeeping, safety supervision, mobility assistance, going for walks, errands, arrangement of and accompaniment to appointments.	Personal Support Workers, Nursing
ON/ PATH: Priority Support to Transition Home/ Timiskaming Home Support/ (2022) [59]	Comm	To provide services that support the well-being of older adults in the District of Timiskaming, enabling them to remain in their home.	Older adults (55 years +) / In hospital, medically stable, requiring additional supports to return home.	Client home/ Length not specified	We provide worry-free patient transitions from hospital to home with services for a timely discharge, able to identify potential safety issues in home, home care assistance with activities of daily living.	Not specified
ON/ Donato House & Post Stroke Transitional Care Program/ ICAN Independence Centre Network/ (2017) [60]	Comm	Independence Centre and Network are passionate about helping seniors live independent, productive and happy lives. We aim to help individuals get back to daily activities and live the fullest life possible.	Older adults/ In hospital, medically stable, requiring additional supports to return home.	2-bedroom home at ICAN property & Client Home/ Length not specified	Your stay at the Donato House includes ICAN’s trained support staff available to assist with personal care needs, homemaking and activities of daily living. Our trained independent living assistants are on-site 24/7 to provide services such as meals, bed linens, towels, cleaning, accommodation and assistance with personal care needs. A lifeline pendant is also supplied. Further, a Stroke Community Navigator will support building a plan for recovery and connect you to agencies. Services are designed to assist in a variety of situations, such as respite/caregiver relief, education, socialization, recreation, assessment and therapy.	Stroke community navigator, Personal Support Worker
**Province/ Program Name/ Author/ (Year)**	**Model of Care**	**Program Goal**	**Population/ Eligibility**	**Setting/ Length of Program**	**Intervention/ Services**	**Healthcare Team Members**
ON/ PHARA Transitional Care Program/ PHARA Independence and Housing/ (2022) [61]	Comm	Our Transition to Home Program assists clients on a short-term basis in transitioning from hospital to home.	Older adults/ In hospital but no longer require acute care.	2-bedroom apartments/ Up to 90 days	Attendants are on site 24/7 to provide assistance with daily living.	Care attendants
ON/ Frail Seniors Transition to Home Program/ West Park Healthcare Centre/ (2022) [62]	Comm	To support seniors, prolong their independent community living, improve their functional abilities, and enhance health and wellness.	Older adults/ In hospital no longer requiring acute care, medically stable and waiting to be discharged home.	Client home/ 4–6 weeks	Our care team works with patients and families to collaboratively develop their goals of care, enabling patients to return home to independent living.	Geriatrician and interprofessional team
					Referrals to community supports and geriatric follow-up as needed.	
ON/ LOFT Specialized Support: Transition from hospital/ LOFT Community Service/ (2022) [63]	Comm	Our programs and services provide safety, stability, and the practical support our clients need to regain their dignity and take control of their lives. We take a Recovery-Based Approach, for each client to set their own goals and feel empowered to make their own choices to change the direction their lives have taken.	Older adults/ In hospital no longer requiring acute care, but unable to be discharged because they have no appropriate caregiver support or appropriate housing.	Client home/ Length not specified	Our intensive, individualized support services enable seniors to thrive as they re-enter the community.	Not specified
ON/ The Key: Post-Hospital Care/ Home Care Assistance/ (2022) [64]	Comm	Post-Hospital Care that promotes a faster recovery for seniors in the comfort of their home.	Older adults/ In hospital, medically stable, and unable to return home without enhanced support.	Client home/ Length not specified	Provides home care tailored to meet the needs of individual patients in post hospital recovery. We provide 24/hr assistance with personal care, medication and appointment reminders, assistance with nutrition and exercise programs, and encouragement, companionship throughout the recovery process.	Caregivers
ON/ One Care: Home at Last/ One Care Home and Community Support Services/ (2022) [65]	Comm	To help transition older adults’ home after a hospital stay or visit to the emergency department.	Older adults 55+ who are frail and/or have complex care needs/ In hospital no longer requiring acute care.	Client home/ Length not specified	Attendant meets patient at hospital, assists with discharge paperwork, gathering belongings from hospital, transportation, pharmacy/grocery store, safety check at home upon arrival, medications education, complimentary meal, stay with patient until they are settled and comfortable at home. Follow-up call from coordinator within 24–48 hours after discharge.	Trained attendant
ON/ Right at Home: Hospital to Home Program (RightTransitions)/ Right at Home Canada/ (2022) [66]	Comm	Right at Home can help seniors and adults with disabilities get home safely and prevent further hospitalizations with regular visits by our trained caregivers. We’re here to make living at home easier, so you can save your energy for recovery.	Older adults/ In hospital no longer requiring acute care.	Client home/ Length not specified	When developing your personalized care plan, we take into account your unique situation when offering services such as: skilled nursing visits, medication reminders, transportation, homemaking, personal care. Communication with family and health care providers to notify of any changes in client condition.	Caregivers
**Province/ Program Name/ Author/ (Year)**	**Model of Care**	**Program Goal**	**Population/ Eligibility**	**Setting/ Length of Program**	**Intervention/ Services**	**Healthcare Team Members**
ON/ Divine Care—Transitional Care Services/ Divine Home Care/ (2022) [67]	Comm	At Divine Home Care we specialize in helping our clients through this transition period until they can fully restore their health and independence.	Older adult/ In hospital no longer requiring acute care.	Client home/ Length not specified	A caregiver meets with clients during their hospital stay, to help the transition home, ensure discharge plans are followed and medications properly administered. We provide anything else necessary to assist in their comfortable recovery including bathing assistance, dressing, transportation, toilet assistance, grocery shopping, meal preparation and clean-up, housekeeping, picking up medications, and personal care.	Not specified
ON/ Canes@Home Transitional Care/ Canes Community Care/ (2020) [68]	Comm	Canes@Home offers a solution to enable patients a safe return home.	Older adults/ In hospital, medically stable, requiring additional supports to transition home.	Client home/ Up to 60 days	The community care coordinator works with patients and families to assess patient care needs, develop a customized care plan with patients and families, and coordinate services before they leave the hospital. Our team follows patient’s progress at home and continues care planning with augmented in-home personal support.	Personal Support Worker, Community Care Coordinator
ON/ Transitional Rapid Access Care Coordination (TRACC)/ Sinai Health/ (2022) [69]	Comm	The Transitional Rapid Access Care Coordination (TRACC) Program provides short-term care coordination for patients recently discharged from the Mount Sinai Hospital Psychiatry Inpatient Unit. The focus of TRACC is to ensure smooth transitions from inpatient hospitalization back to the community.	Older adults/ In hospital, medically stable, requiring additional supports to transition home.	Client home/ Up to 4 weeks post hospital discharge	TRACC uses a recovery-oriented approach to provide recovery plan development, risk assessment and safety planning, symptom management and supportive counselling, medication management, linkage/support access to Primary Healthcare Provider involved in care, initiate referrals for community supports, financial/income support and housing referrals. Programming is offered in a group format and individually to patients for up to 4 weeks post discharge.	Nursing, Occupational Therapists, Social Workers
ON/ Emergency Department Diversion Program/ Grand River Hospital/ (2021) [70]	Comm	To trial a rapid transition of patients through emerge department in order to help them receive more appropriate care from other providers within their community.	Older adults with dementia, post-fall, post-infection, and palliative patients/ Recent hospital admissions or emergency visit	Client home/ Length not specified	After initial care within the ED is completed, the patient is transitioned back to their home with ongoing support and care in the community. Program support patients in their homes through partnerships with other organizations to meet patient needs.	Emerge Nursing, Home Care Coordinators, Personal Support Workers
ON/ Circle of Care/ Sinai Health/ (2022) [71]	Comm	Circle of Care provides community supports that will improve health and independence at home and remove barriers many patients and families come up against on their own.	Older adults with frail and complex needs/ In hospital, medically stable, requiring additional supports to transition home.	Client home/ Up to 90 days	Care Navigators, social workers and clinical teams collaborate with the patient and family on a coordinated care plan to provide: community support services, home care, care navigation, financial aid, caregiver support, rehabilitative services, mental health services, and home-bound programs. Once initiated, the Care Navigator continues to follow patients back at home or in the community, ensuring a smooth transition and adjusting the plan as necessary.	Nursing, Care Navigators, Social Workers, Personal Support Workers
**Province/ Program Name/ Author/ (Year)**	**Model of Care**	**Program Goal**	**Population/ Eligibility**	**Setting/ Length of Program**	**Intervention/ Services**	**Healthcare Team Members**
ON/ Southlake Transitional Care/ Southlake Regional Health Centre/ (2019) [72]	Hosp	To provide specialized, restorative care focused on specific client needs and goals, while working to foster client’s independence and optimize strengths and abilities.	Older adults/ In hospital, medically stable, requiring additional supports to transition home.	Semi-private rooms/ Length not specified	Specialized, restorative care, group exercises.	Nursing, Rehab Assistants, Care Coordinator, Dietician, Physiotherapists, Occupational Therapists, Social Worker, Recreational Therapists, Physician, Pharmacist
ON/ Reactivation Care Unit/ Northeast Specialized Geriatric Centre/ (2023) [73]	Hosp	To provide exceptional and focused care as well as support patients to improve or maintain their independence and functional abilities while in hospital.	Older adults/ In hospital, a decline in health status or functional ability, could benefit from restorative care in a specialized geriatric inpatient unit.	20-bed inpatient unit/ 6–12 weeks	Our care team provides regular assessment, treatment and 24/7 on call coverage with a geriatrician and physician consultation available. Patients are encouraged to mobilize regularly, get dressed daily, and get up out of bed to eat all meals. Discharge planning will include input from patients and families as well as referrals to community supports specific to patient needs.	Nursing, Clinical Manager, Geriatrician, Social Worker, Personal Support Worker, Dietician, Occupational Therapy, Recreational Therapist, Physiotherapist
ON/ St. Joseph’s Continuing Care Centre/ St. Joseph’s Health Centre of Sudbury/ (2019) [74]	Hosp	To work with patients to transition them back to independent living, whether that be home, a retirement residence or an assisted living environment.	Medically complex older adults/ In hospital and requiring rehabilitative, supportive transitional care	64-bed and 72 bed units/ Length not specified	We provide specialized care and services to adults requiring rehabilitative, supportive, or transitional care to maximize their functional potential. Our care teams focus on delivering rehabilitative and restorative care by participating in daily functional activities which are individualized to meet each patient’s unique needs, daily exercise program and use of the therapeutic gym; therapy focusing on restoring independence for basic care needs such as dressing and grooming; use of therapy amenities (i.e., stairs, kitchen, laundry) to enable independence with higher level tasks. Patients will receive customized discharge planning for successful reintegration home.	Nursing, Geriatrician, Dietician, Occupational Therapists, Physiotherapists, Social Worker, Recreation Therapists, Rehabilitation Assistants, Pharmacist
ON/ Acute Care of the Elderly Unit (ACE)/ Michael Garron Hospital/ (2023) [75]	Hosp	To optimize the quality of life for individuals with chronic complex conditions. We provide a senior-friendly environment for patients over 65 with multiple chronic health conditions to help patients maintain their independence, mobility, and function to return home.	Older adults (65+) with multiple chronic health conditions/ In hospital, requiring additional supports to transition home	Michael Garron Hospital Unit/ Length not specified	Support for clients recovering their strength, mobility and maximizing their independence to improve overall health and well-being. Our inter-professional care team works with patients and their families to develop an individualized treatment plan that meets their needs.	Nursing, Personal Support Worker, Transition Navigators, Recreational Therapist, Occupational Therapist, Social Worker, Physicians, Pharmacists
ON/ Short-term Inpatient Rehab/ Michael Garron Hospital/ (2023) [76]	Hosp	To provide short-term, multidisciplinary inpatient rehabilitation to medically stable older adults who will be returning home or to another community setting following an acute care stay at Michael Garron Hospital.	Older adults/ In hospital, medically stable, requiring additional supports to return home	Michael Garron Hospital Unit/ Length not specified	Support for clients recovering their strength, mobility and maximizing their independence to improve overall health and well-being. Our inter-professional care team works with patients and their families to develop an individualized treatment plan that meets their needs.	Nursing, Personal Support Worker, Transition Navigators, Recreational Therapist, Social Worker, Physicians, Pharmacists
**Province/ Program Name/ Author/ (Year)**	**Model of Care**	**Program Goal**	**Population/ Eligibility**	**Setting/ Length of Program**	**Intervention/ Services**	**Healthcare Team Members**
ON/ Centralized Care and Transitions (CCaTT)/ Hamilton Health Sciences/ (2023) [77]	Hosp	To reduce functional and cognitive decline associated with hospital admissions and facilitate timely and safe discharges for frail and high-risk individuals; re-admission avoidance via emergency department; reduce transitions to alternate level of care and prolonged hospital stay by providing early intervention/planning.	Older adults (64+) with frailty/ In hospital, medically stable, high risk for hospital re-admission, requiring additional support to transition home	Hamilton General and Juravinski Hospital Units/ Length not specified	Our care team provides early screening 7 days/week to provide standardized assessment of patients scoring high-risk for frailty. Following Ontario’s Assess & Restore Guidelines we develop and implement care plans to reduce the risk of adverse outcomes; make referrals to appropriate health/social services; and rehabilitative care provided in parallel with acute care.	Nursing, Occupational Therapists, Care Coordinator, Dietician, Physiotherapists, Social Worker, Pharmacist, Clinical Nurse Specialist
ON/ Hospital Outreach Team/ Hamilton Health Sciences/ (2019) [78]	Hosp	To assist older adults in the hospital-to-home transition.	Older adults (64+) with frailty/ In hospital, medically stable, high risk for hospital re-admission, requiring additional support to transition home	Hamilton Health Sciences Hospital Unit/ Length not specified	Our team of healthcare professionals utilize Ontario Ministry of Health Links Model of Care to develop coordinated care plans with patients based on what is most important to the patient. We make referrals to appropriate health and social services and use standardized screening tools to determine root cause for frequent hospital utilization (i.e., unmet needs, undiagnosed cognitive impairment and depression, health literacy issues).	Nursing, Occupational Therapists, Care Coordinator, Dietician, Physiotherapists, Social Worker, Pharmacist, Clinical Nurse Specialist
ON/ Geriatric Rehab Unit (M3)/ Hamilton Health Sciences/ (2023) [79]	Hosp	To provide an assessment and individualized treatment plan in hospital before patients make the transition to return home.	Older adults with complex medical needs/ In hospital, medically stable, high risk for re-admission, requiring additional support to transition home	Juravinski Hospital Unit/ 2–3 weeks	Our care teams meet once a week to discuss your progress and plan for when you will be able to transition home. As part of your rehabilitation patients are expected to do as much of your daily personal care as possible. You will have assistance with activities of daily living and rehabilitation with equipment, transferring in and out of bed and vehicle, using stairs, transportation to appointments, and discharge process home.	Nursing, Occupational Therapists, Care Coordinator, Dietician, Physiotherapists, Social Worker, Pharmacist, Clinical Nurse Specialist
ON/ Ottawa Hospital Rehabilitation Centre (TOHRC)/ The Ottawa Hospital/ (2023) [80]	Hosp	To help patients maximize their independence and re-adapt to rebuilding their lives.	Older adults/ In hospital, requiring support to transition home	Length not specified.	Our team of therapists will work with patients and families to identify what is needed to facilitate your transition to community living. We provide individualized treatment plan, physical rehabilitation, discharge planning, arrangement of community-based services, patient/family teaching, access to a variety of specialized health care providers. Early on during your stay, a discharge plan will be developed to support patients with managing safe community living according to your specific needs.	Nursing, Physiatrists, Social Worker, Therapists, Dietician, Psychologist, Pharmacist, Rehab Therapists, Recreational Therapists, Prosthetics/ Orthotics Team, Chiropodist
ON/ Specialized Seniors Care Inpatient Unit/ Royal Victoria Regional Health Centre/ (2021) [81]	Hosp	To enhance or maintain the health status and quality of life of older adults with complex medical issues.	Older adults (65+) with acute illness/ In hospital, requiring additional support to transition home	34-bed unit at Royal Victoria Regional Health/ Length not specified	Each care provider has a specific role to play to ensure seamless integration of care and together, they strive to provide exceptional care to each patient and family.	Nursing, Physicians, Patient Care Assistants, Behavioral Support Therapists, Geriatrician, Discharge Planner, Social Worker
**Province/ Program Name/ Author/ (Year)**	**Model of Care**	**Program Goal**	**Population/ Eligibility**	**Setting/ Length of Program**	**Intervention/ Services**	**Healthcare Team Members**
ON/ Transitional Care Inpatient Unit/ Royal Victoria Regional Health Centre/ (2021) [82]	Hosp	To provide short-term supports for patients with a firm discharge destination but face a temporary obstacle, such as awaiting home and community care services or slow stream rehabilitation and convalescent care. Focused, short-term supports will enable patients to be more quickly and safely discharged.	Older adults (65+)/ In hospital, requiring additional support to transition home	40-bed unit at Royal Victoria Regional Health/ Length not specified	Our care team provides focused, short-term supports to older adults. Patients are transferred to this unit once a discharge plan is in place. Each care provider has a specific role to play to ensure seamless integration of care and together, they strive to provide exceptional care to each patient and family.	Nursing, Physicians, Patient Care Assistants, Behavioral Support Therapists, Geriatrician, Discharge Planner, Social Worker
ON/ Sudbury Health Sciences COACH/ Health Sciences North/ (2023) [83]	Hosp	To improve functional outcomes of seniors at hospital discharge, reducing the length of stay needed to achieve these outcomes, and resulting in decreased risk for hospital re-admission or awaiting long-term care placement.	Older adults/ In hospital, medically stable, requiring additional supports to transition home	Unit at Health Science North/ Length not specified.	The role of our team is to provide early assessment within 48-hours of hospital admission for adults 65+ and admitted to an inpatient bed. The focus will be on supporting patients that have lost or are at risk to lose independence in their activities of daily living as a result of their acute illness.	Not specified
ON/ West Haldimand Transitional Care Bed Program/ Able Living/ (2022) [84]	Hosp	AbleLiving services provide independent living support solutions for seniors to remain in the community as long as possible.	Older adults/ In hospital, medically stable, no longer require acute care, requiring additional support to transition home	20-bed unit West Haldimand General Hospital/ Length not specified	Our care teams provide short term support for people who are no longer in need of acute hospital care but require additional supports prior to their return home or to another future living arrangement. The program offers 20 transitional beds with enhanced 24/7 personal care support, nursing care, congregate dining, in-house laundry services and therapeutic recreational programming. Specialty nursing and therapeutic services are coordinated by the by the Home and Community Care team of Hamilton Niagara Haldimand Brant Burlington.	Nursing, Personal Support Workers, Laundry Services, Recreational Therapists, Social Worker
ON/ Willett Transitional Care Bed Program/ Able Living/ (2022) [85]	Hosp	AbleLiving services provide independent living support solutions for seniors to remain in the community as long as possible.	Older adults/ In hospital, medically stable, no longer requiring acute care, requiring additional support to transition home	20-beds Willett Urgent Care Centre/ Length not specified	Our care teams provide short term accommodation for people who are no longer in need of acute hospital care but require additional supports prior to their return home, or to another future living arrangement. The program offers 20 transitional beds with enhanced 24/7 personal care support, nursing care, congregate dining, in-house laundry services and therapeutic recreational programming. Specialty nursing and therapy services are coordinated by the Home and Community Care team of Hamilton Niagara Haldimand Brant and Burlington.	Nursing, Personal Support Workers, Laundry Services, Recreational Therapists, Social Worker
ON/ St. Joseph’s Parkwood Hospital Complex Care and Transitional Care Unit/ St. Joseph’s Hospital/ (2022) [86]	Hosp	Restore health, promote independence and maximize patient’s potential to be cared for in their own homes. Complex Care is a part of the health services continuum designed to provide medical management, skilled nursing, and a range of interdisciplinary services to achieve patient identified goals and optimize the quality of life.	Older adults/ In hospital, requiring a more appropriate level of care before transitioning home	Transitional Care Unit St. Joseph’s Hospital/ Length not specified	Our care team restores health, promotes independence and maximizes potential to be cared for in their own homes. The length of time patients spend in Parkwood’s Institute Complex Care Program varies according to patient’s need.	Case manager, Nursing
**Province/ Program Name/ Author/ (Year)**	**Model of Care**	**Program Goal**	**Population/ Eligibility**	**Setting/ Length of Program**	**Intervention/ Services**	**Healthcare Team Members**
ON/ St. Joseph’s Geriatric Rehabilitation Unit/ St Joseph’s Hospital/ (2022) [87]	Hosp	To provide specialized rehabilitation services to help frail patients 60 years and older become healthy, independent, and maintain their quality of life.	Older adults with complex health problems/ In hospital, requiring a more appropriate level of care before transitioning home	30-bed Unit St. Joseph’s Hospital/ 2–6 weeks	The Rehabilitation program helps patients who are struggling to maintain independence after discharged from hospital, following surgery, after an illness, experience declining health. Our care team supports patients with activities of daily living, sitting up in a chair for all meals; attending therapy sessions; participating in walking programs and other exercise groups; and participating in discharge planning and arranging alternative options.	Nursing, Geriatricians, Occupational Therapists, Dietician, Physiotherapists, Social Workers, Pharmacists, Therapeutic Recreation
ON/ Geriatric Engagement and Reintegration Unit/ Ross Memorial Hospital/ (2022) [88]	Hosp	To provide a comprehensive range of services supporting the continuum of care of patients with complex medical needs, restoration/reactivation needs and for successful transition to a community setting.	Older adults/ In hospital, requiring slow stream rehabilitation to return to community setting	11-bed and 22-bed Units at Ross Memorial Hospital/ Length not specified	The rehabilitation program provides low to medium intensity rehabilitation therapy, with interdisciplinary assessments and intervention to adapt to changes related to health conditions and to increase strength and stamina to transition home. This program provides short- or longer-term care for patients requiring skilled nursing or medical care that cannot be met on an ongoing basis in other levels of rehabilitative care or in a long-term care setting.	Not specified
ON/ Ontario Shores Geriatric Transitional Unit/ Ontario Shores Centre for Mental Health Services/ (2021) [89]	Hosp	To provide specialized services to meet the mental health needs of individuals with a primary diagnosis of dementia. This includes psychogeriatric resources to provide assessment, management and transitional care needs of the client with the goal of reintegration to community.	Older adults (55+)/ In hospital, medically stable, primary diagnosis of age-related dementia, requiring support to transition home.	20-bed Unit Ontario Shores Health Centre/ Up to 59 days	Our care team provides comprehensive assessments, person-centred care plans, psychogeriatric resources, family support and education, and connections within the community. All team members provide non-pharmacological interventions specific to patients’ care plan with family involvement. Services includes music therapy, reminiscent therapy, pet therapy, karaoke, balloon badminton, and enhanced discharge planning.	Interprofessional team including Behavioral Therapist
ON/ Halton Complex Transitional Care (CTC)/ Halton Health Care/ (2022) [90]	Hosp	To optimize patients’ quality of life, maximize their independence and improve their function, to the best extent possible.	Older adults/ In hospital, medically stable, requiring additional supports to transition home	3 Units at Halton Health Care (Milton, Georgetown, Oakville/ Trafalgar)/ Length not specified	A team of healthcare professionals will provide short term Complex Medical Management (Medically complex and specialized services) to develop a care plan that enhances quality of life and facilitates discharge to a lighter level of care. During initial weeks of admission, we begin assessments to determine level of function, identify appropriate equipment required, and therapy to help you progress towards your goal. A discharge planner meets with patients early on to discuss returning home.	Nursing, Discharge Planner, Home & Community Care Coordinator, Therapists, Physicians, Recreational therapy, Social Worker, Spiritual Care, Unit Clerk
ON/ Centenary Complex Continuing Care Unit/ Scarborough Health Network/ (2023) [91]	Hosp	To optimize quality of life, support patients with chronic, complex, and multiple non-urgent medical needs with managing their condition, and transition safely from the hospital to home.	Older adults with chronic conditions/ In hospital, medically stable, requiring additional services and resources to transition home	Unit at Centenary Hospital/ Length not specified	We provide short-term services to support patients in improving their abilities and functioning. Care providers support comprehensive geriatric assessment and treatment for elderly patients recovering from a major illness or injury. Complex care for patients with chronic conditions or multi-system disease, such as diabetes, cardiovascular conditions, and stroke.	Specialized Nursing, Physician, Dieticians, Physiotherapists, Occupational Therapy, Social Worker, Personal Support Worker, Pharmacists, Recreational Therapy
**Province/ Program Name/ Author/ (Year)**	**Model of Care**	**Program Goal**	**Population/ Eligibility**	**Setting/ Length of Program**	**Intervention/ Services**	**Healthcare Team Members**
ON/ Day Therapy Transitional Program/ St. Joseph’s Healthcare Hamilton/ (2022) [92]	Hosp	To help patients recently discharged home improve their health and function.	Older adults/ Recently discharged from hospital or working to prevent hospitalization	Outpatient Unit St. Joseph’s Healthcare Hamilton/ Length not specified	The Day Therapy Transitional Program is a time limited program for patients recently discharged from Hospital in which a dedicated team of health care professionals work with patients to improve health and function by working with patients to set goals based and connect with resources in the community to ensure patients maintain improved level of functioning.	Social Worker, Occupational Therapist, Physiotherapist, Recreational Therapist, Dietician, Speech-language Pathologist
ON/ Reactivation Care Centre/ Central LHIN Hospitals Collaborative/ (2022) [93] *Locations at Humber River Health; Trillium Health Partners; Sunnybrook Health Sciences; St Joseph’s Health Centre; William Osler Health System	Hosp	To help patients who no longer need acute care services, but often find themselves waiting for an alternate care facility or community setting like convalescent care. We support patients and families to have access to specialized care they need in a restorative setting.	Older adults/ In hospital, no longer require acute care but need additional support to transition home	Multiple sites / Length not specified	Reactivation care units are newly renovated and provide a quiet, peaceful place for therapy, rehabilitation, and activation supports relative to pace and scale of acute care hospital. Our care teams provide specialized activation therapies in a setting designed to focus on the needs of the patient and support transition to home. Patients enjoy singe or 2-patient rooms with equipment for their needs, and enhanced infection and prevention.	Nursing, Physician, Social Worker, other health professionals
ON/ Ben & Hilda Katz ACE Unit/ Sinai Health/ (2022) [94]	Hosp	Our ACE Medical Unit meets the unique needs of older patients admitted to our hospital to maintain their physical and cognitive abilities, prevent functional decline and promote healthy recovery.	Older adults (65+) with frailty/ In hospital at Mount Sinai, medically stable, additional support to transition home	28-bed Unit Mount Sinai/ Length not specified	Our interprofessional teams on the ACE unit work closely with patients, their families and caregivers to apply early transitional care planning to empower patients to return home and live independently whenever possible.	Skilled Nursing, Interprofessional team
ON/ Bruyere Transitional Care Program/ Bruyere/ (2022) [95]	Hosp	Bruyere provides a bridge for patients to make their way back to the community safely.	Older adults/ In hospital, requiring specialized care before returning home.	21 beds at Élisabeth Bruyère; 120 beds at (49) 60 days	Not specified	Nursing, Rehabilitation Assistant, Physiotherapist, Personal Support Worker
ON/ Hennick Bridgepoint/ Sinai Health/ (2022) [96]	Hosp	We provide remarkable care in the hospital and community through partnership, expert care, practical research and education. We support the care of patients who are ready for discharge from an inpatient stay at Mount Sinai.	Older adults/ In hospital at Mount Sinai or Hennick Bridgepoint, requiring additional support before transitioning to another setting	Transitional Care Unit Hennick Bridgepoint/ Length not specified	The team provides programming to help patients maintain their physical, mental and emotional well-being and therapy to maintain function. For both our transitional care and reconditioning patients, the care teams work with patients and care partners to create a care plan tailored to each patient’s needs and goals. We work with patients and care partners in discharge planning.	Nursing, Patient Care Manager, Physicians, Dieticians, Occupation Therapists, Pharmacy, Physiotherapists, Recreational Therapist, Social Worker
**Province/ Program Name/ Author/ (Year)**	**Model of Care**	**Program Goal**	**Population/ Eligibility**	**Setting/ Length of Program**	**Intervention/ Services**	**Healthcare Team Members**
ON/ St. Michael’s Unity Health ACE Unit/ Unity Health Toronto/ (2022) [97]	Hosp	Unity Health provides comprehensive and compassionate care for elderly people facing age-related issues like chronic disease, mobility problems and more. The ACE unit focuses on patient’s mobility and function to help them maintain their independence and ability to return home.	Older adults (70+)/ In hospital, medically stable, requiring additional support to transition home	8 bed unit at St. Michael’s Hospital/ Length not specified	With physiotherapists and occupational therapists on our team, the unit embraces a collaborative and interprofessional philosophy to providing care. The team works closely with community partners facilitating a safe transition home by ensuring that the appropriate supports are in place.	Nursing, Physiotherapy, Occupational Therapy, Geriatrician
ON/ Seniors and Rehabilitation Day Hospital/ Trillium Health Partners/ (2022) [98]	Hosp	To give patients and families the knowledge and skills to safely manage their health and successfully re-integrate into their community.	Older adults/ In hospital, medically stable, requiring rehabilitation, referral from Credit Valley Hospital	Credit Valley Hospital/ Up to 8 weeks	The program offers Individual assessment and treatment plan to help patients in their recovery. Therapy may include exercise programs, practice of activities of daily living, and education. Progress is evaluated regularly by the team.	Nursing, Occupational Therapists, Physiotherapists, Recreation Therapists
ON/ Assess & Restore Program/ Toronto Grace Health Centre/ (2022) [99]	Hosp	To extend the functional independence of community-dwelling frail seniors and other persons for as long as possible, as well as to reduce caregiver burden by improving psychosocial and health outcomes.	Older adults with frailty/ In hospital,	Assess & Restore Unit at Grace Health Centre/ Length not specified	Our care teams provide sub-acute complex interventions, geriatric rehabilitative interventions, and active recuperative interventions. While patients work towards recovery goals, social workers collaborate with care coordinators to better transition patients back home. Frail seniors and other persons re-entering the community are provided with an appropriate care plan and are closely monitored. Any potential signs of decline will trigger re-admission to the Assess & Restore Unit for additional rehabilitation to maximize their health.	Care Coordinator, Social Worker
ON/ RECOVER Program/ Toronto Grace Health Centre/ (2022) [100]	Hosp	To offer a novel continuum of care pathway for patients after an episode of critical illness. We aim to address healthcare gaps across critical-illness and recovery continuum including coordination of continuity of care from ICU to the community, interprofessional health care teams, and education of patients and families.	Older adults after critical illness/ In hospital, discharge from ICU, medically stable, requiring rehabilitative supports to recover and plan for transition home	RECOVER Unit at Grace Health Centre/ Length not specified	The RECOVER Program promotes care-planning and the appropriate accommodation of patients (including the frail elderly) in inpatient rehabilitation settings after their discharge (<7 days) from ICU. Our care team at RECOVER program coordinate a care plan with ICU team to plan for rehabilitation and transition home. Our care teams provide individual care planning, mental health intervention, mindfulness program, and follow-up with patients in community for up to 1-year.	Nursing, Intensivists, Psychiatrists, Dieticians, Physicians, Pharmacists, Social Worker, Occupational Therapists, Physiotherapists, Recreation Therapists, Respiratory Therapists
ON/ Satellite Health Facility/ St. Joseph’s Health- care Hamilton/ (2022) [101]	Facility	To provide quality care for patients in a safe setting, while supporting the needs of our community throughout the COVID-19 pandemic.	Older adults/ In hospital, no longer require acute care but need additional support to transition home	Satellite Health Facility/ Length not specified	This facility accommodates patients who no longer require hospital care, but who need healthcare support until they transition to another location in the community. As an extension of both hospitals, the Satellite health facility is staffed by an interprofessional team of healthcare providers and primary care physicians.	Nurses, Social Worker, Physician, Home Care Coordinators
**Province/ Program Name/ Author/ (Year)**	**Model of Care**	**Program Goal**	**Population/ Eligibility**	**Setting/ Length of Program**	**Intervention/ Services**	**Healthcare Team Members**
ON/ Restorative Transitional Care/ Providence Care Centre/ (2021) [102]	Facility	Our goal is to ensure patients receive “the right care in the right place,” according to their individual needs. At Providence Transitional Care, it is in a patient’s best interest to return home or a more appropriate care setting.	Older adult/ In hospital, medically stable, requiring rehabilitation and recreation to return home.	2 inpatient units with 64 beds/ Up to 90 days	Everyone who works, learns or volunteers at Providence Transitional Care Centre (PTCC) is working together to provide high quality, safe care. Patients are provided laundry services, on-unit activation rooms, rehabilitative and recreational activities to build strength & stamina.	Recreational Therapists, Physiotherapists, Occupational Therapists, Physicians, Nursing, Allied health.
ON/ Care First Transitional Care Centre/ Care First/ (2022) [103]	Facility	To provide an environment promoting exceptional recovery and also simultaneously enriching the quality of life for individuals who are in need.	Older adults/ In hospital recovering from surgical procedures or medical conditions, no longer requiring acute care.	27-bed unit facility/ Up to 3 months	Our care teams provide nursing and personal care with 24-hr assistance; meals/snacks and dietary consultation; housekeeping; access to onsite primary health and pharmaceutical services; access to community care, support services, social and recreational activities; physiotherapy service and Mon-Fri activation-based activities; access to Social Worker for short and long-term planning; and post-discharge follow-up.	Nursing, Physiotherapy, Primary Care Providers, Recreation Specialists, Social Workers, Dietician
ON/ Integrated Transitional Services Program/ Toronto Grace Health Centre/ (2022) [104]	Facility	To provide patients with a seamless health care pathway from acute care to the community. When all stakeholders are focused on the goal of providing clients with a compassionate and exceptional health care environment, clients have appropriate supports to successfully transition back home.	Older adults/ In hospital and medically stable, no longer requiring acute care and in need of support before community transition.	15-room facility / Length not specified	Our team facilitates recreational and social activities, and also provides co-ordination of community mental health and addiction services.	Nursing, Social Work, Mental Health Case Manager, Chaplain, Personal Support Worker, Unit Clerk
ON/ Pine Villa Transitional Care/ Pine Villa/ (2022) [105]	Facility	To ensure patients and families receive the support they need while preparing for the next step in care to return home.	Older adults/ In hospital, medically stable and requiring additional supports to transition to another long-term living arrangement independently.	68-bed facility/ Short-term, length not specified	Case management coordinates health-related services and supports transitioning to a permanent residence. Assistance with personal care (bathing/ grooming); housekeeping and laundry; daily meals; medication support; social activities and recreational therapy; access to primary care provider. Team expertise from hospital, mental health and addictions, and community support services to improve patient care and well-being.	Personal Support Worker, Nursing, Case Managers, Physician, and Recreation Therapists.
ON/ Villa Pugliese Assisted Living/ Villa Pugliese/ (2022) [106]	Facility	To offer various levels of care as an assisted living alternative to institutionalization (assisted living, short stays, respite/ convalescent care).	Older adults/ In hospital, requiring additional supports to transition home.	10-bed facility/ Short-term, length not specified	Comfort, supervision and assistance with daily tasks, offering transitional care after hospitalization or surgery.	Personal Support Worker, Nursing, Administration staff
**Province/ Program Name/ Author/ (Year)**	**Model of Care**	**Program Goal**	**Population/ Eligibility**	**Setting/ Length of Program**	**Intervention/ Services**	**Healthcare Team Members**
ON/ Integrated Care Solutions/ Bayshore Healthcare/ (2022) [107]	Facility	To help patients transition smoothly back to the community. Bayshore Healthcare is designed to support Ontario Health Teams, across the continuum of care to achieve the quadruple aim (improved patient outcomes, improved patient experience, lower costs of care, improved provider experience).	Older adults/ In hospital no longer requiring acute care, but requiring additional services to return home	Spaces leased from retirement homes/ Length not specified	We have partnered with an Ontario health system to provide 24-hour supports to older adult patients as they transition back to the community. We develop evidence-based care plans and work with patients, their families and their caregivers to help patients remain at home and thriving within their community. Bayshore offers services that foster patient autonomy and quality of life. Our experienced nurses and health care professionals will work with you to design a personalized care plan to help you recover from illness, injury or other medical conditions in the comfort of your home. Working with hospital discharge planner to coordinate home requirements, provide transportation, housekeeping, grocery/prescription pickup, personal support, medication reminders, meal preparation, nursing, 24/7 services.	Nursing, Personal Support Workers, Care Manager, Physiotherapists, Occupational Therapists, Speech and Language Therapy
ON/ Reintegration Care Unit/ Les Centres d’Accueil Héritage (CAH)/ (2022) [108]	Facility	To develop a continuum of care for Francophones to ensure equitable access when and where support is needed.	Francophone (French Speaking) Seniors/ In hospital, no longer require acute care but additional supports to return home.	Two private rooms in Place Saint-Laurent / Length not specified	Case managers conduct a joint assessment with hospital care coordinators allowing eligible seniors to access another level of care until they are able to return home safely. Hospitals can refer French-speaking patients. Services include 24-hr support from francophone team, personal care, meals, assistance in preparing meals, support and navigation of health system.	Francophone personal support workers
ON/ Hillcrest Reactivation Centre/ University Health Network/ (2022) [109]	Facility	Assisting clients as they transition from hospital to community in a comfortable and supportive setting; reduce pressures on acute care and need for long-term care home placement; supporting clients gain their independence; decreasing risk of re-admission to hospital.	Older adults/ In hospital, medically stable, requiring additional supports to transition home.	75-bed facility/ Up to 42 days	We provide group therapy, adaptive exercise, safe sports, expressive arts.	Nursing, Personal Support Worker, Physician, Geriatrician, Recreational Therapist, Physiotherapist, Occupation Therapist, Social Worker
ON/ Binbrook Transitional Care Bed Program/ AbleLiving/ (2022) [110]	Facility	AbleLiving services provide independent living support solutions for seniors through a variety of in-home services to remain in the community as long as possible.	Older Adults/ In hospital, medically stable, requiring additional support to return home.	24-bed facility/ Length not specified	Program offers enhanced 24/7 personal care support, nursing care, congregate dining, in-house laundry services and therapeutic recreational programming. Specialty nursing and therapy services are coordinated by the Home and Community Care team.	Nursing, Therapeutic Services, Personal Support Workers
ON/ Helping Hands Orillia Transitional Bed Program/ Helping Hands/ (2022) [111]	Facility	Helping Hands exists to serve clients in our community by bringing support, maintenance and enhancing their quality of life to live independently. We provide transitional care when patients are unable to go home.	Older adults/ In hospital but no longer require acute care.	12-bed facility/ Up to 90 days	Program provides 24-hour on-site or on-call supervision from a Personal Support Worker, personal care assistance, toileting, transfers and mobility, meals, transportation, friendly visiting and telephone reassurance and much more.	Personal Support Worker
**Province/ Program Name/ Author/ (Year)**	**Model of Care**	**Program Goal**	**Population/ Eligibility**	**Setting/ Length of Program**	**Intervention/ Services**	**Healthcare Team Members**
ON/ Transitional Care Units/ VHA Home Health Care/ (2022) [112]	Facility	To support clients as they move out of the hospital and back into the community.	Older adults/ In hospital, medically stable, requiring additional support to transition home	80-bed Transitional Care Unit/ Length not specified	Our care teams on transitional care units provide multi-disciplinary, high-quality, 24-hr care. Individualized treatment to help the recovery process. Support patients and families as they navigate the transition to their next destination.	Personal Support Workers, Rehabilitation Providers
ON/ Transitional Care Sites/ Canes Community Care/ (2020) [113]	Facility	To help patients who longer require hospital care to receive the care they need in the community.	Older adults with comorbidities and frailty/ In hospital, medically stable, requiring additional support to transition home	Canes facility Central West and Mississauga Halton/ Up to 42 days	Our care team provides planned collaboration between home and community care service providers to ensure a comprehensive and streamlined approach to providing care for complex patients. We provide geriatric assessment; virtual care calls as needed; personal care assistance; meals; programs/activities; medication management; transportation; equipment	Nursing, Personal Support Worker, Occupational Therapy, Physiotherapy, Care Coordinators, Personal Support Workers
ON/ RVH-IOOF Patient Flow Program/ Royal Victoria Regional Health Centre/ (2021) [114]	Facility	To enhance or maintain the health status and quality of life of our patients until they reach their discharge destination.	Older adults (65+)/ In hospital, designated Alternate Level of Care, requiring additional support to transition home	27-bed satellite location/ Length not specified	Our care team provides rehabilitation for patients designated Alternate Level of Care (ALC). Each care provider has a specific role to play to ensure seamless integration of care and together, they strive to provide exceptional care to each patient and family.	Nursing, Physicians, Patient Care Assistants, Behavioral Support Therapists, Geriatrician, Discharge Planner, Social Worker
ON/ Transitional Behavioral Support Unit/ Baycrest/ (2023) [115]	Facility	To provide time-limited, specialized support for seniors whose behaviours have become unmanageable in their current setting, and for whom available resources have been unsuccessful in managing their challenging behaviours.	Older adults with progressive dementia/ In hospital, medically stable, with behaviours that cannot be managed in current environment	23-bed unit in Apotex Centre Facility/ Up to 4 months	Our care teams at Baycrest’s Apotex Centre, Jewish Home for the Aged provides an enhanced care delivery model. In a specialized unit, clients receive stabilization care and then are repatriated back to the originating community.	Interdisciplinary team
ON/ Queen’s Estate Transitional Care Rehab Unit/ Queens Estate/ (2019) [116]	Facility	To provide patients with high quality 24-hr care until they can safely go back home.	Older adults/ In hospital, whose hospital treatment plan is complete but are unable to return to their home right away	Queens Estate LTC facility/ Up to 45 days	Our comprehensive therapeutic services include specialized nursing, wound care, medication administration, catheter care and health monitoring; 24/7 Personal Support Worker services including assistance with bathing, dressing, and companionship services; access to Central LHIN Home and Community Care coordinators providing ongoing discharge planning support; pharmacist services; health coaching towards self-management of chronic conditions; Assistive Devices Program services; falls prevention and exercise program	Nursing, Occupational Therapists, Physiotherapists, Personal Support Worker, Community Care Coordinators, Pharmacists
ON/ Yee Hong Centre for Geriatric Care/ Indus Community Services/ (2018) [117]	Facility	We provide culturally appropriate short-stay transitional beds for Chinese seniors who live in Greater Toronto Area. We focus on reducing the number of Alternate Level of Care patients waiting in hospital and to help seniors transition home.	Chinese older adults/ In hospital, medically stable, requiring additional time and rehabilitation to return home	64-bed unit/ Length not specified	Our care teams provide 24-hour nursing and personal care, access to health professionals, support with activities of daily living, recreation and social programs, medical supplies, and equipment,	Nursing, Personal Support Worker, Intake Coordinator, Dietician, Physiotherapists
**Province/ Program Name/ Author/ (Year)**	**Model of Care**	**Program Goal**	**Population/ Eligibility**	**Setting/ Length of Program**	**Intervention/ Services**	**Healthcare Team Members**
ON/ Sunrise Short Stay Support/ Eastern York Region North Durham Sunrise Senior Living/ (2022) [118] *Facilities exist in QC, ON	Facility	To provide all the support you need to help you recover and transition to the next destination. We aim to preserve dignity, celebrate individuality, enable freedom of choice, encourage independence, and involve family and friends.	Older adults/ In hospital or rehabilitation centre and requiring assistance during an illness or after hospital visit.	Sunrise Retirement Facility/ Up to 30 days	Provides assistance both during an illness or after a hospital visit from Oak Valley Hospital. From homemade meals, engaging in activities, medication management to around-the-clock care, you will experience all of the additional services that aid in a full, speedy recovery.	Skilled Nursing, Personal Support Workers, Therapists
ON/ Perley Health Sub-Acute Care for Frail Elderly [SAFE]/ Perley Health/ (2021) [119]	Facility	To improve the health and well-being of frail elderly patients hospitalized due to surgery, illness, accident to prevent deconditioning that can happen in hospital.	Older adults/ In hospital no longer requiring acute care, but requiring additional services to return home	20-bed unit/ 2–3 weeks	Under SAFE, patients transfer from an acute-care hospital where they can access medical and rehabilitative supports needed to recover fully and return to their place of residence. A Perley Health family physician oversees the care of each transferred patient, with the support of a team of specialists.	Physician, Specialists, Nursing, Personal Support Worker, Physiotherapist, Occupational Therapist, Dietician, Recreation Therapists
ON/ Caledon Transitional Care Program/ Caledon Community Services/ (2022) [120]	Facility	To help clients get back on their feet when an illness or injury has taken its toll.	Older adults (55+)/ In hospital, medically stable, requiring additional support to transition home	3-bed unit/ Up to 30 days	Our care team provides assistance and support with activities of daily living, medication administration, meals, case management, referrals to other health services, rehabilitation plans.	Personal Support Worker, Respite Service Providers, Case Managers, Respite Services Coordinator
ON/ Transitional Care Unit: Windsor Retirement Residence/ Kingston Health Sciences Centre/ (2021) [121]	Facility	To give people the care and support they need to enter a rehab facility or return to a home environment with the best quality of life possible.	Older adults/ In hospital, medically stable, requiring additional supports to transition home	102-bed unit Windsor Retirement Residence/ Average 39 days	Our care team meets patients where they are at, providing short- or medium-term support. We provide access to restorative therapies as well as nursing and personal care support that helps patients for a return to life at home or admission to a rehabilitation facility.	Nursing, Clinical Nurse Specialist, Social Worker, Physician, Recreation Therapy, Physiotherapy
ON/ Rubidge Retirement Residence Transitional Care Unit/ Peterborough Regional Health Centre/ (2020) [122]	Facility	To ensure patients receive the appropriate level of care in an appropriate setting.	Older adult/ In hospital, medically stable, no longer requiring acute care, requiring additional support to transition home	45-bed unit Rubidge Retirement Facility/ Length not specified	Not specified	Nursing, Personal Support Workers, Therapists, Social Worker
**Province/ Program Name/ Author/ (Year)**	**Model of Care**	**Program Goal**	**Population/ Eligibility**	**Setting/ Length of Program**	**Intervention/ Services**	**Healthcare Team Members**
ON/ Rekai Centre Transitional Care Unit/ Sinai Health/ (2022) [123]	Facility	The goal of our healthcare team is to provide support and help patients regain their strength and independence. Our priority is to ensure everyone that leaves our Transitional Care Unit will be stable and settled into an appropriate community setting.	Older adult/ In hospital, medically stable, requiring additional support and specialized medical care to return home	Transitional Care Unit at Rekai Centre/ Up to 60 days	During their stay, our care team will work with patients to help them make informed decisions about their future care needs and living arrangements. We provide specialized care services such as IV antibiotics, support with activities of daily living, wound care, rehabilitation. Many patients might need their homes refurbished for wheelchair accessibility or finding adequate community housing placements. A Social Worker is there to facilitate and organize the placement for the patient and will continue to monitor how they are transitioning in home environments.	Nursing, Social Worker
ON/ Transitional Care Beds/ Niagara Gardens Retirement Manor/ (2022) [124]	Facility	To provide a caring and happy atmosphere while maintaining the privacy and dignity of all our residents.	Older adults/ In hospital, no longer requiring acute care, require temporary accommodation with enhanced care to transition home	10-bed unit Niagara Gardens Retirement Manor/ Length not specified	Our care teams provide 24-hour care, personal care, medication administration, emergency response system, meals, housekeeping, laundry, physiotherapy, medical equipment and supplies. Niagara Gardens provides private and semi-private accommodations in a transitional care unit.	Not specified
British Columbia						
BC/ Personalized Support & Stabilization Team Plus (PSS+) at Robert & Lily Lee Family Community Health Centre/ Vancouver Coastal Health/ (2023) [125]	Comm	To provide rapid access to short-term comprehensive, coordinated and client-centred care in the home for individuals with high priority time-sensitive care needs to improve health outcomes and prevent unnecessary Emergency Department visits and/or hospital admissions.	Older adults at high risk/ In hospital, medically stable, requiring additional supports to transition home.	Client home/ Up to 8 weeks	Team assists clients in prioritizing activities that are important to them and enable them to recover and increase their ability to be independent. Build on individual strengths and goals. Connect clients with ongoing community support services as required.	Nursing, Rehabilitation Specialists, Other Healthcare Clinicians
BC/ Transition Services Team (TST) Vancouver General Hospital/ Vancouver Coastal Health/ (2023) [126]	Hosp	To help senior clients transition from the hospital back into the community and to facilitate access for clients to needed community based services, such as assisted living, residential care and home care team services.	Older adults/ In hospital, medically stable, requiring additional supports to transition from Vancouver General Hospital.	Hospital Unit/ Length not specified	TST acts as a gateway for post-hospital community are by performing centralized intake needs assessments for hospital inpatient adults eligible for and needing publicly-funded facilities and services upon discharge. TST also assists in supporting the reduction of Alternate Level of Care patient days within Vancouver.	Vancouver Coastal Health Care Providers, Pennsylvania Hotel Employees.
BC/ Transition Services at Koerner Pavilion/ Vancouver Coastal Health/ (2023) [127]	Hosp	To help senior clients transition from hospital back to their own community setting.	Older adults/ In hospital, medically stable, requiring addition supports to transition home.	Hospital Unit/ Length not specified	Not specified	Not specified
**Province/ Program Name/ Author/ (Year)**	**Model of Care**	**Program Goal**	**Population/ Eligibility**	**Setting/ Length of Program**	**Intervention/ Services**	**Healthcare Team Members**
BC/ Convalescent Care Program/ Fraser Health/ (2023) [128]	Hosp	To provide short-stay service that helps clients regain their strength and mobility after hospitalization, so they can return back to the community.	Older adults with chronic illness/ In hospital, requiring more time to recover before returning home.	Hospital Unit/ Up to 7 weeks	Staff assist clients to regain their strength and mobility so they can safely carry out their activities of daily living upon returning home. During your stay, your convalescent care team will meet regularly to plan for your needs and progress. We encourage you and your family/caregivers to actively participate in your care and discharge planning. Upon discharge, staff will assist in organizing any necessary equipment for home and community supports.	Nursing, Physiotherapist, Occupational Therapists, Rehabilitation Assistants
BC/ Convalescent Care Program/ Interior Health/ (2023) [129]	Hosp	To provide short-stay service that helps clients regain their strength and mobility after hospitalization, so they can return back to the community.	Older adults with chronic illness/ In hospital, requiring more time to recover before returning home.	Hospital Unit/ 4–8 weeks	Your convalescent care team will meet regularly to plan for your needs and progress. To help you transition back to your community, you will be expected to actively participate in recovery, including goal setting, personal care and mobility. Your care team will work with you to develop a plan to help you achieve your recovery goal.	Nursing, Physiotherapists, Occupational Therapists, Social Worker, Rehabilitation Assistants
BC/ Providence Care Centre/ Providence Care/ (2022) [130]	Hosp	To ensure patients are receiving the right care in the right place, according to their individual care needs	Older adults/ In hospital, medically stable, able to participate in rehab and recreation to return home.	64-bed Hospital Unit/ Length not specified	Our care team provides restorative transitional care, cognitive-behavioural support, and restorative convalescent care. We provide high level of activation, such as mirroring at home routines and support services, such as spiritual care, social work, discharge planning.	Nursing, Home Care Coordinators, Social Workers
BC/ Glengarry Transitional Care Unit/ Island Health/ (2023) [131]	Hosp	To provide comprehensive and interdisciplinary short term transitional care and discharge planning with patients and families in a small and focused unit, to support the timely and safe discharge of patients to appropriate longer-term locations in the community.	Older adults/ In hospital, medically stable, requiring rehabilitation before returning home.	14 bed Hospital Unit/ Length not specified	Glengarry Transitional Care Unit is a regional island-wide resource for people who are affected by combinations of mental health, physical health, behavioural and substance use issues who have multifaceted and unique needs that are often not aligned with established community service delivery models and as such are challenging to place in our current continuum of community-based settings. The length of stay is variable and determined by individual need, with the goal being to transition clients within a relatively short time.	Physician services, Nursing, Community Case Management, Social Worker, Recreational Therapist, Occupational Therapist, Dietician
BC/ Short Term Enablement and Planning Suites (STEPS)/ Island Health/ (2022) [132]	Facility	To allow people to access enhanced health care services in the community within a "virtual ward" concept, thereby reducing the need for hospitalization, offsetting strain on acute care, and creating a better care experience.	Older adults/ In hospital, medically stable, requiring short-term rehabilitation before returning home.	10 Living Suites within LTC facility/ 4–12 weeks	STEPS provide 24-hour support from a team of community health workers who check on clients regularly, providing physical assistance and emotional support. Regular visits from a multidisciplinary team.	Case Manager, Community Physicians, Community Health Workers
BC/ Evergreen House/ Vancouver Coastal Health/ (2023) [133]	Facility	To support older adults transition from hospital back to their own community setting.	Older adults/ In hospital, medically stable, requiring additional supports to transition home.	Designated beds in LTC facility/ Length not specified	Individualized assessment and care planning to meet the physical, psychological, social, spiritual, and environmental needs of residents coping with chronic illness. Services are available to older adults with complex care needs or conditions who cannot live safety and independently at home.	Health Care Team within LTC facility
**Province/ Program Name/ Author/ (Year)**	**Model of Care**	**Program Goal**	**Population/ Eligibility**	**Setting/ Length of Program**	**Intervention/ Services**	**Healthcare Team Members**
BC/ Glenwood Care Centre/ Fraser Health/ (2023) [134]	Facility	To provide social connection, fun activities, exercise, and health checks for seniors who have health challenges and want to keep living as independently as possible.	Older adults/ In hospital, medically stable, requiring additional supports to transition home.	53 bed unit within LTC facility/ Length not specified	Glenwood Care teams provide nursing and personal care; various recreational activities; spiritual care; and physiotherapy through “Moving for Life” Program.	Nursing, Personal Support Workers, Physiotherapists, Health Care Team within LTC facility.
Alberta						
AB/ Alberta Health Services Self-Managed Care Program/ Harmony Caregiving/ (2023) [135]	Comm	To make the hospital to home transition as smooth as possible.	Older adults/ In hospital and requiring additional support to transition home.	Client home/ Length not specified	Personal care services, medication management, toileting & incontinence Care, showering/bathing, transfer/ mobility assistance, meal assistance, catheter care, ostomy care, wound care, appointment accompaniment, grocery shopping, house cleaning, errand running. Care staff meet client at hospital on day of discharge and ensure they are transferred home safely and comfortably.	Nursing, Health Care Aides, Case Manager, Physiotherapy, Occupational Therapist, Massage Therapy
AB/ Post-Hospital Care/ Comfort Keepers/ (2023) [136]	Comm	To ensure our clients retain as much independence as possible while remaining in their familiar and comfortable surroundings.	Older adults/ In hospital, requiring additional support to transition home.	Client home/ Length not specified	Collaborate with hospital to ensure discharge plans and goals are understood. Assistance with medication management, meal preparation, housekeeping, transportation to appointments and running errands.	Personal Care Workers
AB/ Post-Hospital Care/ Care West AB/ (2020) [137]	Comm	To provide customized care to all clients so they can live happier, healthier lives at home.	Older adults/ In hospital, requiring additional support to transition home.	Client home/ Length not specified	Personal care assistance (transfers, bathing, dressing, grooming), meal preparation, medication reminders, housekeeping, laundry, transportation, companionship, and memory care. Care manager available on call 24/7.	Care Manager, Caregiver
AB/ Hope 4 Life/ Hope 4 Life Home Care/ (2017) [138]	Comm	To provide safe hospital discharge care. Our geriatric care services are designed to promote physical and emotional well-being, ensuring care plans evolve to changing needs.	Older adults/ In hospital and requiring additional support to transition home.	Not specified/ Length not specified	Assistance with personal care, activities of daily living, medication administration, companionship and community-oriented activities, meal preparation and assistance with feeding, transportation to appointments and for recreational travel.	Case Management, Occupational Therapy, Physiotherapy, Psychiatry Counselling services, Dietician
AB/ Rehabilitation and Enhanced Community Transition Program/ Home Care Assistance/ (2022) [139]	Facility	To provide residents and clients with quality care in safe, comfortable and supportive environments.	Older adults/ In hospital, requiring additional supports to transition home. Enhanced care for medically complex clients requiring intensive nurse care.	3 Facility Sites / Length not specified	Short stay units provide additional rehabilitation or convalescence for people who, after injury or illness, do not need to be in acute care hospital but who cannot yet manage at home or another care setting. Services provide practice and daily support achieving activities of daily living and walking. Rehabilitation sessions with therapy teams offered up to 2–3 times a week.	Care coordinator, Physician, Nursing, Personal Support Worker, Occupational Therapy, Unit Clerk, Physiotherapy, Social Worker, Dietician, Pharmacists
Saskatchewan						
SK/ Kensington Transitional Beds/ Kensington Gentle Care Home/ (2023) [140]	Facility	To continue caring for clients who no longer need to be cared for in a hospital but who still require healthcare services. Clients can convalesce in transitional beds until they are ready to move back home or another community setting.	Older adults/ In hospital, no longer requiring acute care, require additional support to transition home.	15 Private rooms at Kensington LTC facility/ Length not specified	Saskatchewan Health Authority has partnered with Kensington Gentle Care Home to provide transitional care beds. We provide basic personal care as in a personal care home. At Kensington there are 15 private rooms with bathroom, hospital bed, TV, wi-fi, beautiful garden, heated solarium. Clients have access to a professional health care provider 24/7.	Nursing, therapists, Care Coordinator.
**Province/ Program Name/ Author/ (Year)**	**Model of Care**	**Program Goal**	**Population/ Eligibility**	**Setting/ Length of Program**	**Intervention/ Services**	**Healthcare Team Members**
SK/ Convalescent Care/ William Booth Special Care Home/ (2021) [141]	Facility	The convalescent care program provides additional short-term recuperation support after surgery or serious illness. The focus of this program is centred around activities of daily living and physiotherapy.	Older adults/ In hospital, recovering from surgery or illness, requiring additional support to transition home.	18 Private rooms at William Booth facility/ Up to 6 weeks	Upon admission clients complete My Voice for Sustaining Treatment to develop an advanced care plan. We provide a secure and safe environment with rehabilitation, foot care, hair care, meals, equipment for care, medication administration, nurse call system, personal care, physician services, recreation program	Nursing, Care Coordinator, Physiotherapy, Social Worker, Personal Support Worker, Pharmacists.
Manitoba						
MB/ Priority Home Rapid Response Nursing Team/ Winnipeg Regional Health Authority (2017) [142]	Comm	To provide short-term home care service to help patients understand their chronic disease and support them in managing their health needs independently.	Older adults with chronic disease/ In hospital, requiring additional supports to transition home.	Client home/ 2–3 weeks	Nursing team helps clients understand chronic disease and medications. Call from nurse within 1–2 days of hospital admission to arrange first visit and review discharge plan from hospital. Nurse arranges follow-up; develops health care plan; contacts primary care provider to share this information; arrange community supports; additional in-home or telephone visits as needed.	Nursing
MB/ Priority Home/ Winnipeg Regional Health Authority/ (2017) [143]	Comm	To provide seniors with high needs safe in their homes for as long as possible with community supports.	Older adults/ In hospital, requiring intensive case management and restorative approach to return safely home.	Client home/ Up to 90 days	Program designed to enhance Home Care’s capacity to provide short-term, transitional, intensive case coordination, and restorative services. Case coordinator, and in some cases a therapist (OT, PT), meets client at home within 48-hours of referral. Services include nursing, health coordination, stroke services, home nutrition program, and self & family managed care program. Referral to other community programs/ services.	Home Care Staff, Occupational Therapist, Physiotherapists
MB/ Prairie Mountain Health Transitional Care/ Prairie Mountain Health/ (2023) [144]	Hosp	To provide specialized care to patients who do not require 24/7 medical supervision by a physician but still require some 24/7 nursing care.	Older adults/ In hospital, no longer requiring acute care services.	9 Prairie Mountain Health facilities/ Length not specified	Care teams provide assistance with daily living activities, meals, nursing care, prescription drugs, physiotherapy, occupational therapy, routine laundry and linen services, routine medical and surgical supplies.	Not Specified
MB/ Misericordia Transitional Care Unit (Restorative Care)/ Misericordia Health Centre/ (2023) [145]	Hosp	To provide restorative care serving clients who require complex social and medical support for a limited period of time before transitioning home with community services or to a personal care home, supportive housing or elsewhere.	Older adults/ In hospital, no longer requiring acute-care and not at risk for a rapid decline in medical stability.	3 hospital units, 111 beds / Up to 90 days	Restorative care promotes clients’ ability to adapt and adjust to living as independently and safely as possible. The health-care team focuses on a holistic approach to improve the physical, mental and social functioning of clients. Staff provide care, treatment and services in a way that respects and fosters clients’ dignity and autonomy. During the short-term stay, clients receive on-going treatment while being assessed to determine the best setting for them in the future. Discussions of options take place with both clients and their families.	Nursing, Physician, Health-care Aides, Unit Clerks, Occupational Therapists, Dietitians, Physiotherapists, Social Worker, Pharmacists, and mental health clinicians
MB/ Victoria Hospital- Geriatric Rehabilitation Unit/ Victoria hospital/ (2020) [146]	Hosp	To provide rehabilitation support to clients in the geriatric population who may require an inpatient stay to support their transition back to home or in community.	Older adults (65+)/ In hospital, medically stable and could benefit from rehabilitation support to return home.	30 bed facility/ Length not specified	Daily individual assessment and review of the clinical course and treatment plan for a limited time period, until the condition is stabilized, or the treatment course is completed.	Physicians, Nursing, Personal Support Worker
**Province/ Program Name/ Author/ (Year)**	**Model of Care**	**Program Goal**	**Population/ Eligibility**	**Setting/ Length of Program**	**Intervention/ Services**	**Healthcare Team Members**
MB/ Rehabilitation Geriatric Services- Seven Oaks General Hospital/ Seven Oaks General Hospital/ (2023) [147]	Hosp	To return patients to the highest possible level of function.	Older adults/ In hospital at Seven Oaks and requiring additional support to transition out of acute care.	Seven Oaks Facility/ Length not specified	Care teams provide scheduled therapy, patient and family development of discharge goals, weekly goal setting, assessment of activities of daily living abilities to determine discharge needs.	Physicians, Nursing, Physiotherapists, Occupational Therapists, Social Worker, Personal Support Worker, Dietician, Pharmacist, Rehab Specialists, Geriatric Specialists
MB/ Deer Lodge- Geriatric Assessment and Rehabilitation Program/ Deer Lodge Centre/ (2023) [148]	Hosp	To help patients return safely to the community.	Older adults/ In hospital post-surgery, stroke or following a long stay in a hospital setting.	2 facility units—88 beds/ Length not specified	The team works with you to identify everything that needs to be done to make sure that you have a safe discharge. We review the care plan and your progress often. We will change the estimated date of discharge if you need a shorter or longer length of stay based on your needs and your progress. Community admissions provided based on recommendations from a Geriatric Program Assessment Team.	Not specified
Quebec						
QC/ McGill University Health Centre Transitional care Unit/ McGill University Health Centre/ (2023) [149]	Hosp	To provide continuing supportive care to patients at the Montreal General Hospital and the Glen site who are in the process of transitioning out of the hospital into a new setting in the community.	Older adult/ In hospital, requiring additional supports before transitioning to another community setting	12-bed units at two facility sites/ Length not specified	Not specified	Nursing, Clinical Nurse Specialist, Social Worker, Physician, Recreation Therapy, Physiotherapy, Spiritual Therapy
QC/ Jeffery Hale Saint Brigid’s Geriatrics Unit -Functional Rehabilitation Transition Unit/ Jeffery Hale Saint Bridges/ (2023) [150]	Hosp	To provide seniors with evaluation, treatment and rehabilitation services.	Older adult 65+ with reduced autonomy/ In hospital, requiring additional supports before transitioning to another community setting	16-bed unit/ Up to 50 days	Assessment, treatment, and rehabilitation services for seniors with reduced autonomy. Collaborative goal setting to increase patient’s level of autonomy	Nursing, Social Worker, Care Coordinators, Dietician, Occupation Therapist, Geriatrician, Physician, Nutritionist, Pharmacist, Physiotherapist
QC/ Short Term Stay Sunrise Senior Living Quebec/ Sunrise Senior Living/ (2022) *Facilities exist in ON, QC [151]	Facility	To provide all the support you need to help you recover. We aim to preserve dignity, celebrate individuality, enable freedom of choice, encourage independence, and involve family and friends.	Older adults/ In hospital or rehabilitation centre and requiring assistance during an illness or after hospital visit.	Sunrise Senior Facility/ Up to 30 days	Provides assistance both during an illness or after a hospital visit. From homemade meals, engaging in activities, medication management to around-the-clock care, you will experience all of the additional services that aid in a full, speedy recovery.	Skilled Nursing, Personal Support Workers, Therapists
**Province/ Program Name/ Author/ (Year)**	**Model of Care**	**Program Goal**	**Population/ Eligibility**	**Setting/ Length of Program**	**Intervention/ Services**	**Healthcare Team Members**
New Brunswick						
NB/ New Brunswick Extra Mural Program/ Medavie Health Services NB/ (2023) [152]	Comm	To improve the quality of life of New Brunswickers within their communities. The Extra Mural Program, known as the “hospital without walls,” provides home health services to New Brunswickers in their homes and communities.	Older adults / In hospital no longer requiring acute care and need support to move back home or in another community setting.	Client home/ Length not specified	We help patients get back home early from a health care facility and prevent readmission by providing short-term care at home to people dealing with an acute illness.	Nursing, Social Workers, Respiratory Therapists, Dieticians, Physiotherapists, Occupational Therapists, Speech-Language Pathologists, Rehabilitation Assistants
NB/ Rapid Rehabilitation and Reablement/ Social Supports NB/ (2023) [153]	Comm	To provide individualized services delivered in a quick, seamless, integrated and intensive way to improve better patient outcomes.	Older adults / In hospital no longer requiring acute care with health needs expected to improve with short-term, intensive care to return home.	Client home/ Length not specified	Services provide intensive, short-term care, as well as equipment and supplies, to help restore seniors’ independence so they can remain safely at home following a hospital admission or illness or injury that impaired daily living. Our team helps seniors maximize recovery after illness or injury, learn or relearn the skills necessary to adapt to living independently and to carry out daily activities while living with impairment.	Nursing, Doctors, Home Support Providers, Extra-Mural Program Staff, Social Development Staff, Dieticians, Rehab Assistants
NB/ Transitional Living Suites / Horizon Health Network NB/ (2023) [154]	Hosp	To maximize independence and self-care of people who have challenges.	Older adults / Discharge from hospital and requiring intensive assessment and rehabilitation but do not require intensive nursing care.	20 inpatient bed unit / Length not specified	Transitional living suites provide accessible and self-contained living spaces on site at Stan Cassidy Centre for Rehabilitation. Our team provides a treatment plan developed with patients and caregivers that is directed toward achieving goals of both patient and the team. Services include coordinated care either in-person or by videoconference, education, and individualized services to support you and your family caregiver needs.	Nursing, Dieticians, Occupational Therapy, Physiotherapy, Psychologists, Recreational Therapists, Social Workers, Speech-Language Pathology
Prince Edward Island						
PEI/ Caring for Older Adults in the Community [COACH] Program/ Government of Prince Edward Island/ (2022) [155]	Comm	To improve access to and quality of care for frail older adults and their families and caregivers by supporting patients to remain at home longer and return home from hospital sooner.	Older adults / In hospital no longer requiring acute care and need support to move back home or in another community setting.	Client home/ Length not specified	Direct patient care at home to predict, prevent or proactively manage health crises, and decrease the need for emergency services or admission to hospital. Holistic assessments (including falls risk, home safety screening, and discipline-specific assessments), medication reconciliation, advance care planning and access to community support. In-home CGA by geriatric NP. Tailored interventions for clients. Education and support. Connections with community supports.	Geriatric Nurse Practitioner, Primary Care Provider, Home Care Coordinator.
Nova Scotia						
NS/ Victoria General Hospital Transitional Care Unit/ Nova Scotia Health Authority/ (2023) [156]	Hosp	The 4B Community Transitions Unit cares for people who are waiting to go to a nursing home and do not need to be in a hospital anymore. The care we provide is like that of a nursing home, but it is not a permanent place to live.	Older adult/ In hospital, medically stable, requiring rehab to transition home.	Designated beds within Hospital Unit/ Length not specified	Our care team works with clients and families to deliver personalized care, support rehabilitation, family support with counselling sessions. We plan for your discharge early and connect you with community supports to ease the transition home. Our unit provides meals, rehabilitation, laundry, recreational therapy, foot care, specialized nursing care.	Nursing, Care Team Assistant, Ward Assistant, Physicians, Recreation Therapists, Occupational Therapists, Social Worker, Dietician
**Province/ Program Name/ Author/ (Year)**	**Model of Care**	**Program Goal**	**Population/ Eligibility**	**Setting/ Length of Program**	**Intervention/ Services**	**Healthcare Team Members**
Newfoundland and Labrador						
NL/ Central Health Restorative Care/ Central Health/ (2023) [157]	Hosp	To discharge clients back to their previous living arrangement at their optimal level of functioning. Restorative Care is slow paced, meaning fewer hours of rehabilitation a day to participate in activities of daily living.	Older adult with complex medical needs or frailty/ In hospital, medically stable, requiring restorative care to return home.	5-bed Hospital Unit/ Up to 60 days	The Restorative care program targets individuals in acute care whose recovery takes longer. Our team will assess client’s physical, psychological, emotional, and social health status. Individual group treatment and therapy programs, review client progress to assess effectiveness of treatment and therapy goals, track performance indicators, and provide supportive services for caregivers.	Nursing, Physician, Occupational Therapy, Physiotherapy, Social Workers, Dietician, Pharmacists
Northwest Territories						
NT/ Norman Wells Transitional Care/ Northwest Territories Health Authority/ (2019) [158]	Facility	To support eligible individuals or families who require more assistance than families and community can provide, to allow them to live as independently in their own homes in their own communities as long as possible.	Older adults / In hospital, no longer requiring acute care and need additional supports to transition home.	18-bed facility/ Length not specified	Not specified	Healthcare Aides, Nursing, Activity Coordinator
Yukon						
YT/ Bridge-to-Home/ Government of Yukon/ (2023) [159]	Comm	Bridge-to-Home improves the quality and safety of transitions for patients, care providers by delivering a patient-oriented care transitions bundle. This patient-oriented care transitions bundle equips them with the knowledge and confidence they need to manage their care at home or in the community, especially during transitions.	Older adults / In hospital, no longer requiring acute care and need additional supports to transition home.	Client home/ Length not specified	Bridge-to-Home provides patients, families and essential care partners with the knowledge and confidence they need to manage their care at home or in the community, especially during transitions. Patient-oriented care transitions bundle consists of the Patient Oriented Discharge Summary (PODS), ‘teach-back’ methods for patient and family education, involvement of patients and essential care partners in the discharge process and post-discharge follow-up.	Nursing, Home Care Coordinator

Community transitional care programs provide tailored services, goal-oriented patient-centred care, coordination of care, rehabilitation for older adults, and short-term services ensuring older adults transition safely home. Community transitional care programs provide services to older adults who are medically stable and require additional support to return home. To be eligible for transitional care, older adults are in-hospital and recovering from acute illness, surgical procedure, or complex chronic conditions. Community transitional care programs are delivered within older adults’ home or temporary apartment units. Program length is short-term, between 3 to 4 months, up to a maximum of 6-months. Services include 24/7 clinical care and case management, hospital discharge planning, support with activities of daily living, rehabilitation therapy, meal preparation, housekeeping, transportation to appointments, medication administration, assistive medical devices and equipment, referrals to community supports, and self-management training. Healthcare team members include, but are not limited to, specialized nursing health professionals, care coordinators, rehabilitation therapists (i.e., physiotherapy, occupational, massage), personal support workers, social workers, and dieticians.

Hospital transitional care programs provide services for older adults that are being discharged from hospital, medically stable, and requiring additional support to improve functional and cognitive functioning before returning home. The goal of hospital transitional care programs is to facilitate timely and safe discharges, avoid hospital re-admission, and return older adults to highest level of mental and social functioning through restorative care. Hospital program services provide individualized and specialized 24/7 nursing care to support older adults with activities of daily living, rehabilitation, and routine medical needs. Hospital program length is short-term between 2 to 6 weeks. Healthcare team members include nurses, care coordinators, physiotherapist, occupational therapists, recreational therapists, and dieticians.

Facility-based transitional care programs provide enhanced health services for older adults that no longer need hospital care but require additional support to maintain health status and quality of life before transitioning home. Facility-based programs provide individualized assessment and care planning, 24/7 care for activities of daily living, medication management, recreation and social programs. Designated units with private rooms in long-term care homes or assisted living facilities provide transitional care services between 4 to 12 weeks. Healthcare team members include personal support workers, therapists, and nurses.

#### Reported outcomes

Eighteen transitional care programs reported qualitative and quantitative patient, caregiver, and health system outcomes (Table 4). Outcomes were reported from Ontario (n = 14, Community = 5, Hospital = 5, Facility = 4), British Columbia (n = 2, Hospital = 1 and Facility = 1), New Brunswick (Community = 1), and Prince Edward Island (Community = 1). Patient, caregiver, and health system leader testimonials describe the impact of transitional care programs for improving quality of care, coordinating services, reducing caregiver stress for families, and enhancing rehabilitation and health outcomes for patients. The impact for one older adult patient is described as, “Without the help of my care navigator, I would have ended up in a group home, and I’m not ready for that yet. It would have been extremely difficult to make this happen on my own” [71]. Health system outcomes are reported as quality improvement initiatives for improving patient reported outcomes, coordination of care, and reducing health system costs associated with emergency department visits, hospital readmission, and extended length of stay. The COACH program in Prince Edward Island reports primary care visits decreased by 50% per month, emergency department visits decreased by 33% per month, and $1.41 million (CAD) in health system costs were reduced from 13 clients and families [155].

**Table 4:** Transitional care program reported outcomes.

Province/ Program Name/ Author/ (Year)	Model of Care	Setting/ Length of Program	Transitional Care Program Reported Outcomes
Ontario			
ON/ Integrated Comprehensive Care Program/ St. Joseph’s Healthcare Hamilton/ (2018) [45]	Comm	Client home/ Up to 60 days post hospital discharge	Length of stay in hospital (8.4 before) (6.7 after)
			Percent of patients with Emergency Department visits within 60 days (all cause) (74% before) (61% after)
			Percent of patients with unplanned readmissions within 60 days (all cause) (42% before) (33% after)
			Average length of stay for readmissions (12.2 before) (8.0 after)
ON/ Bayshore @ Home/ Bayshore Healthcare/ (2022) [46]	Comm	Client Home/ 16 weeks	Since 2017, the Bayshore@Home program has saved the health system over 25,000 alternate level of care bed days. The alternate level of care to long-term care program, and launched in November, has saved 11,000 bed days.
			The Bayshore@home transitional care model has also reduced risk of fall by 77% and more than 80% of patients have been diverted from long-term care, with 40% of those patients continuing to live independently in their home.
			"Of the 2,900 patients admitted, 94% said they would recommend the program."
ON/ Southlake@Home/ Southlake Regional Health Centre/ (2019) [48]	Comm	Client home/ Up to 16 weeks	Southlake@Home has saved 5,000 alternate level of care days/year and reduced alternate level of care wait for homecare from avg. 14.2 days to 0 ($2M cost avoidance*)
			Use bed days saved for funded acute purpose and reduce hallway healthcare by 5,000 bed days/year
			Reduce unnecessary emergency department visits ($0.3M/year cost avoidance*)
			“85% of patients strongly agreed/agreed they were provided the care they needed to be supported at home prior to discharge and 86% of patients strongly agreed/agreed that they received the support they needed at home.”
			“100% of homecare providers strongly agreed /agreed they felt they were part of a heath team, 82% of homecare providers strongly agreed/agreed they were satisfied they joined the Southlake@home team.”
ON/ KHSC@Home/ Kingston Health Sciences Centre/ (2021) [57]	Comm	Client home/ Up to 16 weeks	KHSC@Home has helped over 230 people in the first-year transition safely out of hospital by providing 16 weeks of care.
			KHSC has freed up over 6,000 days of hospital beds yearly to care for the sickest patients in our region.
			Average number of days spent in hospital decreased by 56% for patients in the program over a six-month period at the beginning of 2020, and the number of patients needing to visit the emergency department or be readmitted to hospital decreased by 36%. Another important benefit of the program for patients is the better connection between the hospital and primary care. Four per cent of patients did not have a family doctor before entering the program, and through the service were able to get attached to a general practitioner. As well, timely communication between the hospital and primary care means that the majority of patients are able to arrange a visit with their primary care providers within seven days of leaving the hospital.
			Dr. Tim Chaplin, medical director of KHSC’s Emergency Medicine Program, agrees that the transitional care unit does a fantastic job. “It meets patients where they are at, providing short- or medium-term support. We are not staffed or trained to provide that level of care in an emergency department or at an academic tertiary care hospital.” “The people who receive care at the transitional care unit have good outcomes,” says Hogan. “They are more than 50 per cent less likely to be readmitted to hospital within 30 days of leaving the hospital, compared to all other discharged inpatients.”
			"My mental health is better, and my back is a lot better from doing daily physio (five days a week). When I first came here, I couldn’t even tie my shoes. I can dress myself completely now and I couldn’t do that before." “I wholly endorse it, 100 per cent.
			“I go out a couple of times a week with my sisters, the nurses help me with my medication, the food is good–especially the ribs, and you can walk outside on a paved path around the building. The social interaction is nice too. I enjoy what we call the professional bingo league on Tuesdays and Fridays.
			“It’s a good atmosphere and we have fun. “Also, there aren’t as many sick people here as there are in the hospital. People here are close to going home. “You can’t stay here forever but the time that you are here will be very good. I think just about anybody could be happy here.”
**Province/ Program Name/ Author/ (Year)**	**Model of Care**	**Setting/ Length of Program**	**Transitional Care Program Reported Outcomes**
ON/ Circle of Care/ Sinai Health/ (2022) [71]	Comm	Client home/ Up to 90 days	“Without the help of my Care Navigator, I would have ended up in a group home or nursing home, and I’m not ready for that yet,” said one patient who found herself homeless when a miscommunication during a recent hospital stay resulted in losing her apartment. Upon discharge, she moved on a to a six-week transitional care unit before moving into a permanent supportive housing unit for older adults. “It would have been extremely difficult to make this happen on my own. I really needed her and the reassurance that she gave me.”
			Care Navigator at Mount Sinai Hospital, the ability to seamlessly follow up with clinicians on specific challenges or issues their patients might be facing at home, is a significant benefit to the partnership. “Being able to easily connect with the pharmacist to review medications, for example, or closing the loop on a particular patient, really highlights the importance of the continuum of care,” she says.
			Dr. Samir Sinha, Sinai Health System’s Director of Geriatrics says the commitment and expertise Care Navigators bring in helping people remain in the community, and out of hospitals and long-term care homes, is a testament to the value they bring to our already strained health-care system. “In situations where complex social issues or medical conditions are at the heart of what is threatening people to stay healthy and independent in their own homes, Social Work Care Navigators are an integral solution,” he says. “Being able to count on a community expert who can help address social issues–like housing, caregiver burnout, and access to meals, transportation and counselling–is a tremendous asset to the rest of the clinical team.” “Partnering with the Care Navigator alleviates some of the concern the clinical team may feel about their patients managing at home. It’s a relief for them to know there’s a professional set of eyes out there, continuing to assess any risks and being able to communicate to the team if anything changes.”
ON/ Southlake Transitional Care/ Southlake Regional Health Centre/ (2019) [72]	Hosp	Semi-private rooms/ Length not specified	Southlake Transitional Care has helped 300 patients safely transition home in 2019–20, avoiding more than 3,500 ALC days for the hospital. The program continues to raise the bar for integrated care provincially. The percentage of our acute beds used for alternate level of care patients dropped by 25% in 2019–20 compared to the year before.
ON/ Centralized Care and Transitions (CCaTT)/ Hamilton Health Sciences/ (2023) [77]	Hosp	Hamilton General and Juravinski Hospital Units/ Length not specified	Patients seen by CCaTT had lower average lengths of stay compared to similar patients (i.e. “case mix groupings”) that were not seen by the CCaTT in addition to receiving standard hospital care interventions
			There was 27% decrease in alternate level of care days* resulting in 2,515 days decrease in acute alternate level of care and length of stay for individuals waiting for bedded post-acute rehabilitative care.
			CCaTT patients at HHS’ Hamilton General site had 56% lower acute average length of stay and 10% lower post-acute average length of stay compared to Non-CCaTT patients. CCaTT patients at HHS’ Juravinski site had 50% lower acute average length of stay and 8% lower post-acute average length of stay compared to Non-CCaTT patients.
			Total cost avoidance of 3million. (decrease in length of stay + reduced post-acute rehab care + decrease in alternate level of care days—cost of CCaTT human resources = cost savings).
			CCaTT patients had 33% increase in function (Barthel), 89% discharged home, 7% require post-rehabilitative care. CCaTT patients’ pre-post function improved in each of three years with the greatest improvement seen in 2017–18.
ON/ Hospital Outreach Team/ Hamilton Health Sciences/ (2019) [78]	Hosp	Hamilton Health Sciences Hospital Unit/ Length not specified	12 months post-initiation of Care Plan: 40% Fewer Emergency visits; 51% Fewer admissions; 58% Fewer 30-day readmits; 35% Fewer admissions for ambulatory care sensitive conditions.
			• Of 1,013 Patients cared for:
			97% of patients said the team linked them to health services when needed and 88% said their care plan addressed both their health and social needs.
			95% of patients surveyed indicated that they felt as though they have been listened to by their healthcare team; 84% indicated that their healthcare team involved them in making decisions about their care; 89% indicated that their questions and concerns are always addressed; 78% indicated that they leave their healthcare appointments with a clear understanding of what is going to happen next in their care; 81% indicated that their care plan addresses their health and social situation (e.g. housing, nutrition); 73% indicated that their healthcare experience has been improved; 90% indicated that their healthcare team links them to other health services when needed; 94% indicated that they are being helped by the services that they are receiving.
			“Knowing I have someone to call who will call me back helps me feel less anxious. I suffer from depression but have been feeling much better since having someone to help me when I have questions or need things. I get nervous and don’t how to figure these things out on my own.”
			“Thank you for listening to me. I want to keep my mother at home and it is good to talk about how hard it can be sometimes. Thank you for all your help.”
			“You are the only people I have to help me. I have no one else. I now get to all my appointments and when I need anything I know who to call as you always help me. It makes me feel good to have people I trust that check on me and get me the help I need.”
**Province/ Program Name/ Author/ (Year)**	**Model of Care**	**Setting/ Length of Program**	**Transitional Care Program Reported Outcomes**
ON/ St. Joseph’s Parkwood Hospital Complex Care and Transitional Care Unit/ St. Joseph’s Hospital/ (2022) [86]	Hosp	Transition Care Unit St. Joseph’s Hospital/ Length not specified	Over half of the transitional care unit patients we’ve served to date have had their health restored so they feel well enough to go back home, “Previously, when I left the hospital, I felt as if an umbilical cord had been cut,” says one grateful transitional care unit patient. “This time I am sure I can go home.”
ON/ Ben & Hilda Katz ACE Unit/ Sinai Health/ (2022) [94]	Hosp	28-bed Unit Mount Sinai/ Length not specified	Mount Sinai’s ACE strategy has also allowed the hospital to reduce its average total lengths of stay per patient by 28% and decrease its alternate level of care days by 18%. Our older patients are now more likely to go directly home from the hospital instead of nursing homes, and are less likely to be readmitted.
			Patients’ lengths of stay have decreased, and readmissions rates have been cut in half from 15.3% to 7.6%.
			More importantly, ACE Unit patients have a 34 per cent increased likelihood of returning home after their stay in hospital and patient satisfaction rates have risen to as high as 100 per cent.
			The team has been carefully monitoring its patient and system outcomes through independent surveys and measurements. Compared to a year earlier, the use of urinary catheters, and the incidence of pressure ulcers have fallen.
ON/ Restorative Transitional Care/ Providence Care Centre/ (2021) [102]	Facility	2 inpatient units with 64 beds/ Up to 90 days	“Anna had access to a supportive community of peers and a variety of tools to be creative at home. She says having access to similar activities while in the hospital has drastically helped her recovery and healing journey.”
ON/ Care First Transitional Care Centre/ Care First/ (2022) [103]	Facility	27-bed unit facility/ Up to 3 months	Of 466 clients that have used Care First services; 21 clients (4.51%) reported emergency department visits; 24 clients (5.15%) reported hospitalization; 1 client (0.21%) reported hospital readmission within 7 days; 1 client (0.21%) reported hospital readmission within 8–14 days; 5 clients (1.07%) reported falls. Such partnership on transitions of care is translating to savings of at least close to a million dollars to the health care system making it an innovative way to reduce hospital stays and their associated costs.
			93% of the 146 clients surveyed selected "Excellent" or "Very good" to describe their experience at Transitional Care Centre as a service user.
ON/ Helping Hands Orillia Transitional Bed Program/ Helping Hands/ (2022) [111]	Facility	12-bed facility/ Up to 90 days	"“We are so grateful for everything you did for us. You run a top-notch organization and you change our live remarkably, so thank you, thank you.”
			“What a great service, there’s nothing like it. My grandfather is in a transitional care bed, it puts our family’s mind at ease, gives us the time to plan out the next steps. Have done everything possible to get him to his appointments to take that extra off us."
			“I benefitted from the support and kindness of you and your staff. I felt encouraged, safe, and cared for. I was able to rest and regain my strength. I thank you for helping me.”
ON/ Perley Health Sub-Acute Care for Frail Elderly [SAFE]/ Perley Health/ (2021) [119]	Facility	20-bed unit/ 2–3 weeks	From SAFE program there is 18% reduction in the length of hospital stays and SAFE patients were 38% more likely to be discharged home where they were also three times less likely to require home or community care."
			"As compared with a control group who were cared for in hospital until they were ready for discharge, the patients cared for in the Perley Health’s SAFE Unit were also less likely to be readmitted to hospital."
			"By some estimates, SAFE could reduce health care costs by approximately $700,000 per year. "
			"A significant difference between the two groups is that the SAFE patients had more complex disease profiles–more of them suffered from ailments such as coronary heart disease or cancer, for instance. Despite this difference, though, SAFE patients spent less time in hospital, were more likely to return home, and were less likely to require home care or be transferred to long-term care.
			"Patients in this special program had shorter lengths of stay, were more likely to return home and not require additional supports and were no more likely to visit a hospital emergency room than others."
**Province/ Program Name/ Author/ (Year)**	**Model of Care**	**Setting/ Length of Program**	**Transitional Care Program Reported Outcomes**
British Columbia			
BC/ Convalescent Care Program/ Interior Health/ (2023) [129]	Hosp	Hospital Unit/ 4–8 weeks	“While my aunt was in their care that day, the care team had built a relationship of trust, and we were happy to bring her home knowing the team had assessed her carefully and planned for what interventions would be necessary for her to go home.
			“Team-based care (like the kind my aunt received) stresses the importance of including family as valued members of the health-care team, particularly when planning for a person to leave the hospital. Team-based care also focuses on communicating openly with patients and families to balance the caregiver’s needs and expectations with the discharge process offered by the facility.”
BC/ Short Term Enablement and Planning Suites (STEPS)/ Island Health/ (2023) [132]	Facility	10 Living Suites within LTC facility/ 4–12 weeks	Prior to STEPS, patients would have had to wait in hospital if returning home wasn’t an option. However, it is estimated that in 2021, the program saved 2,055 hospital bed-days, which in turn helps to reduce pressure on the healthcare system.
			In a survey carried out by Island Health, 86% of patients and 75% of caregivers rated their experience of Hospital at Home as 10 out of 10.”
			“The staff here are so nice and well trained. They help me with my medication, they support me with the household chores that I can no longer do, and often there is someone to sit with me and keep me company,” says STEPS client Ruby.
			“This program is such a good idea. It’s really needed and it’s working.” Further, STEPS clients not only receive around the clock support, they also have the opportunity to meet other assisted living residents, eat meals together in the dining room, and participate in activities, which is much more conducive to their wellness and ongoing recovery than a hospital setting.
			“STEPS takes a very client-centred approach. We have many elderly clients and families who are coping with a lot and trying to decide whether to make a move to another care setting can be stressful,” says Schafer-Blood.
			“A person’s health is so variable—this program gives our clients an opportunity to stabilize so we can work with them and their families to get them back home with support, or into assisted living or long-term care.”
			“What we’ve heard through our journey is that many times patients say, ‘I have a reason to wake up and put my lipstick on in the morning because the doctor is coming at 10 o’clock’; even though they’re acutely ill, they actually recover faster because they’re at home” (Lead Physician).
New Brunswick			
NB/ New Brunswick Extra Mural Program/ Medavie Health Services NB/ (2023) [152]	Comm	Client home/ Length not specified	Reported data reflect the fiscal year 2022–2023:
			Extra Mural Program has served 26,835 patients; 13,922 patients were admitted, and 13,746 patients were discharged.
			Ratio and number of patient visits to the emergency department decreased from 0.6 to 0.43.
			Patient experience measures indicate 95% of patients rate home health care services positively.
			90% of patients waited 35 days from time of referral for Extra Mural Program–a target goal of 10 days has been established.
			50% of patients waited 4 days between their referral being received and initial care event–a target goal of 1 day has been established.
Prince Edward Island			
PEI/ Caring for Older Adults in the Community and at Home [COACH] Program/ Government of Prince Edward Island/ (2022) [155]	Comm	Client home/ Length not specified	From the COACH pilot program:
			Primary care visits (average appointments per month) decreased by 50%;
			Visits to the emergency department decreased by 33% per month;
			Inpatient admissions decreased by 66% per month;
			Benefits for the system included savings of $1.41 million from 13 clients and families;
			For the COACH clients who had advanced to long-term care (13 clients) and had passed away, the average length of stay in long-term care was 0.65 years, whereas the average lengths of stay in long-term care in Prince Edward Island was 2.6 years;
			Improved staff satisfaction was a major outcome. COACH clients are better able to self-manage and make informed decisions that positively impact their quality of life at home and, when necessary, support smoother transitions to and from acute care or long-term care.

## Discussion

This systematic review of text and opinion characterizes transitional care programs that exist across Canada for older adults to transition from hospital to home. Our grey literature search identified 119 transitional care programs, differentiated by model of care in community (n = 42), hospital (n = 45), and facility-based (n = 32) settings. Most transitional care programs we identified exist in Ontario (n = 84), British Columbia (n = 10), Manitoba (n = 7), and Alberta (n = 5). Ontario has developed the most expansive range of transitional care programs in community (n = 31), hospital (n = 30), and facility-based (n = 23) settings. Of 18 transitional care program reported outcomes, 14 were reported from Ontario and show improved health outcomes and reduced health system costs from fewer emergency visits, hospital readmissions and extended length of stay. Characteristics and reported outcomes of transitional care programs are important for informing health services planning and scaling up of transitional care program models of care across Canada.

Investing in transitional care programs across provincial/territorial regions is particularly important for growing populations of older adults. Canada’s population of older adults is expected to more than double in the next 20 years [2]. An increasing older adult population has many implications in terms of ensuring adequate supports are in place to provide home care services, suitable housing, transportation, and financial resources for rising costs of living. That there are limited examples of transitional care programs in Canada is concerning, particularly for provinces and territories with above average proportions of older adult populations (Table 2). Atlantic provinces (i.e., Nova Scotia, Newfoundland and Labrador, Prince Edward Island), and Quebec have the fewest number of transitional care programs however, experience above average proportions of people aged 65 and older and can anticipate significant increases in the proportion of older adults aged 85 and older over the coming decades [3]. Ontario, British Columbia, Manitoba, and Alberta have five or more transitional care programs, making them better equipped to scale up existing transitional care programs across the province to meet the needs of an aging population. Whereas provinces with few or no transitional care programs require immediate action to plan for implementing transitional care programs.

Transitional care programs provide a means of addressing complex health system challenges facing older adults and contribute towards achieving the quintuple aim of healthcare improvement [160]. The quintuple aim of healthcare aims to address health equity; population health outcomes; clinician well-being; patient experience; and reduce healthcare costs [160, 161]. Reported transitional care program outcomes show improved older adult experience, health outcomes, access to equitable care, clinician and caregiver well-being, and significant reductions in acute care costs associated with emergency visits, extended stays, delayed discharge, and hospital readmission [7, 19, 20]. A systematic review of transitional care programs within long-term care facilities show long-term care transitional care programs improve outcomes with a greater percentage of older adults returning home [29]. These findings are consistent with a scoping review of transitional care programs for trauma patients across the United States [32]. A total of 10 studies describing 9 transitional care programs show readmissions were reduced by 5%, emergency visits reduced by 13%, and follow-up adherence improved by 75% [32].Transitional care programs not only improve quality of care and health outcomes, they also reduce healthcare disparities and inequities attributed to older adults’ lower socioeconomic condition and poor coordination of care during transitions between health settings [162–164]. Interprofessional transitional care teams are well positioned to identify and address inequitable access to healthcare for older adults by assessing individual health needs within community and home settings, such as access to transportation, nutritious food, and resources required to maintain a safe and supportive housing environment. Recognizing the impact of transitional care programs in relation to the quintuple aim for health care [160] could strengthen evidence for investing in transitional care programs as a core health service.

Limited peer reviewed literature exists in Canada identifying transitional care programs. We conducted a grey literature search to fill this knowledge gap, of which the 119 transitional care programs we identified would not have been produced from a search of academic literature. Limited academic literature and large volumes of grey literature makes it challenging for health system decision-makers to find and use best available evidence to respond to urgent and emerging health system crisis, such as the influx of older adults designated with ALC status and long waitlists for long-term care [11]. There is an increasing need to include different sources of evidence, such as grey literature, so that systematic and rapid reviews comprise the most up-to-date information about health system interventions [165]. These findings can inform health system leaders with design and delivery of future transitional care programs, such as tailoring existing transitional care program goals, services, length of program, and healthcare team members. Furthermore, examples of transitional care programs across Canada are particularly important for rapid-cycle improvement and implementation of health services across diverse provincial/territorial governing health authorities. Results from this systematic review are also relevant to other countries with similar nationally funded and regionally governed health care systems (e.g., Australia, New Zealand).

Further research is needed to build the evidence base of *how* transitional care programs are implemented and operationalized in provincial/territorial health systems. For example, interviews with transitional care program leaders and managers would contribute in-depth understanding of lessons learned and challenges and enablers for implementing transitional care programs. This information would benefit health system leaders with tailoring transitional care program design and model of care based on available health system resources (i.e., financial, health human resources, integration with other health services) and urban and rural context. We recommend future research gather more in-depth information about strategies for implementing and funding transitional care programs with health system leaders and transitional care program managers. Furthermore, exploring patient experience and healthcare outcomes would improve the design of patient-centered transitional care programs across different community settings. Most transitional care programs identified in this review were within urban settings, as such we recommend future research investigate model of care and services best suited to meet patient needs within rural communities. Improving uptake of rural transitional care programs is particularly important for addressing health inequities for older adults with limited access to healthcare services, inadequate housing, and risk of extended length of stay in hospital [166].

### Limitations

Findings from this systematic review of text and opinion should be considered in the context of potential methodological limitations. First, our search of grey literature was limited to textual sources and data available online. Due to the high volume of different grey literature sources, we focused on textual sources of grey literature and were unable to include video and audio content. Findings are also limited to data available online by transitional care programs. It was not possible to include additional characteristics of transitional care programs that were of interest such as funding models, program status (i.e., active, in-active, pilot), involvement of unpaid caregivers, discharge process, and integration with other health services due to the fact these data were unavailable or unclear for almost all transitional care programs we identified. Furthermore, due to the high number of transitional care programs identified (n = 119), it was unfeasible to contact each program to verify accuracy and availability of information online. Second, this systematic review of grey literature aimed to characterize the scope of Canadian transitional care programs. Therefore, we did not hand search a comprehensive list of hospitals and long-term care facilities where transitional care programs could exist. It is likely there are additional transitional care programs that were not identified in this review. Lastly, we found many of the questions for assessing methodological quality using the JBI critical appraisal checklist tool [35] were not suitable for the text and opinion sources included in this review (i.e., health centre websites). We recommend that the methodological guidelines for conducting systematic reviews of grey literature be updated to include methodological quality tools relevant to the wide range of text and opinion sources available online.

## Conclusion

A growing body of evidence demonstrates the impact of transitional care programs for improving health and well-being of older adults transitioning from hospital to home. This systematic review maps and characterizes the scope of transitional care programs across Canada, contributing to the evidence base and highlighting provincial/territorial regions requiring greater investment to support increasing populations of older adults. Characterizing transitional care programs is important for informing health services planning and scaling of transitional care programs across Canada. Findings from this systematic review are relevant for other countries aiming to understand the scope of transitional care services and characteristics of these programs that could improve existing health care services for older adult populations. Investments are required to recognize transitional care programs as a core health service that is needed to meet the health care needs of older adults and addressing the quintuple aim of health care. Further research is needed to build an evidence base of processes for operationalizing and implementing transitional care models across different communities specific to the needs of older adult populations, health human resources, and integration with other health services.

## Supporting information

S1 Appendix PRISMA checklist. (DOCX)

S2 Appendix Search strategy. (DOCX)

S3 Appendix Screening tool. (DOCX)

S4 Appendix Data extraction table. (DOCX)

S5 Appendix Program source links. (DOCX)
