# Supplementary material for: Transitional care programs for older adults moving from hospital to home in Canada: A systematic review of text and opinion
Source: PLoS One. 2024 Jul 18;19(7):e0307306. doi: 10.1371/journal.pone.0307306 (PMC11257371; doi:10.1371/journal.pone.0307306)
Supplement: S2 Appendix — (DOCX) [file pone.0307306.s002.docx]

**Appendix B: Search Strategy and Keywords**

Phase 1: Canadian national grey literature resources

1. Government and Legislative Libraries Online Publication Portal (GALLOP)

Search conducted on September 6^th^ 2022, Updated October 14^th^ 2023

http://aplicportal.ola.org/aplicsearch.asp?language=eng

| **Query** | Results | First 10 Pages of Results |
| --- | --- | --- |
| (transitional care) AND (older adult) | 2,254 |  |
| (transitional care) AND (elderly) | 1,802 |  |
| (transitional care) AND (senior populations) | 2,187 |  |
| (transitional care) AND (aged population) | 2,422 |  |
| (transitions care) AND (older adult) | 2,175 |  |
| (transitions care) AND (elderly) | 1,361 |  |
| (senior transitioning care) | 1,858 |  |
| (transitions care for older adults) | 2,249 |  |
| (transitional care) AND (geriatric) | 674 |  |
| (intermediate care programs) AND (older adult) | 1,877 |  |
| (reintegration programs) AND (older adult) | 794 |  |
| (sub-acute care programs) AND (older adult) | 0 |  |
| (post-acute care programs) AND (older adult) | 0 |  |
| (reactivation programs) AND (older adult) | 137 |  |
| (skilled nursing facilities) AND (older adult) | 1,569 |  |
| (short-term transitional care programs) AND (older adult) | 0 |  |

2. Canadian Institute for Health Information (CIHI)

Search conducted on September 15^th^ 2022, Updated October 16^th^ 2023

https://www.cihi.ca/en/access-data-and-reports

| Query | Results |
| --- | --- |
| (transitional care) AND (older adult) | 0 |
| (transitional care) | 36 |
| (senior transitioning) | 20 |
| (senior transitional care) | 3 |
| (transitional care) AND (elderly) | 0 |
| (transitional care) AND (aged population) | 0 |
| (transitions care elderly) | 0 |
| (transitions care for older adults) | 0 |
| (transitional care) AND (geriatric) | 0 |
| (intermediate care programs) AND (older adult) | 0 |
| (reintegration programs) AND (older adult) | 0 |
| (sub-acute care programs) AND (older adult) | 0 |
| (post-acute care programs) AND (older adult) | 0 |
| (reactivation programs) AND (older adult) | 0 |
| (skilled nursing facilities) AND (older adult) | 0 |
| (short-term transitional care programs) AND (older adult) | 0 |

3. Canadian Nurses Association

Search conducted on September 19^th^ 2022, Updated October 19^th^ 2023

https://www.cna-aiic.ca/en

| **Query** | **Results** |
| --- | --- |
| (transitional care) AND (older adult) | 2 |
| (transitional care) | 16 |
| (senior transitioning) | 4 |
| (senior transitional care) | 4 |
| (transitional care) AND (elderly) | 0 |
| (transitional care) AND (aged population) | 2 |
| (transitions care elderly) | 0 |
| (transitions care for older adults) | 2 |
| (transitional care) AND (geriatric) | 0 |
| (intermediate care programs) AND (older adult) | 0 |
| (reintegration programs) AND (older adult) | 0 |
| (sub-acute care programs) AND (older adult) | 2 |
| (post-acute care programs) AND (older adult) | 2 |
| (reactivation programs) AND (older adult) | 0 |
| (skilled nursing facilities) AND (older adult) | 0 |
| (short-term transitional care programs) AND (older adult) | 2 |

4. Theses Canada Portal – Library and Archives Canada

Search conducted on September 26^th^ 2022, Updated October 20^th^ 2023

https://www.bac-lac.gc.ca/eng/services/theses/Pages/search.aspx

| **Query** | **Results** |
| --- | --- |
| transitional care AND older adult | 44 |
| (transition care for older adult) | 56 |
| (transitional care) AND (senior population) | 34 |
| (seniors transitioning care) | 62 |
| (transitional care) AND (elderly) | 44 |
| (transitional care) AND (aged population) | 43 |
| (transitions care elderly) | 51 |
| (transitions care for older adults) | 67 |
| (transitional care) AND (geriatric) | 25 |
| (intermediate care programs) AND (older adult) | 15 |
| (reintegration programs) AND (older adult) | 1 |
| (sub-acute care programs) AND (older adult) | 13 |
| (post-acute care programs) AND (older adult) | 2 |
| (reactivation programs) AND (older adult) | 12 |
| (skilled nursing facilities) AND (older adult) | 189 |
| (short-term transitional care programs) AND (older adult) | 0 |

5. Google Programmable Search Engine for Canadian Federal Documents

Search conducted on September 28^th^ 2022, Updated October 23^rd^ 2023

https://cse.google.com/cse?cx=007843865286850066037%3A3ajwn2jlweq#gsc.tab=0

| **Query** | **Results** | First 10 Pages of Results |
| --- | --- | --- |
| (transitional care) AND (older adult) | 140,000,000 |  |
| (senior transitional care) | 24,700,000 |  |
| (transitional care) AND (elderly) | 7,840,000 |  |
| (transitions care) AND (older adults) | 144,000,000 |  |
| (transitional care) AND (aged population) | 222,000,000 |  |
| (transitions care elderly) | 61,300,000 |  |
| (transitions care for older adult) | 148,000,000 |  |
| (transitional care) AND (geriatric) | 26,800,000 |  |
| (intermediate care programs) AND (older adult) | 175,000,000 |  |
| (reintegration programs) AND (older adult) | 4,720,000 |  |
| (sub-acute care programs) AND (older adult) | 225,000,000 |  |
| (post-acute care programs) AND (older adult) | 7,300,000,000 |  |
| (reactivation programs) AND (older adult) | 8 |  |
| (skilled nursing facilities) AND (older adult) | 4,230,000,000 |  |
| (short-term transitional care programs) AND (older adult) | 37,200,000 |  |

6. Canadian Agency for Drug and Technologies in Health (CADTH)

Search conducted on October 4^th^, 2022, Updated November 1^st^ 2023

https://www.cadth.ca/

| **Query** | **Results** |
| --- | --- |
| (transitional care) AND (older adult) | 339 |
| (transitional care) | 523 |
| (transitional care) AND (senior population) | 78 |
| (transitional care) AND (elderly) | 152 |
| (transitional care) AND (aged population) | 452 |
| (senior transitional care) | 82 |
| (transitions care) AND (older adults) | 339 |
| (transitions care elderly) | 152 |
| (transitions care for older adults) | 339 |
| (transitional care) AND (geriatric) | 53 |
| (intermediate care programs) AND (older adult) | 133 |
| (reintegration programs) AND (older adult) | 3 |
| (sub-acute care programs) AND (older adult) | 2 |
| (post-acute care programs) AND (older adult) | 14 |
| (reactivation programs) AND (older adult) | 144 |
| (skilled nursing facilities) AND (older adult) | 54 |
| (short-term transitional care programs) AND (older adult) | 194 |

Phase 2: Canadian advanced Google search and news media sources

| **1. Advanced Google Search - Canada**  **Search Conducted July 26^th^ 2022, updated search October 4^th^ 2023** Limits: language: English, published after 2016  https://www.google.ca/advanced_search | **Results** |
| --- | --- |
| **Query** | |
| “transitional care” AND “older adult” | First 10 pages of results (Approx. 100) |
| “transitional care” AND “elderly” |  |
| “transitional care” AND “senior populations” |  |
| “transitional care” AND “aged population” |  |
| “transitions care” AND “older adult” |  |
| “transitions care” AND “elderly” |  |
| “senior transitioning care” |  |
| “transitions care for older adults” |  |
| “transitional care” AND “geriatric” |  |
| “intermediate care programs” AND “older adult” |  |
| “reintegration programs” AND “older adult” |  |
| “sub-acute care programs” AND “older adult” |  |
| “post-acute care programs” AND “older adult” |  |
| “reactivation programs” AND “older adult” |  |
| “skilled nursing facilities” AND “older adult” |  |
| “short-term transitional care programs” AND “older adult” |  |
|  |  |
| **2. ProQuest News and Newspaper**  **Search Conducted on August 10^th^ 2022, updated search October 5^th^ 2023** Limits: language: English, published after 2016, location by country (Canada) https://www.proquest.com/news/advanced?accountid=10406&parentSessionId=XRWQ6SykBI5QNkfqAoPS80LMTIdMim7PjyctqFD1JHQ%3D | **Results** |
| **Query** | |
| (transitional care) AND (older adult) AND loc(Canada) | 115 |
| “transitional care” AND loc(Canada) | 319 |
| “senior transitioning” AND loc(Canada) | 0 |
| (senior transitional care) AND loc(Canada) | 702 |
| (transitional care) AND elderly AND loc(Canada) | 146 |
| (transitional care) AND (aged population) AND loc(Canada) | 56 |
| (transitions care for older adults) AND loc(Canada) | 1,070 |
| (transitional care) AND geriatric AND loc(Canada) | 22 |
| (intermediate care programs) AND (older adult) AND loc(Canada) | 564 |
| (reintegration programs) AND (older adult) AND loc(Canada) | 18 |
| (sub-acute care programs) AND (older adult) AND loc(Canada) | 5 |
| (post-acute care programs) AND (older adult) AND loc(Canada) | 7 |
| (reactivation programs) AND (older adult) AND loc(Canada) | 39 |
| (skilled nursing facilities ) AND (older adult) AND loc(Canada) | 317 |
| (short-term transitional care programs) AND (older adult) AND loc(Canada) | 20 |
|  |  |
| **3. Nexis Uni   Search Conducted on August 24^th^, 2022, updated search October 6^th^ 2023** Limits: language: English, published after 2016, location by country (Canada) https://advance-lexis-com.ezproxy.library.dal.ca/BISAcademicResearchHome/?identityprofileid=B9WJ2958411&crid=e0e8c06b-65e2-4713-a2ef-a9f0b1cd8c9e | **Results** |
| **Query** | |
| transitional care program older adult | 30 |
| transitional care | 1,031 |
| senior transitioning | 2,602 |
| senior transitional care | 50 |
| transitional care elderly | 540 |
| transitional care aged population | 676 |
| transitions care elderly | 90 |
| transitions care for older adults | 13 |
| transitional care AND geriatric | 75 |
| intermediate care programs AND older adult | 0 |
| reintegration programs AND older adult | 3 |
| sub-acute care programs AND older adult | 0 |
| post-acute care programs AND older adult | 0 |
| reactivation programs AND older adult | 0 |
| skilled nursing facilities AND older adult | 36 |
| short-term transitional care programs AND older adult | 0 |
|  |  |

Phase 3: Provincial-territorial Advanced Google Search

| **Suffix** | Google searches were conducted between September 6^th^ to October 20^th^ 2022 | **Results** |
| --- | --- | --- |
| British Columbia,  Alberta, Saskatchewan, Manitoba,  Ontario,  Quebec,  New Brunswick, Nova Scotia,  Prince Edward Island, Newfoundland and Labrador, Nunavut,  Yukon,  Northwest Territories | **Query** | |
|  | “transitional care” AND “older adult” | First 10 pages of results (Approx. 100) |
|  | “transitional care” AND “elderly” |  |
|  | “transitional care” AND “senior populations” |  |
|  | “transitional care” AND “aged population” |  |
|  | “transitions care” AND “older adult” |  |
|  | “transitions care” AND “elderly” |  |
|  | “senior transitioning care” |  |
|  | “transitions care for older adults” |  |
|  | “transitional care” AND “geriatric” |  |
|  | “intermediate care programs” AND “older adult” |  |
|  | “reintegration programs” AND “older adult” |  |
|  | “sub-acute care programs” AND “older adult” |  |
|  | “post-acute care programs” AND “older adult” |  |
|  | “reactivation programs” AND “older adult” |  |
|  | “skilled nursing facilities” AND “older adult” |  |
|  | “short-term transitional care programs” AND “older adult” |  |
|  |  |  |
