# Supplementary material for: Transitional care programs for older adults moving from hospital to home in Canada: A systematic review of text and opinion
Source: PLoS One. 2024 Jul 18;19(7):e0307306. doi: 10.1371/journal.pone.0307306 (PMC11257371; doi:10.1371/journal.pone.0307306)
Supplement: S3 Appendix — (DOCX) [file pone.0307306.s003.docx]

Appendix D: Grey Literature Screening Tool

Transitional Care Programs in Canada for Older Adults Transitioning from Hospital to Home: A Systematic Review of Text and Opinion

**Review Question:**

What transitional care programs exist across Canada for older adults to support moving from hospital to home; and what are the characteristics and outcomes of these programs?

**For each item, check the following criteria. If any criteria are not known, include.**

Article author and year: _________________________________________________________________

|  | Tick if excluded in section |
| --- | --- |
| **Preliminary Questions** |  |
| Is this publication from 2016 or later?  *If yes, move to next question.*  *If no, stop screening and exclude as “Wrong publication date”.* | □ |
| Is this publication Canadian?  *If yes, move to next question.*  *If no, stop screening and exclude as “Wrong country of publication”/* | □ |
| **Section 1: Participants** |  |
| Does the publication concern older adults (65 years of age and older) and/or Indigenous adults 55 years of age and older?  *If yes, move to the next question.*  *If no, stop screening and exclude as “Wrong patient population”.* | □ |
| **Section 2: Phenomena of Interest** |  |
| Is the study focused on transitional care programs that support older adults moving from hospital to home?  *If yes, move to the next question.*  *If no, stop screening and exclude as “Does not concern care program of interest”.* | □ |
| If the study is focused on interventions designed for older adults waiting in hospital, emergency departments, observation stays in hospital, or to be transitioned to long-term care placement? (Commonly referred to as transitional care units or alternative level of care unit)  *If yes, stop screening and exclude as “Does not concern care program of interest”.*  *If no, move to the next question.*  Note: This review will consider the characteristics of transitional care programs across Canada. Characteristics include components of the program of care such as, specific services or interventions offered, types of care settings, characteristics of the healthcare teams, characteristics of patient populations, integration with other healthcare services, resources and services offered to unpaid caregivers, and impact of the program.  Note: There are interchangeable terms for transitional care in the literature. These include: Intermediate care programs, short-term transitional care programs, reintegration programs, sub-acute care, post-acute care, reactivation programs, and skilled nursing facilities. | □ |
| **Section 3: Context (See Preliminary Questions)** |  |
| **Section 4: Types of Publications** |  |
| Is the publication: a report, policy literature, working papers, a media source, an expert report, a website, a government document, a white paper, an internal document, an evaluation, a policy, or a procedure?  *If yes, move to the next question.*  *If no, stop screening and exclude as “Wrong publication type”.* | □ |
