# Supplementary material for: Transitional care programs for older adults moving from hospital to home in Canada: A systematic review of text and opinion
Source: PLoS One. 2024 Jul 18;19(7):e0307306. doi: 10.1371/journal.pone.0307306 (PMC11257371; doi:10.1371/journal.pone.0307306)
Supplement: S4 Appendix — (DOCX) [file pone.0307306.s004.docx]

Appendix D: Data Extraction Instrument

| **Information to be Extracted** | **Description** | |
| --- | --- | --- |
| Date of Search: |  | |
| Region: (Location) |  | |
| Source & URL: (Type of Text) |  | |
| Year of Publication: |  | |
| Author(s): |  | |
| Language: |  | |
| Search Terms: |  | |
| Name of Program/Service: |  | |
| Type of Program Delivery:  (Goal of program, mission/vision, assessment, care planning, treatment) |  | |
| Services Offered: |  | |
| Care Setting: |  | |
| Characteristics of Healthcare Team: |  | |
| Characteristics of Patient Population: |  | |
| Integration with other health services: |  | |
| Engagement/ Services for unpaid caregivers: |  | |
| **Qualitative Findings:** | **Theme, pattern, concepts, categories** | **Illustrative quote** |
| **Outcomes:**  **Patient** (i.e., functional status, quality of life) **Family/caregiver** (caregiver burnout, caregiving responsibilities, financial burden) **Health System** (i.e., length of hospital stay, health services utilization, health service costs) |  |  |
| **Quantitative Findings:** | **Narrative description of findings** | **Findings (intervention group and control group, or before and after, if applicable)** |
| **Outcomes:**  **Patient** (i.e., functional status, quality of life) **Family/caregiver** (caregiver burnout, caregiving responsibilities, financial burden) **Health System** (i.e., length of hospital stay, health services utilization, health service costs) |  |  |
