# Supplementary material for: Transitional care programs for older adults moving from hospital to home in Canada: A systematic review of text and opinion
Source: PLoS One. 2024 Jul 18;19(7):e0307306. doi: 10.1371/journal.pone.0307306 (PMC11257371; doi:10.1371/journal.pone.0307306)
Supplement: S5 Appendix — (DOCX) [file pone.0307306.s005.docx]

Appendix E: Complete list of grey literature sources for Canadian transitional care program models of care

| **Province/**  **Program Name/ Author/  (Year)** | **Model of Care** | **Source Links** |
| --- | --- | --- |
| ON/  Let's Go Home (LEGHO)/  One Care Support/ (2022) (42) | Comm | <https://www.onecaresupport.ca/lets-go-home-legho/>   - <https://www.onecaresupport.ca/about-us/hear-from-our-clients/> - https://www.onecaresupport.ca/about-us/mission/ |
| ON/  Richview Community Care Services/  Toronto Central Healthline/ (2022) (43) | Comm | <https://www.richviewccs.ca>   - <https://www.caregiverexchange.ca/Services/Display/60827/Short_Stay_Transitional_Beds?site=TCO> - <https://www.torontocentralhealthline.ca/displayService.aspx?id=60827> |
| ON/  Home Concierge - Post-operative care/  Home Concierge/ (2022) (44) | Comm | <https://www.thehomeconcierge.ca/blog/what-is-convalescent-care-and-how-can-you-do-it-from-your-own-home>   - <https://www.thehomeconcierge.ca/post-operative-care> - <https://www.thehomeconcierge.ca> |
| ON/  Home at Last/  West Neighbourhood Group/ (2022) (45) | Comm | <https://www.westnh.org/hal/>   - <https://tngcommunityto.org/Programs-Services/Programs/Home-at-Last?cat=4146> - <https://sites.google.com/site/torontocentralhal/> |
| ON/  Integrated Comprehensive Care Program/  St. Joseph’s Healthcare Hamilton / (2018) (46) | Comm | <https://www.stjoes.ca/hospital-services/integrated-comprehensive-care-icc-> |
| ON/  Bayshore @ Home/  Bayshore Healthcare/ (2022) (47) | Comm | <https://www.bayshore.ca/2023/03/27/bayshores-innovative-home-programs-improve-alc-patient-outcomes-increase-system-capacity/>   - <https://www.bayshore.ca/services/home-care/medical-services/> |
| ON/  Aghabi Place - Bridges to Care Program/ Able Living/ (2022) (48) | Comm | https://ableliving.org/programs/#transitional-wellness |
| ON/  Southlake@Home/  Southlake Regional Health Centre/ (2020) (49) | Comm | <https://southlake.ca/wp-content/uploads/2020/03/Southlake@Home-Guide-2020.pdf>   - <https://southlake.ca/southlakehome/> - <https://www.leadershiftproject.ca/uploads/1/1/8/4/118409684/leadershift_conference_slide_deck.pdf> |
| ON/  C-Care/ C-Care Health Services/ (2022) (50) | Comm | <https://www.c-care.ca/services/post-surgical-transportation/>   - <https://www.c-care.ca/services/post-surgery/> - <https://www.c-care.ca/services/post-surgical-transportation/> - <https://www.c-care.ca/blog/home-care/transition-hospital-homecare/> |
| ON/  Comfort Keepers Post Hospital Care/  Comfort Keepers/ (2022) (51) | Comm | <https://www.comfortkeepers.ca/mississauganorth/care-services/transition-services/>   - <https://www.comfortkeepers.ca/transition-services/> - <https://www.comfortkeepers.ca/mississauganorth/care-services/in-home-elder-care-services/> - <https://www.comfortkeepers.ca/post-hospital-care/> |
| ON/  Spectrum Hospital to Home Program/ Spectrum Health Care/ (2022) (52) | Comm | <https://spectrumhealthcare.com/services/senior-care-services/>   - https://spectrumhealthcare.com/services/senior-care-services/ |
| **Province/**  **Program Name/ Author/  (Year)** | **Model of Care** | **Source Links** |
| ON/  Scarborough Health Network Transitional Care Program [SHN@home]/ Scarborough Health Network/ (2023) (53) | Comm | <https://www.shn.ca/areas-of-care/seniors-health/shnhome/>   - <https://www.shn.ca/seniors-health/> - <https://www.shn.ca/areas-of-care/seniors-health/complex-continuing-care/> |
| ON/  SE Care Transitions: Reactivation Care/ Saint Elizabeth Health Care/ (2022) (54) | Comm | <https://acutecare.sehc.com/services-solutions?gclid=EAIaIQobChMIpv-5w_i6_AIVGI3ICh1jbgm-EAAYASAAEgK7AvD_BwE>   - <https://acutecare.sehc.com/information> |
| ON/  Post Hospital Transition Care/  Global Health Care Services/ (2022)  (55) | Comm | <https://healthcareservicesottawa.ca/ressources/faqs/>   - <https://healthcareservicesottawa.ca/home-care/post-hospital-care%E2%80%A9/transition-care/> - <https://healthcareservicesottawa.ca/ressources/faqs/> |
| ON/  MSH Care@Home/  Eastern York Region North Durham/ (2022) (56) | Comm | <https://static1.squarespace.com/static/5ef00d2f7bc5bf6a756dbe09/t/6138de11b2b9065a0b13d08e/1631116820461/21-7267+MSH+Care%40Home+Brochure.pdf> |
| ON/  Care@Home/  Eastern York Region North Durham/ (2022) (57) | Comm | <https://static1.squarespace.com/static/5ef00d2f7bc5bf6a756dbe09/t/62057c641f188654ef29a99e/1644526692897/21-7267+Care%40Home+Plus+Brochure+v4--.pdf> |
| ON/  KHSC@Home/  Kingston Health Sciences Centre/ (2021) (58) | Comm | <https://www.proquest.com/docview/2229976996?accountid=10406&parentSessionId=BIfgkNrkdcLIvd%2BpWTOvUPsDAvJgAUaSbA%2FKzL7wul4%3D>   - <https://kingstonhsc.ca/khscconnect/news/transitional-care-unit-grows-again> - <https://kingstonhsc.ca/khscconnect/news/standing-tall-transitional-care-unit> |
| ON/  Dynamic Home Care Services/  Kingston Health Sciences Centre/ (2022) (59) | Comm | <https://www.dynamichomecareservices.com/team>   - <https://www.dynamichomecareservices.com/team> |
| ON/  PATH: Priority Support to Transition Home/ Timiskaming Home Support/ (2022) (60) | Comm | <https://homesupportservices.ca/path-priority-support-to-transition-home/> |
| ON/  Donato House & Post Stroke Transitional Care Program/  ICAN Independence Centre Network/ (2017) (61) | Comm | <https://www.ican-cerd.com/perch/resources/icanprograms-classesbrochureen.pdf>   - <https://www.ican-cerd.com/post-stroke.php#Transitional-Care-Program> - <https://www.ican-cerd.com/perch/resources/icanpoststroketransitionen.pdf> |
| ON/  PHARA Transitional Care Program/ PHARA Independence and Housing/ (2022) (62) | Comm | <https://static1.squarespace.com/static/5a7bac64edaed821286c5b5f/t/62fdaf08cfa6414bd9593187/1660792594589/2022+-+04+-+12+-+PHARA+Annual+Report+-+v3+%28reading+layout+-+final+1%29.pdf>   - [https://www.phara.org/services/#transition](https://www.phara.org/services/#transition ) |
| **Province/**  **Program Name/ Author/  (Year)** | **Model of Care** | **Source Links** |
| ON/  Frail Seniors Transition to Home Program/  West Park Healthcare Centre/ (2022) (63) | Comm | <https://www.westpark.org/en/Services/FrailSeniors>   - <https://www.westpark.org/PatientsAndFamilies/ReferringtoWestPark1> - <https://www.westpark.org/AboutUs/WhoWeAre> |
| ON/  LOFT Specialized Support: Transition from hospital/ LOFT Community Service/ (2022) (64) | Comm | <https://www.loftcs.org/services/>   - <https://www.loftcs.org/services/one-on-one-services/> |
| ON/  The Key: Post-Hospital Care/ Home Care Assistance/ (2022) (65) | Comm | <https://thekey.ca/our-services> |
| ON/  One Care: Home at Last/  One Care Home and Community Support Services/ (2022) (66) | Comm | <https://www.onecaresupport.ca/services/helping-you-at-home/home-at-last/> |
| ON/  Right at Home: Hospital to Home Program (RightTransitions)/ Right at Home Canada/ (2022) (67) | Comm | <https://www.rightathomecanada.com/barrie/services/specialty-care/hospital-to-home>   - <https://www.rightathomecanada.com/barrie/about-us> - <https://www.rightathomecanada.com/barrie/services> - <https://www.rightathomecanada.com/resources> |
| ON/  Divine Care - Transitional Care Services/  Divine Home Care/ (2022) (68) | Comm | <https://www.divinehomecare.ca/information-centre>   - <https://www.divinehomecare.ca/services> - <https://www.divinehomecare.ca/information-centre> |
| ON/  Canes@Home Transitional Care/  Canes Community Care/ (2020) (69) | Comm | <https://www.canes.on.ca/services/caneshome-transitional-care>   - https://www.canes.on.ca/files/documents/services/CANESHome_Patient_InfoSheet.pdf |
| ON/  Transitional Rapid Access Care Coordination (TRACC)/  Sinai Health/ (2022) (70) | Comm | <https://www.mountsinai.on.ca/care/psych/outpatient-clinics-and-services> |
| ON/  Emergency Department Diversion Program/ Grand River Hospital/ (2021) (71) | Comm | https://www.grhosp.on.ca/assets/documents/ACTION_Summer2021.pdf |
| ON/  Circle of Care/  Sinai Health/ (2022) (72) | Comm | <https://www.circleofcare.com/improving-patient-outcomes-with-a-warm-community-hand-off/> |
| ON/  Southlake Transitional Care/ Southlake Regional Health Centre/ (2019) (73) | Hosp | <https://southlake.ca/wp-content/uploads/2019/08/SL2422_10-Restorative-Care-Unit-Handbook.pdf>   - <https://southlake.ca/news/ontario-expanding-home-and-community-care-in-york-region-investments-in-frontline-services-key-to-ontarios-plan-to-end-hallway-health-care/> |
| **Province/**  **Program Name/ Author/  (Year)** | **Model of Care** | **Source Links** |
| ON/  Reactivation Care Unit/  Northeast Specialized Geriatric Centre/ (2023) (74) | Hosp | https://www.nesgc.ca/Clinical-Services/Reactivation-Care-Unit   - <https://www.nesgc.ca/Research-Evaluation/Transitional-Care-Beds-in-Response-to-COVID-19>   https://www.nesgc.ca/Clinical-Services |
| ON/  St. Joseph's Continuing Care Centre/  St. Joseph’s Health Centre of Sudbury/ (2019) (75) | Hosp | <http://www.sjsudbury.com/index.php/st-josephs-continuing-care-centres-clarion-site-celebrates-its-1-year-anniversary/>   - https://www.thesudburystar.com/news/local-news/st-josephs-to-open-new-facility-on-lasalle-boulevard#:~:text=Joseph's%20has%20announced%20expansion%20plans,CEO%20Kari%20Gervais%20said%20Tuesday |
| ON/  Acute Care of the Elderly Unit (ACE)/  Michael Garron Hospital/ (2023) (76) | Hosp | https://www.tehn.ca/programs-services/complex-continuing-care |
| ON/  Short-term Inpatient Rehab/  Michael Garron Hospital/ (2023) (77) | Hosp | https://torontocentral.rehabcareontario.ca/Services/Display/190605/Term_Inpatient_Rehabilitation_Program |
| ON/  Centralized Care and Transitions (CCaTT)/  Hamilton Health Sciences/  (2023) (78) | Hosp | https://hnhb.behaviouralsupportsontario.ca/Uploads/ContentDocuments/HHS%20CPHS%20-%20Integrated%20Senior's%20Table%2031%20Oct.pdf |
| ON/  Hospital Outreach Team/  Hamilton Health Sciences/  (2019) (79) | Hosp | https://www.cfn-nce.ca/wp-content/uploads/2019/01/OHalloran-Kelly-Hospital-Without-Walls-Presentation.pdf |
| ON/  Geriatric Rehab Unit (M3)/  Hamilton Health Sciences/  (2023) (80) | Hosp | <https://www.hamiltonhealthsciences.ca/areas-of-care/seniors-care/geriatric-rehab-unit/> |
| ON/  Ottawa Hospital Rehabilitation Centre (TOHRC)/ The Ottawa Hospital/  (2023) (81) | Hosp | https://www.ottawahospital.on.ca/en/clinical-services/deptpgrmcs/departments/rehabilitation-centre/ |
| ON/  Specialized Seniors Care Inpatient Unit/ Royal Victoria Regional Health Centre/ (2021) (82) | Hosp | https://www.rvh.on.ca/areas-of-care/specialized-seniors-care-inpatient/ |
| ON/  Transitional Care Inpatient Unit/  Royal Victoria Regional Health Centre/ (2021) (83) | Hosp | https://www.rvh.on.ca/areas-of-care/transitional-care-inpatient-unit-tcu/ |
| ON/  Sudbury Health Sciences COACH/ Health Sciences North/ (2023) (84) | Hosp | <https://hsnsudbury.ca/en/Services-and-Specialties/Medicine>   - https://www.northeasthealthline.ca/displayservice.aspx?id=158844 |
| **Province/**  **Program Name/ Author/  (Year)** | **Model of Care** | **Source Links** |
| ON/  West Haldimand Transitional Care Bed Program/  Able Living/  (2022) (85) | Hosp | <https://ableliving.org/programs/#transitional-wellness>   - <https://www.hnhbhealthline.ca/displayService.aspx?id=196595> |
| ON/  Willett Transitional Care Bed Program/ Able Living/  (2022) (86) | Hosp | <https://ableliving.org/programs/#transitional-wellness>   - https://www.hnhbhealthline.ca/displayService.aspx?id=196595 |
| ON/  St. Joseph's Parkwood Hospital Complex Care and Transitional Care Unit/  St. Joseph’s Hospital/ (2022) (87) | Hosp | https://www.sjhc.london.on.ca/areas-of-care/complex-care   - https://hospitalnews.com/transitional-care-unit-restoring-health/ |
| ON/  St. Joseph’s Geriatric Rehabilitation Unit/ St Joseph’s Hospital/  (2022) (88) | Hosp | <https://www.sjhc.london.on.ca/areas-of-care/specialized-geriatric-services/specialized-geriatric-services-inpatient>   - https://www.sjhc.london.on.ca/media/1937/download |
| ON/  Geriatric Engagement and Reintegration Unit/  Ross Memorial Hospital/ (2022) (89) | Hosp | <https://rmh.org/programs-and-services/continuing-care-program>   - https://www.rmh.org/document/services-profile |
| ON/  Ontario Shores Geriatric Transitional Unit/  Ontario Shores Centre for Mental Health Services/ (2021) (90) | Hosp | https://www.ontarioshores.ca/services/geriatric-transitional-unit-gtu   - https://www.ontarioshores.ca/what-expect/seniors-treatment-and-recovery |
| ON/  Halton Complex Transitional Care (CTC)/  Halton Health Care/ (2022) (91) | Hosp | <https://www.mississaugahaltonhealthline.ca/pdfs/Halton%20Healthcare%20-%202018%20Patient%20Guide.pdf>   - <https://www.haltonhealthcare.on.ca/services_/28763/t28377-complex-transitional-care-department--ctc> - <https://mississaugahalton.rehabcareontario.ca/Services/Display/179037/Complex_Transitional_Care_Department> - <https://www.haltonhealthcare.on.ca/site_files/content/services/pdf/complex-transitional-care-department-ctc/ctc---otmh-info-sheet.pdf> - <https://www.haltonhealthcare.on.ca/site_files/content/services/pdf/complex-transitional-care-department-ctc/ctc---gh-info-sheet.pdf> - <https://www.haltonhealthcare.on.ca/site_files/content/services/pdf/complex-transitional-care-department-ctc/ctc---mdh-info-sheet.pdf> |
| ON/  Centenary Complex Continuing Care Unit/  Scarborough Health Network/ (2023) (92) | Hosp | <https://www.shn.ca/areas-of-care/seniors-health/complex-continuing-care/> |
| ON/  Day Therapy Transitional Program/  St. Joseph’s Healthcare Hamilton/ (2022) (93) | Hosp | https://www.stjoes.ca/hospital-services/support-services-and-specialty-disciplines/day-therapy-programs/transitional-program |
| **Province/**  **Program Name/ Author/  (Year)** | **Model of Care** | **Source Links** |
| ON/  Reactivation Care Centre/  Central LHIN Hospitals Collaborative/ (2022) (94)  *Locations at Humber River Health; Trillium Health Partners; Sunnybrook Health Sciences; St Joseph’s Health Centre; William Osler Health System | Hosp | <https://www.ppno.ca/wp-content/uploads/2021/08/Reactivation-Care-Centre-A-Central-LHIN-Hospitals-Collaborative-09-28-17.pdf>   - <https://www.reactivationcarecentre.ca/church> - <https://www.hrh.ca/2018/04/16/reactivation-care-centre-offers-a-bridge-for-patients/> - <https://www.williamoslerhs.ca/en/areas-of-care/seniors-care.aspx> - <https://www.williamoslerhs.ca/en/areas-of-care/reactivation-care.aspx> - <https://www.reactivationcarecentre.ca/finch> - <https://www.toronto.com/life/wellness/alc-patients-known-as-bed-blockers-straining-scarborough-hospitals/article_9d28f365-f6af-5bae-bd36-e1bf7fe7fe55.html> - https://quorum.hqontario.ca/Portals/0/Users/223/67/12767/Hospital_QIP_Overcrowding_PartII_Webinar.31July2019-%20PDF.pdf?ver=2019-08-16-142403-113 |
| ON/  Ben & Hilda Katz ACE Unit/  Sinai Health/ (2022) (95) | Hosp | <https://sinaigeriatrics.ca/services/mount-sinai-2/the-ben-and-hilda-katz-ace-medical-unit/>   - <https://hospitalnews.com/rethinking-traditional-hospital-model-elder-care/> - http://support.supportsinai.com/site/PageServer?pagename=mainsite_yourimpact_sinaistories_triumpofhealthyaging |
| ON/  Bruyere Transitional Care Program/ Bruyere/ (2022) (96) | Hosp | https://www.greystoneretirement.ca/transitional-care-at-greystone-village-retirement/   - Transitional care trend cited as one more way elderly being shortchanged by health system. *Sudbury Star.* (March 18, 2021 Thursday). https://advance-lexis-com.ezproxy.library.dal.ca/api/document?collection=news&id=urn:contentItem:627J-4S91-JC56-J0GW-00000-00&context=1516831. - <https://www.ontariohealthcoalition.ca/index.php/harsh-light-on-problem-groups-see-worrying-trend-of-seniors-pushed-into-poor-care/> - <https://www.bruyere.org/en/transitional-care> - https://www.bruyere.org/en/blog/health-system-capacity-greystone?ly=4 |
| ON/  Hennick Bridgepoint/  Sinai Health/ (2022) (97) | Hosp | <https://www.hennickbridgepointhospital.ca/en/patients-and-visitors/transitional-care.asp>   - https://www.sinaihealth.ca/news/supporting-patients-during-one-of-lifes-biggest-transitions/ |
| ON/  St. Michael's Unity Health ACE Unit/ Unity Health Toronto/ (2022) (98) | Hosp | https://unityhealth.to/areas-of-care/programs-and-clinics/seniors-care/#st-michaels-hospital-geriatric-emergency-management-gem-2   - <https://unityhealth.to/2017/03/cart-full-of-fun-helps-seniors-ace-their-hospital-stay/> - <https://www.smhdom.com/divisions/general-internal-medicine/ctu-specifics/acute-care-of-the-elderly-unit-and-palliative-care-unit> |
| ON/  Seniors and Rehabilitation Day Hospital/ Trillium Health Partners/ (2022) (99) | Hosp | https://www.thp.ca/patientservices/seniors/Pages/Seniors-and-Rehabilitation-Day-Hospital.aspx |
| ON/  Assess & Restore Program/ Toronto Grace Health Centre/ (2022) (100) | Hosp | https://www.torontograce.org/programs-services/slow-pace-rehabilitation/assess-and-restore-program/ |
| ON/  RECOVER Program/ Toronto Grace Health Centre/ (2022) (101) | Hosp | https://www.torontograce.org/programs-services/slow-pace-rehabilitation/the-recover-program/ |
| ON/  Satellite Health Facility/ St. Joseph’s Health- care Hamilton/ (2022) (102) | Facility | https://www.stjoes.ca/coronavirus/satellite-health-facility |
| **Province/**  **Program Name/ Author/  (Year)** | **Model of Care** | **Source Links** |
| ON/  Restorative Transitional Care/  Providence Care Centre/ (2021) (103) | Facility | <https://providencecare.ca/wp-content/uploads/2021/09/PTCC-Provider-Guide-2021.pdf>   - <https://providencecare.ca/providence-transitional-care-centre/> - <https://providencecare.ca/providence-transitional-care-centre/inpatient-care-services/> - <https://providencecare.ca/services-referrals/inpatient-transitional-care/> - <https://providencecare.ca/providence-transitional-care-centre/inpatient-care-services/discharge-leaving/> - <https://providencecare.ca/providence-care-transitional-care-centre-changes-the-face-of-health-care/> - <https://www.oha.com/news/providence-transitional-care-centre-now-open-and-admitting-patients> - <https://www.thewhig.com/news/local-news/transitional-care-centre-to-open-at-former-st-marys-of-the-lake-hospital-site> |
| ON/  Care First Transitional Care Centre/  Care First/ (2022) (104) | Facility | <https://carefirstontario.ca/wp-content/uploads/2018/10/2017-2018_Annual_Report_En.pdf>   - <https://carefirstontario.ca/wp-content/uploads/2022/05/Carefirst_Transitional_Care_Centre_Brochure_12.04.2022.pdf> - <https://carefirstontario.ca/services/transitional-care/> - <https://carefirstontario.ca/services/home-care/> - <https://carefirstontario.ca/wp-content/uploads/2017/02/5.3-Carefirst-Transtional-Care-Overview-Sep-2018.pdf> |
| ON/  Integrated Transitional Services Program/ Toronto Grace Health Centre/ (2022) (105) | Facility | <https://www.torontograce.org/programs-services/integrated-transitional-services/> |
| ON/  Pine Villa Transitional Care/ Pine Villa/ (2022) (106) | Facility | <http://pinevilla.ca/>   - <https://www.sprintseniorcare.org/programs-and-services/pine-villa-transitional-care/> - <https://www.torontocentralhealthline.ca/displayservice.aspx?id=120629> - <https://northtorontooht.ca/wp-content/uploads/2019/10/NT-OHT-Full-Submission-191009.pdf> - <https://www.caregiverexchange.ca/Services/Display/120629/Transitional_Care_Site?site=TCO> |
| ON/  Villa Pugliese Assisted Living/ Villa Pugliese/ (2022) (107) | Facility | <https://www.villapugliese.com/>   - <https://www.seniorcareaccess.com/home/villa-pugliese-assisted-living-facility> - <https://www.ask4care.com/healthcare-links/> |
| ON/  Integrated Care Solutions/  Bayshore Healthcare/ (2022) (108) | Facility | <https://www.bayshore.ca/services/government/integrated-care-solutions/>   - <https://www.bayshore.ca/services/home-care/medical-services/therapy-services/> - <https://www.bayshore.ca/services/home-care/medical-services/post-surgery-hospitalization/?gclid=CjwKCAjw0ZiiBhBKEiwA4PT9zwLClp8GQLJjqMiO61CUXaEMubHcmCrAe4TDFoyZJP7Pvw0ayKJdrxoC3mYQAvD_BwE> - https://www.bayshore.ca/services/government/alternative-level-of-care/ |
| ON/  Reintegration Care Unit/  Les Centres d’Accueil Héritage (CAH)/ (2022) (109) | Facility | <https://www.caheritage.org/en/our-services/transitional-care/#:~:text=33%2C%20Hahn%20Place%20·%20Toronto%20ON,·%20Tel%3A%20416%20365%E2%80%913350>   - <https://www.caheritage.org/en/reintegrationcareunit/> - <https://www.caheritage.org/en/our-services/overview-of-cah-services-3/> |
| ON/  Hillcrest Reactivation Centre/  University Health Network/ (2022) (110) | Facility | <https://www.uhn.ca/PatientsFamilies/Health_Information/Health_Topics/Documents/Welcome_To_Hillcrest_Reactivation_Centre.pdf>   - <https://www.uhn.ca/OurHospitals/Hillcrest> |
| ON/  Binbrook Transitional Care Bed Program/  AbleLiving/ (2022) (111) | Facility | <https://ableliving.org/programs/#transitional-wellness>   - https://www.hnhbhealthline.ca/displayService.aspx?id=193553 |
| **Province/**  **Program Name/ Author/  (Year)** | **Model of Care** | **Source Links** |
| ON/  Helping Hands Orillia Transitional Bed Program/ Helping Hands/ (2022) (112) | Facility | <https://helpinghandsorillia.ca/transitional-bed-service/>   - <https://helpinghandsorillia.ca> - <https://www.nsmhealthline.ca/displayservice.aspx?id=206201> |
| ON/  Transitional Care Units/  VHA Home Health Care/ (2022) (113) | Facility | <https://www.vha.ca/integrated-solutions/transitional-care-units/>   - https://www.vha.ca/services/ |
| ON/  Transitional Care Sites/  Canes Community Care/ (2020) (114) | Facility | https://www.canes.on.ca/services/transitional-care |
| ON/  RVH-IOOF Patient Flow Program/  Royal Victoria Regional Health Centre/ (2021) (115) | Facility | <https://www.rvh.on.ca/areas-of-care/rvh-ioof/> |
| ON/  Transitional Behavioral Support Unit/  Baycrest/  (2023) (116) | Facility | <https://www.baycrest.org/Baycrest/Healthcare-Programs-Services/Programs/Behavioural-Support-for-Seniors-Program>   - <https://www.baycrest.org/Baycrest_Centre/media/content/images/TBSU_FactSheet_FINAL_Nov14.pdf> - <https://www.camh.ca/-/media/files/community-resource-sheets/older-adults-resources-pdf.pdf> - <https://www.baycrest.org/Baycrest_Centre/media/content/images/LTC_BSOT_FactSheet_FINAL_Nov13.pdf> - <https://brainxchange.ca/BSO/2020-2021-Annual-Report.aspx> |
| ON/  Queen’s Estate Transitional Care Rehab Unit/  Queens Estate/ (2019) (117) | Facility | https://www.nygh.on.ca/data/2/rec_docs/3394_TCU-Brochure-NYGH-January-2019.pdf |
| ON/  Yee Hong Centre for Geriatric Care/ Indus Community Services/  (2018) (118) | Facility | <https://trilliumhealthpartners.ca/newsroom/Documents/2018/Indus-THP-Yee-Hong-News-Release-April-2018.pdf>   - https://induscs.ca/wp-content/uploads/2018/08/Indus-Current-Summer-2018-Final.pdf |
| ON/  Sunrise Short Stay Support/  Eastern York Region North Durham Sunrise Senior Living/ (2022) (119) *Facilities exist in QC, ON | Facility | <https://static1.squarespace.com/static/5ef00d2f7bc5bf6a756dbe09/t/6266845ee2b6216656b1cd5c/1650885730126/21-7267+Sunrise+Short+Stay+Support+Brochure+2.pdf> |
| ON/  Perley Health Sub-Acute Care for Frail Elderly [SAFE]/  Perley Health/ (2021) (120) | Facility | <https://www.perleyhealth.ca/safe>   - <https://ottawacitizen.com/opinion/columnists/hoffer-and-roth-ottawa-tries-a-safe-way-to-address-hallway-health-care> - <https://www.perleyhealth.ca/safe-study-released> - <https://www.perleyhealth.ca/> - https://www.perleyhealth.ca/upload/documents/oct27-nr-safe-4.pdf |
| ON/  Caledon Transitional Care Program/ Caledon Community Services/ (2022) (121) | Facility | <https://www.centralwesthealthline.ca/displayservice.aspx?id=111927>   - <https://ccs4u.org/health/transitional-care> - <https://ccs4u.org/health/transitional-care/tcc-eligibility-criteria> - https://211central.ca/record/69806937/ |
| **Province/**  **Program Name/ Author/  (Year)** | **Model of Care** | **Source Links** |
| ON/  Transitional Care Unit: Windsor Retirement Residence/  Kingston Health Sciences Centre/ (2021) (122) | Facility | https://kingstonhsc.ca/khscconnect/news/transitional-care-unit-grows-again |
| ON/  Rubidge Retirement Residence Transitional Care Unit/  Peterborough Regional Health Centre/ (2020) (123) | Facility | <https://www.prhc.on.ca/wp-content/uploads/2020/09/PRHC-Strat-Plan-September-2020.pdf>   - https://pub-peterborough.escribemeetings.com/filestream.ashx?DocumentId=26618 |
| ON/  Rekai Centre Transitional Care Unit/  Sinai Health/  (2022) (124) | Facility | https://rekaicentre.com/tcu.html |
| ON/  Transitional Care Beds/ Niagara Gardens Retirement Manor/ (2022) (125) | Facility | https://www.hnhbhealthline.ca/displayService.aspx?id=193560 |
| British Columbia | | |
| BC/  Personalized Support & Stabilization Team Plus (PSS+) at Robert & Lily Lee Family Community Health Centre/ Vancouver Coastal Health/ (2023) (126) | Comm | https://sea-to-sky.pathwaysbc.ca/programs/3704   - <https://vch.eduhealth.ca/media/VCH/EF/EF.200.R73.pdf> - <https://www.vch.ca/en/location-service/personalized-support-stabilization-team-plus-pss-rllf-chc> |
| BC/  Transition Services Team (TST) Vancouver General Hospital/ Vancouver Coastal Health/ (2023) (127) | Hosp | https://sea-to-sky.pathwaysbc.ca/programs/2157   - <https://www.vch.ca/en/location-service/transition-services-team-vancouver-general-hospital> |
| BC/  Transition Services at Koerner Pavilion/ Vancouver Coastal Health/ (2023) (128) | Hosp | https://www.vch.ca/en/location-service/transition-services-team-koerner-pavilion |
| BC/  Convalescent Care Program/  Fraser Health/ (2023) (129) | Hosp | https://www.fraserhealth.ca/Service-Directory/Services/Seniors---Community-Services/convalescent-care#.ZEgTqOzMKZx   - <https://www2.gov.bc.ca/gov/content/health/accessing-health-care/home-community-care/care-options-and-cost/short-stay-services> |
| BC/  Convalescent Care Program/  Interior Health/  (2023) (130) | Hosp | https://www.interiorhealth.ca/health-and-wellness/home-and-community-care/convalescent-care   - <https://www.interiorhealth.ca/stories/ensuring-smooth-transition-hospital-home> - <https://www.interiorhealth.ca/health-and-wellness/home-and-community-care/help-with-living-at-home> - <https://www.interiorhealth.ca/information-for/patients-and-visitors/getting-discharged-from-hospital#determining-the-best-care-option-for-you> |
| **Province/**  **Program Name/ Author/  (Year)** | **Model of Care** | **Source Links** |
| BC/  Providence Care Centre/  Providence Care/ (2022) (131) | Hosp | https://providencecare.ca/providence-transitional-care-centre/   - <https://providencecare.ca/providence-transitional-care-centre/inpatient-care-services/> - <https://providencecare.ca/providence-transitional-care-centre/inpatient-care-services/discharge-leaving/> - https://providencecare.ca/providence-transitional-care-centre/inpatient-care-services/inpatient-meals/ |
| BC/  Glengarry Transitional Care Unit/ Island Health/  (2023) (132) | Hosp | https://www.islandhealth.ca/our-locations/mental-health-substance-use-locations/glengarry-transitional-care-unit-mental   - https://www.islandhealth.ca/our-locations/long-term-care-locations/glengarry |
| BC/  Short Term Enablement and Planning Suites (STEPS)/ Island Health/ (2022) (133) | Facility | https://www.islandhealth.ca/news/stories/innovative-program-allows-cowichan-residents-transition-temporarily-between-hospital-and-home   - <https://www.islandhealth.ca/news/news-releases/cowichan-steps-program-winner-prestigious-national-award> - <https://www.cerner.com/ca/en/client-achievements/island-health-succeeds-with-hospital-at-home> |
| BC/  Evergreen House/ Vancouver Coastal Health/ (2023) (134) | Facility | <https://oasis.vch.ca/media/EvergreenHouse_WelcomeBooklet.pdf>   - <https://www.vch.ca/en/location/evergreen-house> |
| BC/  Glenwood Care Centre/  Fraser Health/ (2023) (135) | Facility | https://www.fraserhealth.ca/news/2023/Dec/fraser-health-opens-community-transitional-housing--to-support-vulnerable-people   - <https://www.fraserhealth.ca/Service-Directory/Service-At-Location/0/0/glenwood-care-centre#.ZEgUxezMKZx> |
| Alberta | | |
| AB/  Alberta Health Services Self-Managed Care Program/  Harmony Caregiving/ (2023) (136) | Comm | https://harmonycaregiving.com/hospital-to-home-care-self-managed-care/ |
| AB/  Post-Hospital Care/ Comfort Keepers/ (2023) (137) | Comm | https://www.comfortkeepers.ca/reddeer/home-care-in-home-care-rocky-mountain-house-ab/   - <https://www.comfortkeepers.ca/reddeer/transition-services/> - <https://www.comfortkeepers.ca/post-hospital-care/> |
| AB/  Post-Hospital Care/ Care West AB/ (2020) (138) | Comm | https://carewest.ca/dir/wp-content/uploads/2020/01/RCTP-brochure-for-all-Jan.-2020.pdf   - <https://carewest.ca/dir/wp-content/uploads/2020/01/Carewest-Today-2020-for-web.pdf> - <https://carewest.ca/programs-and-services/> - <https://carewest.ca/dir/wp-content/uploads/2016/03/Carewest-GMH-Brochure-2015-July.pdf> - <https://carewest.ca/dir/wp-content/uploads/2018/12/Carewrite-January-to-March-2019-revised-for-web.pdf> - <https://carewest.ca/dir/wp-content/uploads/2018/11/Carewest-Strategic-Plan-2018-2021-1.pdf> - <https://carewest.ca/dir/wp-content/uploads/2020/01/Glenmore-Site-and-Services.pdf> - <https://carewest.ca/carewest-glenmore-park/> |
| AB/  Hope 4 Life/  Hope 4 Life Home Care/ (2017) (139) | Comm | http://hope4life.ca/services/home-care/   - <https://hope4life.ca/about-us/> - <https://hope4life.ca/services/addiction-counseling/> |
| AB/  Rehabilitation and Enhanced Community Transition Program/ Home Care Assistance/ (2022) (140) | Facility | https://www.homecareassistanceedmonton.ca/post-hospital-home-care/   - <https://www.homecareassistanceedmonton.ca/live-in-or-24-hour-home-care/> - <https://www.homecareassistanceedmonton.ca/skilled-nursing-care/> |
| **Province/**  **Program Name/ Author/  (Year)** | **Model of Care** | **Source Links** |
| Saskatchewan | | |
| SK/  Kensington Transitional Beds/  Kensington Gentle Care Home/ (2023) (141) | Facility | <https://kensingtongentlecare.com>   - <https://sk.211.ca/services/kensington-transitional-beds/transitional-beds/> |
| SK/  Convalescent Care/ William Booth Special Care Home/ (2021) (142) | Facility | <http://williamboothregina.ca/wp-content/uploads/2024/03/2024-Convalescent-Resident-Family-Handbook.pdf>   - <https://williamboothregina.ca/convalescent-care/> - <https://www.saskhealthauthority.ca/facilities-locations/william-booth-special-care-home> |
| Manitoba | | |
| MB/  Priority Home Rapid Response Nursing Team/ Winnipeg Regional Health Authority  (2017) (143) | Comm | <https://professionals.wrha.mb.ca/files/priority-home-rrn-client-info-sheet-e.pdf> |
| MB/  Priority Home/ Winnipeg Regional Health Authority/ (2017) (144) | Comm | <https://professionals.wrha.mb.ca/files/priority-home-hospital-faq.pdf>   - <https://professionals.wrha.mb.ca/priority-home/> - <https://wrha.mb.ca/2017/09/20/wrha-announces-priority-home/> |
| MB/  Prairie Mountain Health Transitional Care/ Prairie Mountain Health/ (2023) (145) | Hosp | <https://prairiemountainhealth.ca/about-us/>   - https://prairiemountainhealth.ca/our-locations/birtle/ |
| MB/  Misericordia Transitional Care Unit (Restorative Care)/  Misericordia Health Centre/ (2023) (146) | Hosp | <https://misericordia.mb.ca/wp-content/uploads/factsheet-TCU.pdf>   - <https://misericordia.mb.ca/programs/long-term-care/transitional-care-unit/> - <https://misericordia.mb.ca/wp-content/uploads/TCU-welcome.pdf> |
| MB/  Victoria Hospital- Geriatric Rehabilitation Unit/ Victoria hospital/ (2020) (147) | Hosp | <https://issuu.com/willowonline/docs/victoria_general>   - <https://vgh.mb.ca/patients/programs/geriatric-rehabilitation/> |
| MB/  Rehabilitation Geriatric Services- Seven Oaks General Hospital/ Seven Oaks General Hospital/ (2023) (148) | Hosp | <https://sogh.ca/files/rehabgeribrochureweb.pdf>   - <https://sogh.ca/services/rehabilitation-geriatric-services/> |
| MB/  Deer Lodge- Geriatric Assessment and Rehabilitation Program/ Deer Lodge Centre/ (2023) (149) | Hosp | https://deerlodge.mb.ca/short-stays-at-dlc/geriatric-assessment-and-rehabilitation/ |
| Quebec | | |
| QC/  McGill University Health Centre Transitional care Unit/  McGill University Health Centre/ (2023) (150) | Hosp | <https://muhc.ca/transition/profile/transition-care-service>   - https://muhc.ca/department-medicine |
| **Province/**  **Program Name/ Author/  (Year)** | **Model of Care** | **Source Links** |
| QC/  Jeffery Hale Saint Brigid’s Geriatrics Unit -Functional Rehabilitation Transition Unit/ Jeffery Hale Saint Bridges/ (2023) (151) | Hosp | https://www.ciusss-capitalenationale.gouv.qc.ca/jhsb/services/services-aines/utrf   - https://www.ciusss-capitalenationale.gouv.qc.ca/jhsb/services/services-aines |
| QC/  Short Term Stay Sunrise Senior Living Quebec/  Sunrise Senior Living/ (2022)  *Facilities exist in ON, QC (152) | Facility | https://www.sunriseseniorliving.ca/lp/quebec |
| New Brunswick | | |
| NB/  New Brunswick Extra Mural Program/ Medavie Health Services NB/ (2023) (153) | Comm | https://extramuralnb.ca/en/who-we-are/our-values/   - https://extramuralnb.ca/en/how-we-are-doing/ |
| NB/  Rapid Rehabilitation and Reablement/ Social Supports NB/ (2023) (154) | Comm | https://socialsupportsnb.ca/en/simple_page/rapid-rehabilitation-and-reablement |
| NB/  Transitional Living Suites / Horizon Health Network NB/ (2023) (155) | Hosp | https://horizonnb.ca/horizon-services/provincial-program/stan-cassidy-centre-for-rehabilitation/services/adult-services/ |
| Prince Edward Island | | |
| PEI/  Caring for Older Adults in the Community [COACH] Program/ Government of Prince Edward Island/ (2022) (156) | Comm | <https://www.princeedwardisland.ca/en/information/health-pei/caring-for-older-adults-in-the-community-and-at-home-coach-program>   - <https://www.healthcareexcellence.ca/en/what-we-do/all-programs/advancing-frailty-care-in-the-community/caring-for-older-adults-in-the-community-and-at-home/#:~:text=at%20Home%20(COACH)-,Caring%20for%20Older%20Adults%20in%20the%20Community%20and%20at%20Home,return%20home%20from%20hospital%20sooner> - https://www.healthcareexcellence.ca/media/zocn2fiq/coach-innovationprofile-e-final-ua.pdf |
| Nova Scotia | | |
| NS/  Victoria General Hospital Transitional Care Unit/ Nova Scotia Health Authority/ (2023) (157) | Hosp | https://www.cdha.nshealth.ca/recreation-therapy/internship-opportunities |
| Newfoundland and Labrador | | |
| NL/  Central Health Restorative Care/ Central Health/ (2023) (158) | Hosp | <https://www.centralhealth.nl.ca/grand-falls>   - <https://www.centralhealth.nl.ca/restorative-care> - https://www.centralhealth.nl.ca/_files/ugd/d55165_9510db0742774d4cae3cb941dc0fa41b.pdf |
| Northwest Territories | | |
| NT/  Norman Wells Transitional Care/ Northwest Territories Health Authority/ (2019) (159) | Facility | <https://www.nthssa.ca/sites/nthssa/files/resources/nthssa2018-19-annual-report-final.pdf>   - <https://www.mytruenorthnow.com/31909/news/new-health-centre-opens-in-norman-wells/> - https://www.gov.nt.ca/en/newsroom/news/grand-opening-sahtu%CC%81-gotin%C3%A9-regional-health-and-social-services-centre-and-sahtu%CC%81-dene |
| **Province/**  **Program Name/ Author/  (Year)** | **Model of Care** | **Source Links** |
| Yukon | | |
| YT/  Bridge-to-Home/ Government of Yukon/ (2023) (160) | Comm | <https://www.healthcareexcellence.ca/en/news/2023-06-16-new-bridge-to-home-bc-and-yukon-teams-will-help-improve-care-transitions-from-hospital-to-home/>   - https://www.healthcareexcellence.ca/en/what-we-do/all-programs/bridge-to-home/?utm_source=cfhi-fcass.ca&utm_medium=CTA&utm_campaign=Migration&utm_content=BridgetoHome |
